# Structural elaboration of dicyanopyrazine: towards push–pull molecules with tailored photoredox activity†

**DOI:** 10.1039/c9ra04731j

**Published:** 2019-07-31

**Authors:** Zuzana Hloušková, Milan Klikar, Oldřich Pytela, Numan Almonasy, Aleš Růžička, Veronika Jandová, Filip Bureš

**Affiliations:** Institute of Organic Chemistry and Technology, Faculty of Chemical Technology, University of Pardubice Studentská 573 Pardubice 53210 Czech Republic filip.bures@upce.cz; Department of General and Inorganic Chemistry, Faculty of Chemical Technology, University of Pardubice Studentská 573 Pardubice 53210 Czech Republic

## Abstract

As an extension of the successful dicyanopyrazine photoredox catalysts, a series of X-shaped push–pull molecules with a systematically altered structure were designed and facilely synthesized; their structure–property relationship was elucidated in detail *via* experimental as well as theoretical calculations. Dicyanopyrazines are proven to be powerful photoredox catalysts with a push–pull arrangement that allows facile property tuning by interchanging a particular part of the D–π–A system. Changing the mutual position of the cyano acceptors and the methoxy, methylthio and thienyl donors as well as modifying the linker allowed wide tuning of the fundamental properties of the catalysts. Contrary to the currently available organic photoredox catalysts, we provided a series of catalysts based on a pyrazine heterocyclic scaffold with easy synthesis and further modification, diverse photoredox characteristics and wide application potential across modern photoredox transformations. The photoredox catalytic activities of the target catalysts were examined in a benchmark cross-dehydrogenative coupling and novel and challenging annulation reactions.

## Abstract 2

X-shaped push–pull molecules based on dicyanopyrazine were designed, synthesized and experimentally/theoretically investigated. They proved to be tunable photoredox catalysts in selected chemical transformations.

## Graphical abstract

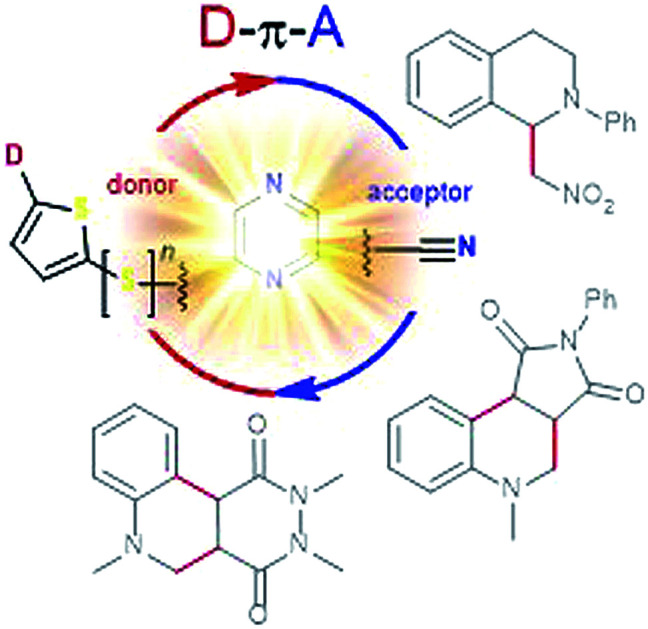

## Introduction

The idea of using visible light (VL) as a stimulant to promote organic transformations is more than a hundred years old, and G. Ciamician was the first researcher to utilize VL in photochemical reactions.^1^ Nowadays, the transformation of solar energy into energy of a chemical bond is a burgeoning area of photochemistry.^2^ This process, inspired by the chlorophyll mechanism of action, is realized by a photoredox catalyst/catalysis (PC) under laboratory conditions. An essential prerequisite of PCs is their ability to undergo single electron transfer (SET) either from or to the catalyst/substrate. In the last decade, it has been verified that photoredox catalysis is a very efficient tool in a number of chemical transformations. They also include new and unprecedented reactions, which provide novel compounds both for academia and industry. The portfolio of common VL-catalysed organic transformations includes but is not limited to oxidations,^3^ reductions,^4^ sp^2^–sp^2^ (ref. 5)/sp^2^–sp^3^ (ref. 6)/sp^3^–sp^3^ (ref. 7) C–C as well as C–S (ref. 8), C–N (ref. 9), C–O (ref. 10) and C–P (ref. 11) bond formation. VL photoredox catalysis has also been well utilized in cycloaddition reactions.^12^ In the last two decades, the research on PC has mostly been focused on the application of transition metal complexes. The polypyridyl complexes of ruthenium and iridium represent the most prominent PCs with superior properties and wide applications.^13^ Dual catalysis is another significant extension of the original PC, which utilizes combined complexes of gold/iridium,^14^ gold/ruthenium^15^ and nickel/iridium.^16^ The main advantages of these complexes include their high photoredox activity, stability, facile synthesis and further modification. However, they are generally expensive, and the presence of toxic heavy metals is undesirable.

On the other hand, organic dyes represent a very promising group of rising photocatalysts. They include the well-known xanthene dyes such as fluorescein,^17^ Eosin Y,^18^ Eosin B,^19^ rose Bengal,^20^ acridinium^21^ and pyrilium salts,^22^ which are readily available, cheap and possess good catalytic activity; nevertheless, the tuning of their properties is limited. Therefore, the attention of chemists has recently been drawn to the synthesis of organic catalysts with tunable properties.^23^ The positive aspects of organic PCs include low price, accessibility, stability and solubility, but mostly their property tuning towards a given photoredox process.^24^ Organic PCs should possesses essential properties as follows: (i) absorption of light within the UV/Vis wavelength range, (ii) absorption maximum overlapping the emission band of the light source and (iii) capability to undergo SET or energy transfer. Good solubility in the reaction medium, inactivity towards substrate/product of the catalysed reaction and low catalytic loadings are also desirable.^24^

**Fig. 1:**
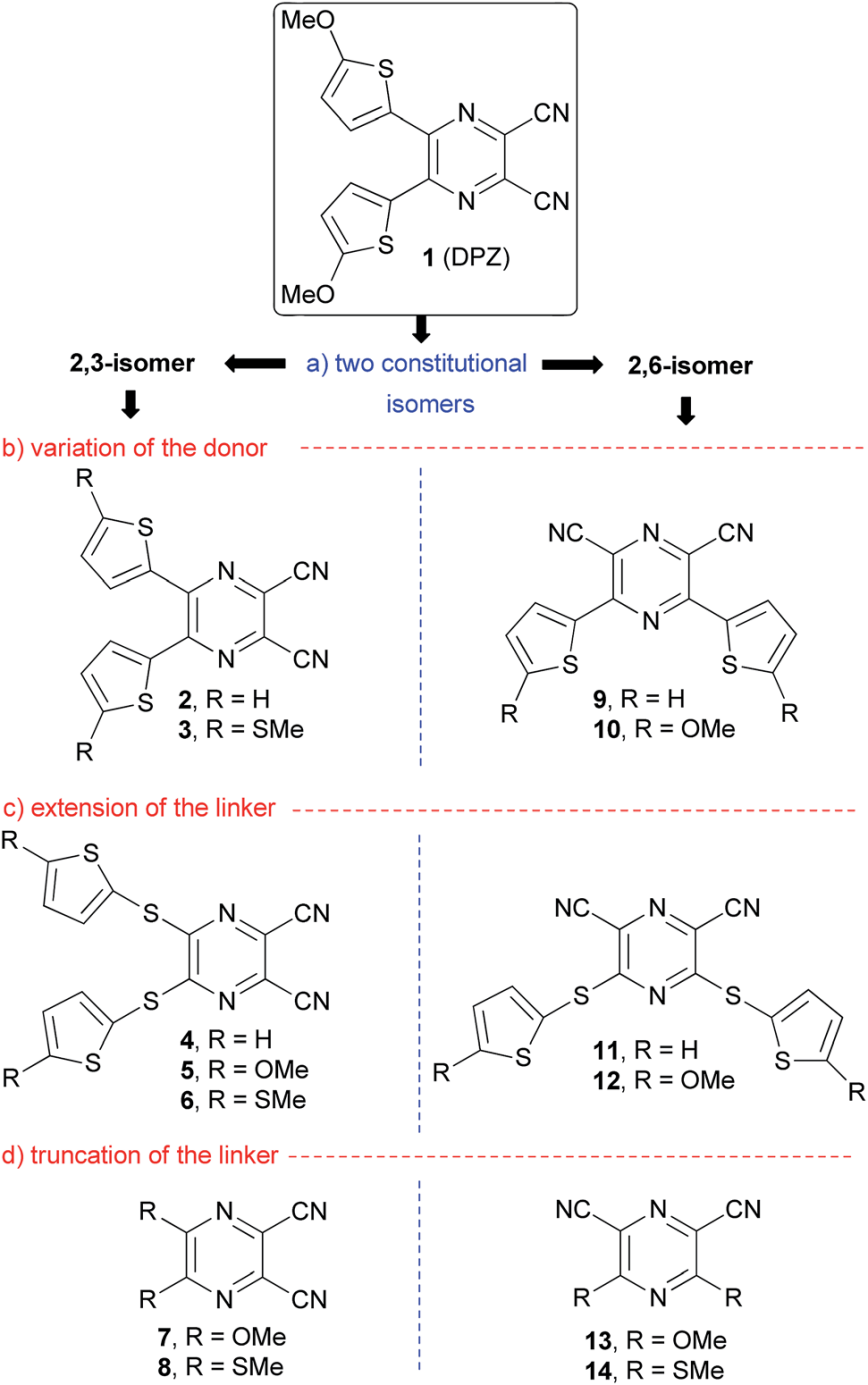
Structural tuning of the DPZ push–pull molecules.

In 2012, push–pull molecules based on 4,5-disubstituted pyrazine-2,3-dicarbonitrile (dicyanopyrazine, DPZ) have been synthesized in our research group.^25^ These X-shaped molecules proved superior CT-chromophores (charge-transfer, CT) with nonlinear optical properties. Further structural tuning resulted in DPZ derivative 1 bearing two 5-methoxythienyl donors, which showed an excellent performance as a photoredox catalyst. Subsequently, DPZ 1 was successfully applied in a benchmark cross-dehydrogenative coupling (CDC) reaction,^26^ oxidation, oxidative hydroxylation, reductive dehalogenation,^24^ chemodivergent radical cascade reactions between *N*-tetrahydroisoquinolines and *N*-itaconimides,^27^ pH-controlled photooxygenation of indoles^28^ and enantioselective oxidative C(sp^3^)–H olefinations.^29^ Its catalytic activity in the aforementioned reactions was outstanding, and in many cases superior to that of other organometallic catalysts. Hence, our further synthetic attempts were focused on the structural modification of 1, especially in terms of introducing an additional 2,5-thienylene ring.^30^ In the present study, we present a thorough structural elaboration of the DPZ family of photoredox catalysts (Fig. 1). Considering that the original DPZ 1 is a D–π–A push–pull molecule,^31,32^ the structural variation may in principle involve the alternation of the acceptor (dicyanopyrazine), π-linker (2,5-thienylene) and the donor (methoxy group).

Fig. 1 summarizes the structural changes carried out within this work, which according the particular part of the DPZ D–π–A system include:

(a) Alternation of the acceptor, especially in terms of mutual position of the CN groups: two series of disubstituted pyrazine-2,3/2,6-dicarbonitrile isomers.

(b) Utilization of OMe and SMe groups as electron donors and unsubstituted derivatives (R = H) as reference compounds.

(c) Introduction of an additional sulfidic (–S–) linker, which separates the electron donor from the pyrazine acceptor.

(d) Removal of the 2,5-thienylene π-linker and direct connection of electron donors to the pyrazine ring.

All the new DPZ derivatives 2–14 were spectrally characterized, and their fundamental properties were further investigated by X-ray analysis, electrochemistry, electronic absorption and emission spectra, which were supported by DFT calculations. DPZs 1–14 were subsequently tested as photoredox catalysts in benchmark CDC and annulation reactions and their thorough structure–catalytic activity relationships were elucidated.

## Results and discussions

### Synthesis

**Scheme 1:**
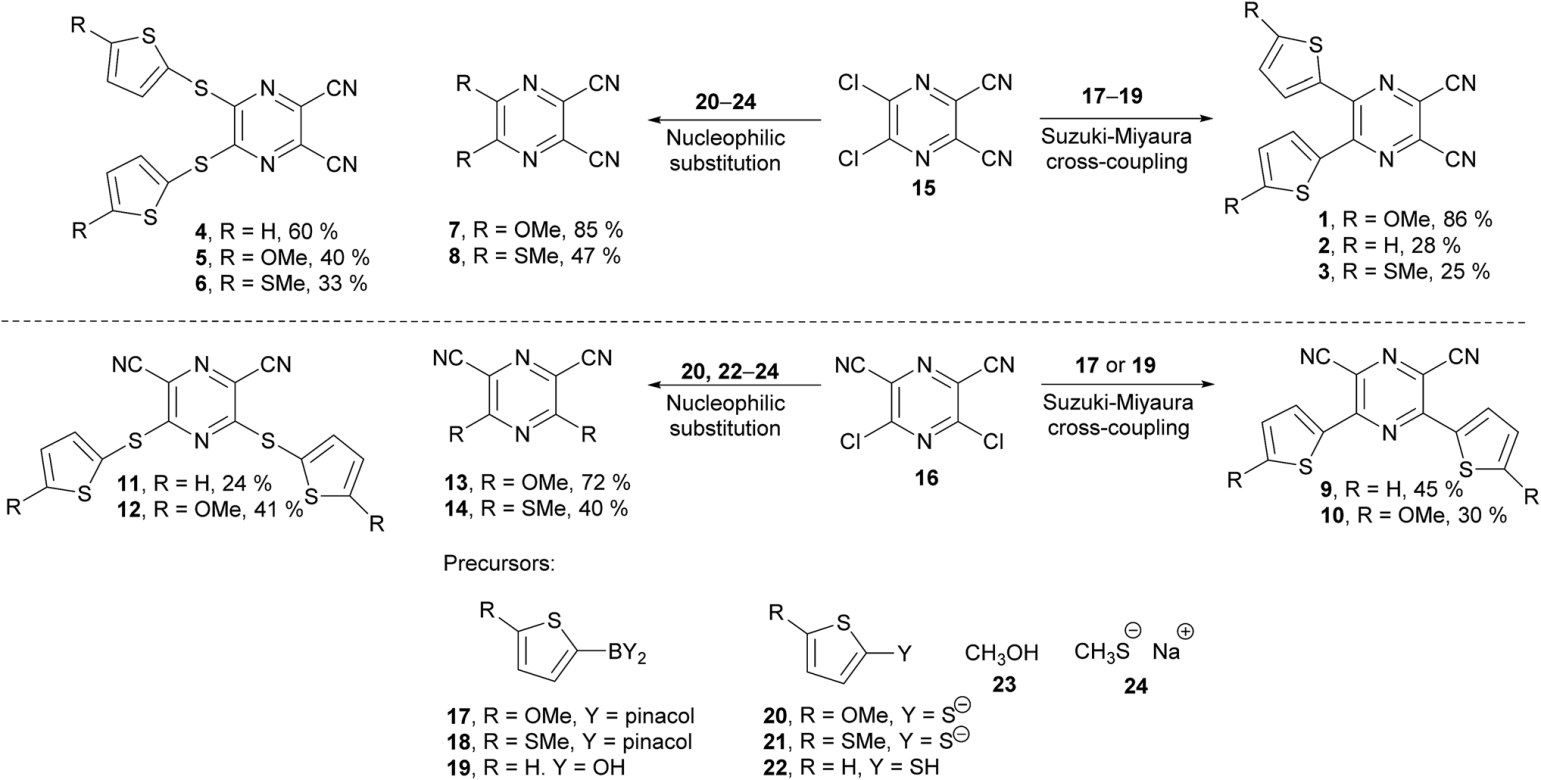
General reaction scheme leading to the target push–pull pyrazines 1–14.

**Scheme 2:**
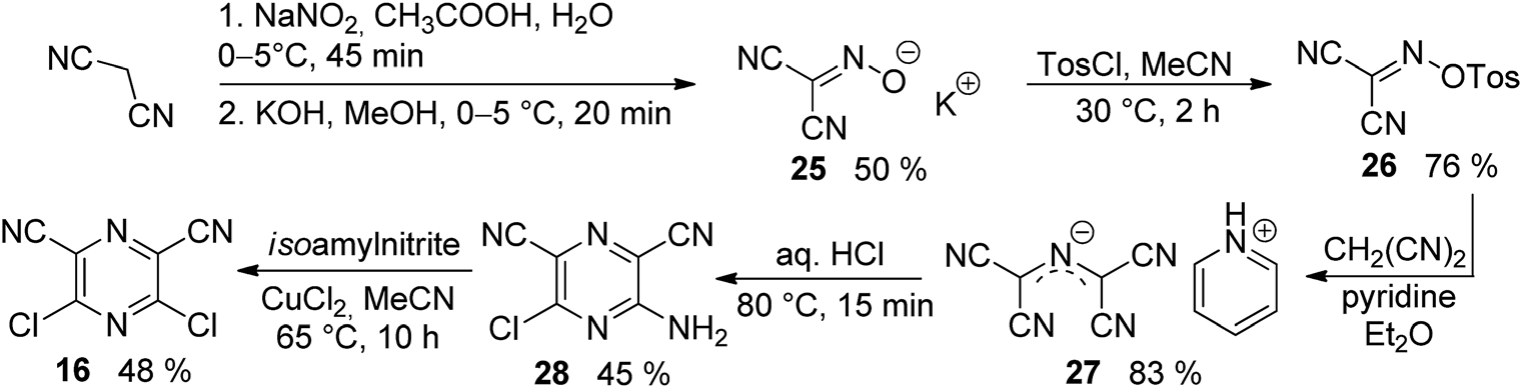
Synthesis of 3,5-dichloropyrazine-2,6-dicarbonitrile 16.

The synthesis of the original DPZ derivative 1 was published previously.^24^ The synthetic strategy (Scheme 1) towards target molecules 2–14 utilized the commercially available 5,6-dichloropyrazine-2,3-dicarbonitrile 15, and its isomer 3,5-dichloropyrazine-2,6-dicarbonitrile 16, which was prepared in a five-step reaction sequence starting from malononitrile (Scheme 2). Its gradual nitrosation,^33^ tosylation,^34^ nucleophilic substitution using another malononitrile,^35^ acid-catalysed cyclization^35^ and final Sandmeyer reaction^36^ afforded 16 in 22% overall yield. More synthetic details are provided in the ESI.†

The chloro atoms in both derivatives 15 and 16 could be replaced in terms of nucleophilic substitution or Suzuki–Miyaura cross-coupling reaction (Scheme 1). Unfortunately, the same set of reactions carried out on the third possible isomer, 3,6-dibromopyrazine-2,5-dicarbonitrile,^37^ failed. All attempts to further optimize/vary the reaction conditions were unsuccessful. Target molecules 1–3 and 9–10, bearing a thiophene ring directly attached to the pyrazine core, were synthesized by Suzuki–Miyaura cross-coupling reactions between dichloropyrazine 15 or 16 and appropriate boronic acid derivative 17–19. DPZ 10 proved to be unstable during storage. All PCs were isolated by column chromatography in yields in the range of 25–86%. Boronic acid pinacol esters 17 and 18 were synthesized as shown in the ESI.†

Compounds 4–8 and 11–14 were prepared from 15 or 16 and nucleophiles 20–24 in yields in the range of 24–72%. Derivatives 7, 8, and 13 were reported previously.^38–40^ Thiolates 20 and 21 were generated *in situ* from the corresponding thiophene derivative and its gradual lithiation and reaction with sulphur, with more details presented in the Experimental section.

### X-ray analysis

**Fig. 2:**
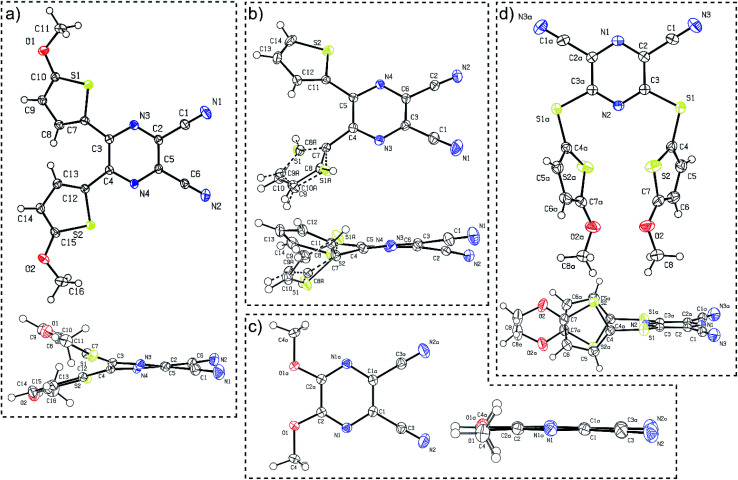
ORTEP representations of the DPZ derivatives (a) 1 (CCDC 1553203), (b) 2 (CCDC 1553204), (c) 7 (CCDC 1897538) and (d) 12 (CCDC 1897539). Vibrational ellipsoids obtained at 150 K are shown at the 50% probability level, *R* = 0.04–0.05. Top and side views are provided.

Suitable single crystals of compounds 7 and 12 were prepared by slow evaporation and hexane diffusion of their DCM solutions. X-ray analyses of parent compounds 1 and 2 were published previously.^30^Fig. 2 shows the ORTEP plots of all the aforementioned derivatives, and the ESI† presents more details and X-ray analysis of intermediate 27. As can be seen from the dimethoxy derivative 7, the parent DPZ adopts an almost planar arrangement with a torsion angle below 1.5° (see the side view). However, both thiophene rings in 1 and 2 showed considerable twist with a torsion angle between 15–35°. As a result, the dicyanopyrazine moiety in 1 and 2 is curved with inner torsion angles between 5–15°. In contrast to the DPZ 2,3-isomers (1, 2 and 7), the 2,6-isomer 12 with a sulfidic linker adopts a completely different arrangement. Although the DPZ acceptor is planar, both 5-methoxythiophenes appended at positions 3 and 5 are perpendicularly localized below the DPZ ring. The orientation of both rings is reverse and parallel with a mutual distance of about 4 Å. The D–A interaction, in particular the push–pull molecules, was estimated through the aromaticity Bird index of the pyrazine and thiophene rings.^41^ The pyrazine rings in 1, 2, 7 and 12 possess *I*_6_ values of 81.8, 84.4, 71.2 and 90.8. A, respectively, in comparison to the unsubstituted pyrazine (*I*_6_ = 88.8), revealing a higher bond length alternation, and thus higher ICT in the 2,3-isomeric DPZs 1, 2 and 7.

The 2,6-isomer 12 possesses higher aromaticity than pyrazine, and therefore, the extent of the intramolecular charge transfer (ICT) is lower. This is because the additional sulfidic linker: (i) is generally a weaker electron donor than the methoxy group (such as in 7),^31^ (ii) separates the 5-methoxythiophene moiety from the pyrazine acceptor and disallows their mutual D–A interaction and (iii) also delocalizes its lone electron pairs partially to the appended thiophene. The average Bird index of the thiophene rings in 1, 2 and 12 are 61.6, 64.3 and 59.8, respectively. Taking *I*_5_ = 66 as a reference value for the unsubstituted thiophene, the thiophene rings in 12 seem to be the most polarized, but are not directly conjugated to the DPZ acceptor. The methoxy groups appended at the thiophene as in 1 also cause significant polarization compared to the unsubstituted derivative 2.

### Photophysical properties

The colour of the DPZ derivatives 1–14 ranges from yellow to red. Their fundamental photophysical properties were measured in acetonitrile (Table 1) and the less polar dichloromethane and 1,4-dioxane (Table S1 in the ESI†). However, only slight solvatochromism of the absorption spectra was observed with Δ*λ*^A^_max_ = 0–21 nm. The emission spectra of 1, 2, 9 and 10 with an enlarged π-system showed positive solvatochromism with Δ*λ*^F^_max_ = 0–46 nm, but no solvatochromism was observed for 7–8 and 13–4 with a truncated π-system.

**Table 1:** Experimentally obtained photophysical and electrochemical parameters of DPZ derivatives 1–14

Comp.	Photophysical data^a^					Electrochemical data^a^						
						Ground state					Excited state	
	*λ* ^A^_max_ [nm eV^−1^]	*λ* ^F^_max_ [nm eV^−1^]	*q* ^F^	Stokes shift [cm^−1^ eV^−1^]	*E*_0,0_ b [eV]	*E*_p(ox1)_ c [V]	*E*_p(red1)_ c [V]	Δ*E*^d^ [V]	*E* ^el^ e(HOMO) [eV]	*E* ^el^ e(LUMO) [eV]	*E*_ox_*^f^ [V]	*E*_red_*^f^ [V]
1	440/2.82	571/2.17	<0.02	5200/0.65	2.50	1.32	−1.14	2.46	−5.79	−3.33	−1.18	1.36
2	379/3.27	488/2.54	<0.02	5900/0.73	2.91	1.75	−1.12	2.87	−6.22	−3.35	−1.16	1.79
3	443/2.79	552/2.25^g^	<0.02^g^	4100/0.51^g^	2.22^g^	1.32	−1.01	2.33	−5.79	−3.46	−0.90^g^	1.21^g^
4	315/3.94	—	—	—	—	1.82	−0.97	2.79	−6.29	−3.50	—	—
5	279/4.44	—	—	—	—	1.31	−1.00	2.31	−5.78	−3.47	—	—
6	307/4.04	—	—	—	—	1.25	−0.95	2.20	−5.72	−3.52	—	—
7	278/4.46	349/3.55	0.024	7300/0.91	4.00	—	−1.53	—	—	−2.94	—	2.47
8	323/3.84	409/3.03^g^	—	6300/0.78^g^	2.97^g^	—	−1.23	—	—	−3.24	—	1.74^g^
9	343/3.62	462/2.68	<0.02	7600/0.93	3.15	—	−1.04	—	—	−3.43	—	2.11
10	389/3.19	562/2.21	<0.02	4500/0.98	2.70	1.57	−1.15	2.72	−6.04	−3.32	−1.13	1.55
11	363/3.42	—	—	—	—	1.79	−0.97	2.76	−6.26	−3.50	—	—
12	351/3.53	—	—	—	—	1.34	−1.00	2.34	−5.81	−3.47	—	—
13	322/3.85	363/3.42	0.18	3600/0.43	3.64	—	−1.41	—	—	−3.06	—	2.23
14	371/3.34	417/3.00	<0.02	3000/0.37	3.17	—	−1.16	—	—	−3.31	—	2.01

a Measured in acetonitrile.

b Excited state energy; calculated as the midpoint between the absorption and emission maxima (ref. 23).

c *E*_p(ox1)_ and *E*_p(red1)_ are the peak potentials of the first oxidation and reduction, respectively; all potentials are given *vs.* SSCE.

d Δ*E* = *E*_p(ox1)_ − *E*_p(red1)_ (electrochemical gap).

e *E* ^el^(HOMO/LUMO) = *E*_p(ox1/red1)_ + 4.429 (in AcCN *vs.* SCE) + 0.036 (difference between SCE (0.241 *vs.* SHE) and SSCE (0.205 *vs.* SHE)).

f Excited-state redox potentials in acetonitrile calculated as follows: *E*_ox_* = *E*_p(ox1)_ − *E*_0,0_ and *E*_red_* = *E*_red_ + *E*_0,0_ (ref. 23).

g Measured/calculated using emission maxima obtained in DCM.

**Fig. 3:**
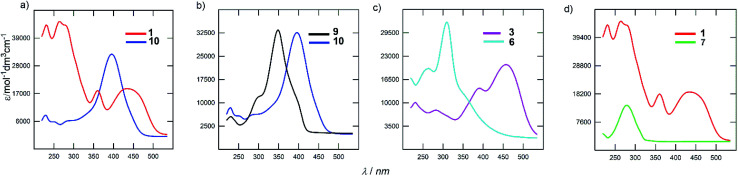
UV/Vis absorption spectra of selected DPZ derivatives.

The electronic absorption spectra of the representative derivatives are shown in Fig. 3. The longest-wavelength absorption maxima appear within a wide range of 278 to 443 nm and is clearly a function of the chromophore structure. Although the spectrum of the parent DPZ 1 contains two well-developed CT-bands, isomer 10 showed a single peak located between the two peaks of 1 (Fig. 3a). This observation obeys the Frenkel exciton model of multipodal push–pull chromophores^42^ and distinguishes the quadrupolar and linear nature of 1 and 10 (similarly to the pair 2 and 9), respectively. Thus, the mutual position of the donors and acceptors appended at the pyrazine core affects the shape and position of the bands significantly. This structural variation also dramatically affects the molar extinction coefficient, which dropped by two times.

Fig. 3b demonstrates the effect of the peripheral donor groups on the representative DPZ derivatives 9 and 10, both with a single narrow absorption peak. Upon going from 9 to 10, and thus two methoxy groups attached, the longest-wavelength absorption maximum shifts bathochromically by almost 50 nm. Further replacement of OMe with SMe groups, for instance in 13/14 or 7/8, is accompanied by a similar red shift. The same trend was also observed in the well-developed series 2/1/3 (H/OMe/SMe). However, the increasing donating ability of the R-substituents in subseries 4–6 or 11–12 has the opposite effect, which reflects a non-conjugated arrangement due to the sulfidic bridge. Its impact can further be demonstrated by comparing representative chromophores 3 and 6, both with SMe peripheral donors (Fig. 3c). The latter showed a substantially blue shifted spectrum by 136 nm. Truncation of the π-system has very similar effect, as shown for chromophores 1 and 7 in Fig. 3d. The CT-band of 7 is positioned hypsochromically by 162 nm in comparison to DPZ 1.

As can be seen from the emission data in Table 1, DPZ derivatives 1–14 are generally not or only weakly emissive, with fluorescence maxima *λ*^F^_max_ in the range of 363 to 571 nm. Only 7 and mainly 13 showed slight fluorescence with quantum yields exceeding 2%. All the derivatives bearing a sulfidic linker (4–6 and 11–12) proved to be not emissive.

The Stokes shifts are generally large, which implies high geometry reorganization upon excitation. The excited state energies *E*_0,0_ were derived from the position of the longest-wavelength absorption/emission maximum. These values refer to the transition between the lowest vibrational states of the first excited and ground levels.

### Electrochemistry

The first oxidation and reduction processes of chromophores 1–14 were studied by cyclic voltammetry (CV). The most important data is gathered in Table 1, and the ESI† presents the experimental set-up, conditions and measured CV diagrams. The first oxidations of 1–6 and 10–12 are all irreversible, and the first oxidations of the DPZs without a thiophene π-linker 7–9 and 13–14 are localized out of the potential window of the used solvent/electrolyte (MeCN/Bu_4_NBF_4_). On the other hand, the first reductions are irreversible, quasi- and reversible processes for DPZs 4–8/11–12, 13–14 and 1–3/9–10, respectively. The reversible reductions possess a cathodic/anodic peak separation of about 60 mV, which indicates one-electron processes. To make all the electrochemical data uniform, only the peak potentials *E*_p(ox1)_ and *E*_p(red1)_ are further considered. Inspection of the limiting currents revealed three times higher currents for the oxidations than for the reductions. This indicates that more electrons are being exchanged within the oxidation step. Although the first reduction takes place at the DPZ moiety, the first oxidation most likely involves the MeO/MeS-substituted thiophene moieties (see Fig. S20 in the ESI† for representative HOMO(−2/−1)/LUMO(+1/+2) localizations). The peak potential *E*_p(ox1)_ and *E*_p(red1)_ were found to be within the range of 1.25 to 1.82 V and −1.53 to −0.95 V, respectively. Since the half-wave potentials are not available for all the studied compounds, the *E*_p(ox1)_ and *E*_p(red1)_ values were recalculated as the HOMO/LUMO energies. The HOMO/LUMO levels and their differences (Δ*E*) are also visualized in the energy level diagram (Fig. S18 in the ESI†). When comparing the electrochemical behavior of the 2,3- and 2,6-isomers, *e.g.*1*vs.*10, the latter showed a deepened HOMO, unaltered LUMO and thus larger HOMO–LUMO gap. This electrochemical observation also corresponds to the aforementioned blue-shifted absorption spectrum of 10. The attachment and variation of the electron donors (H→OMe/SMe) significantly alters the HOMO energy. For instance, the well-developed series of DPZs 2/1/3 (H/OMe/SMe) showed a gradually reduced electrochemical HOMO–LUMO gap of 2.87/2.46/2.33 eV, which also correspond to red-shifted absorption spectra (see above). In the 2,3-DPZ isomers (*e.g.* pairs 1/5, 2/4 and 3/6), the extension with an additional sulfidic linker lowered only the LUMO, whereas both the HOMO and LUMO levels of the 2,6-isomers were affected, *e.g.* when going from 10 to 12. Overall, the insertion of the sulfidic linker reduces the electrochemical HOMO–LUMO gap. Truncation of the π-system by removing the thiophene linker is less electrochemically obvious as oxidations of the chromophores 7–8 and 13–14 are out of the potential window. The redox potentials of the excited states *E*_ox_* and *E*_red_* were calculated by combining the electrochemical peak potentials and the excited state energies *E*_0,0_ (Table 1), and these values are also visualized in Fig. S19 in the ESI.† Although the excited state oxidation potentials are almost unaltered, the principal changes are seen on the reduction potentials. Despite the limited data available, DPZs 1–14 seem to be strong oxidants with an *E*_red_* of up to 2.47 V.

### DFT calculations

Quantum chemical calculations were run with the DFT software package Gaussian, versions 09 (ref. 43) and 16 (ref. 44). The total energies of all the possible conformers of DPZ derivatives 1–14 were calculated using the DFT B3LYP/6-311g(2d,p) method and, based on the lowest total energy, the most stable conformers were chosen. The DFT B3LYP/6-311++g(3df,2p) method involving symmetry constrains was applied to optimize their ground state geometries and to calculate the corresponding radical cations, radical anions, dications, dianions and triplets in acetonitrile. The same method was used to calculate the total energy, energy of the frontier orbitals and further quantum-chemical characteristics in acetonitrile (Tables 2 and S2 in the ESI†). The electronic absorption spectra (see the ESI†), longest-wavelength absorption maxima *λ*^DFT^_max_ (Table 2) and corresponding electron transitions between the molecular orbitals were calculated using the TD-DFT method with the B3LYP/6-311++g(3df,2p) basis set (*n* states = 10).

**Table 2:** DFT-calculated parameters of DPZ derivatives 1–14^a^

Comp.	Radical cation		Ground state			Triplet state		Radical anion
	*E*˙^+^(αHOMO) [eV]	*λ* ^DFT^_max_ [nm eV^−1^]	*E* ^G^(HOMO) [eV]	*E* ^G^(LUMO) [eV]	Δ*E*^G^ [eV]	*E* ^T^(βHOMO) [eV]	*E* ^T^(αHOMO) [eV]	*E*˙^−^(αHOMO) [eV]
1	−6.55	454/2.73	−5.86	−2.94	2.92	−6.45	−4.22	−3.47
2	−7.24	402/3.08	−6.44	−3.13	3.33	−7.18	−4.55	−3.60
3	−6.45	472/2.63	−5.81	−2.99	2.81	−6.11	−4.16	−3.55
4	−6.94	337/3.68	−6.96	−2.90	4.05	−6.49	−4.40	−3.56
5	−6.50	335/3.70	−6.31	−2.88	3.44	−5.86	−4.21	−3.53
6	−6.42	363/3.42	−6.70	−2.93	3.77	−5.65	−4.18	−3.59
7	−8.51	279/4.44	−7.21	−2.65	4.56	−8.35	−4.77	−3.28
8	−7.91	340/3.65	−6.75	−2.79	3.95	−7.40	−4.61	−3.42
9	−7.77	380/3.26	−6.76	−3.06	3.70	−6.62	−4.41	−3.64
10	−7.07	420/2.95	−6.19	−2.84	3.36	−5.97	−4.15	−3.50
11	−7.13	345/3.60	−6.98	−2.92	4.07	−6.04	−4.40	−3.79
12	−7.09	342/3.63	−6.23	−2.88	3.36	−5.51	−4.20	−3.71
13	−8.84	297/4.18	−7.22	−2.65	4.57	−8.15	−4.57	−3.38
14	−8.18	350/3.55	−6.79	−2.78	4.01	−7.18	−4.46	−3.52

a Calculated using the DFT B3LYP/6-311++g(3df,2p) method.

**Fig. 4:**
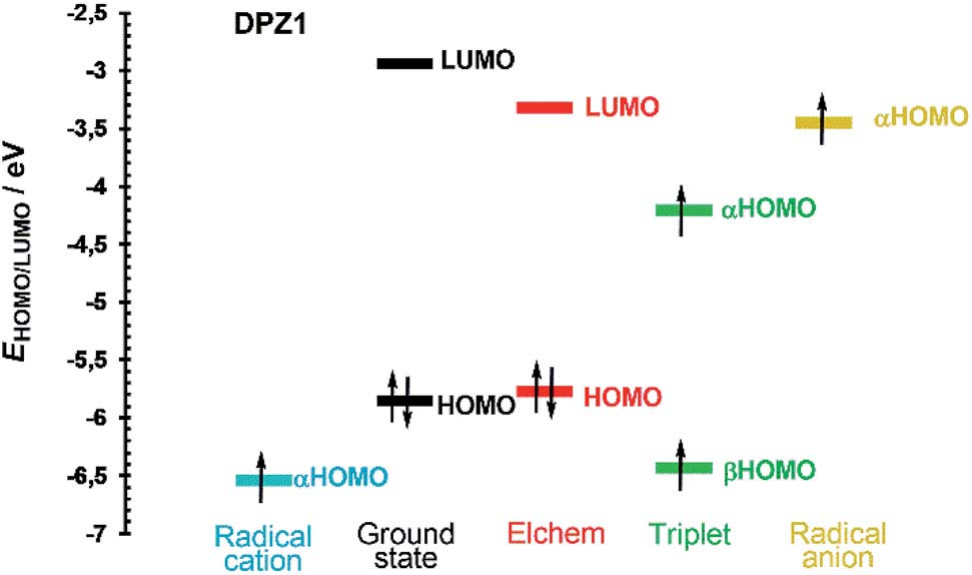
Energy level diagram of the frontier molecular orbitals and spin orbitals of DPZ 1.

Fig. 4 shows the frontier molecular orbital levels of the ground state (black) and spin orbitals of the species formed upon one electron transfer (blue, green and yellow). The comparison of the DFT and electrochemically obtained (red) HOMO/LUMO levels shows good agreement. Fig. 4 can be considered fundamental for interpreting the photoredox catalytic properties of DPZ 1. Compared to the non-ionized molecule, the radical cation possesses a lower highest occupied orbital (αHOMO) energy, which implies the more difficult removal of an additional electron from the positively charged species. On the contrary, the αHOMO level of the radical anion is higher, which facilitates the abstraction of the electron from the negatively charged species. Physical interpretation can be carried out based on the ionization energy/potential of the electron in the orbital, which is given by the negative value of the energy of the particular orbital (Koopman's theorem).^45^ Hence, the ionization potential of the radical cation is higher and radical anion lower than that of the non-ionized molecule. In the triplet, the ground state HOMO is split into two spin orbitals, α and β, with higher and lower energy, respectively. The unpaired electron from the αHOMO can be transferred to another molecule to generate a radical cation. On the contrary, the partially occupied βHOMO may easily accept an electron from a molecule and form a radical anion. Both electron transfers from/to the triplet are energetically very close to the levels of the radical cation/anion (Fig. 4). The aforementioned mechanism is most likely responsible for the visible light-induced photoredox activity of 1 and potentially also all the other DPZ derivatives (see also Fig. S21–S33 in the ESI†).

The CT-band of compounds 1, 2 and 12 is solely generated by the HOMO → LUMO excitation. For the remaining molecules, close orbitals such as HOMO−2/−1 and LUMO+1/+2 are also involved, with the typical configuration interaction of two or exceptionally three (for 4 and 11) electron transitions.

Multilinear regression implemented in the OPstat program (ref. 46) was applied to explore the dependence of the electrochemically obtained *E*_p(ox1)_ values on a maximum of three explanatory variables from the set of the orbital energies given in Table S2 (see the ESI).† The first oxidation potentials correlates tightly with *E*˙^+^(αHOMO) and *E*˙^+^(βHOMO) of the radical cation as well as with *E*^2+^ (HOMO) of the dication, excluding 4 and 11 as outliers (eqn (1)).

1 *E*_p(ox1)_ = −(1.219 ± 0.144) − (0.481 ± 0.034)*E*˙^+^(αHOMO) + (0.426 ± 0.049)*E*˙^+^(βHOMO) − (0.297 ± 0.029)*E*^2+^(HOMO),

*n* = 7, *s* = 1.697 10^−2^, *r* = 0.996, *F*(3,3) = 118.0.

As can be seen, the first oxidation potential does not correlate with the *E*^G^(HOMO) of the ground state, which implies that electron transfer from DPZ to the electrode is independent of the HOMO level. This further indicates that the common, widely applied and simplified correlations of the electrochemically and DFT-derived HOMO levels for push–pull molecules are incorrect. Eqn (1) also confirms the transfer of one/two electrons and formation of a radical cation/dication during the electrochemical oxidation of DPZs. The signs of the regression coefficients show that the first oxidation becomes difficult with more negative *E*˙^+^(αHOMO) and *E*^2+^(HOMO) values and more positive *E*˙^+^(βHOMO).

The first reduction peak potentials *E*_p(red1)_ were examined in the same way as *E*_p(ox1)_.

2 *E*_p(red1)_ = −(3.929 ± 0.312) − (0.612 ± 0.092)*E*˙^−^(αHOMO) − (0.270 ± 0.032)*E*^2−^(HOMO),

*n* = 13, *s* = 3.425 10^−2^, *r* = 0.972, *F*(2,10) = 84.14.

According to eqn (2), it was found that *E*_p(red1)_ depends on the *E*˙^−^(αHOMO) of the radical anion and *E*^2−^(HOMO) of the dianion. DPZ 7 with a limiting *E*_p(red1)_ was excluded as an outlier. The correlations are again very tight and independent of *E*^G^(LUMO). This implies that the electron transfer from the electrode to the molecule does not depend on the lowest unoccupied molecular orbital in the ground state, to which the electron is being transferred, but solely on the new orbitals generated during the electron transfer. Eqn (2) also reveals that both one- and two-electron processes are possible and the reduction is facilitated by the lowered energies *E*˙^−^(αHOMO) and *E*^2−^(HOMO).

From the aforementioned analysis, we can conclude that the electrochemical potentials can be quantum-chemically quantified very well. Especially considering that radical cation and anions are open shell systems and DFT calculations were carried out just for the most stable conformer.

### Visible light-induced photoredox activity

According to the first application of DPZ 1,^24^ the catalytic activity of all the synthesized PCs 1–14 was firstly examined in a benchmark cross-dehydrogenative coupling (CDC) reaction^26^ between *N*-phenyltetrahydroisoquinoline and nitromethane, as shown in Table 3.

**Table 3:** Benchmark photoredox CDC reaction catalysed by DPZ catalysts 1–14

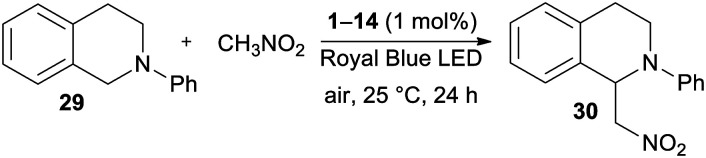			
Catalyst	Isolated yield [%]	Catalyst	Isolated yield [%]
1	96	8	88
2	86	9	93
3	90	11	83
4	85	12	95
5	75	13	85
6	93	14	89
7	88		

To compare the catalytic performance of all catalysts, the CDC reactions were carried out under standard conditions (1 mol% of the catalyst, Royal Blue LED source, 25 °C and 24 h reaction time). All reactions were repeated at least two times, and the average isolated yields ranged from 75% to 96%. Thus, all the PCs were capable, upon photoexcitation, to undergo SET and induce the one-electron oxidation of THIQ 29 to a radical cation. Although only limited excite state reduction potentials *E*_red_* are available (Table 1, Fig. S19 in the ESI†), the measured values are generally higher than the oxidation potential of the starting THIQ (0.79 V). As can be seen, the highest conversion and yields were achieved with original DPZ 1 and with new the DPZs 6, 9 and 12. On the other hand, DPZ 5 provided functionalized THIQ 30 in a diminished yield of75%. In contrast to DPZ 1 with a CT-band (*λ*^A^_max_ = 440 nm) almost perfectly centered to the emission band of the Royal Blue LED (∼430 nm), the absorption maximum of 5 is significantly blue-shifted to 279 nm with a diminished overlap. The absorption spectra of 6, 9 and 12 (*λ*^A^_max_ = 307, 343 and 351 nm) showed better overlap with the emission peak of the light source. DPZ 5 also possesses one of the most positive *E*^T^(βHOMO), which further hindered efficient SET from the substrate. Considering the structure of the most active catalysts, it seems that the presence of peripheral OMe/SMe groups (1, 12, and 6), thiophene moiety (9) or sulfidic linker (6 are 12) is the most crucial factor affecting their performance in the CDC reaction. This is consistent with the aforementioned structure–property relationships.

Secondly, the catalytic activity of DPZs 1–14 was further screened in a more challenging annulation reaction between *N*,*N*-dimethylaniline 31 and *N*-phenylmaleimide 32. This reaction was first reported by Murata *et al.*;^47^ however, its photoredox version was recently reinvestigated.^48^ Besides common PCs such as *N*-hydroxyphthalimide and Ru(bpy)_3_Cl_2_, we were also curious whether it could be catalysed by DPZs.

The optimization of the reaction conditions using DPZ 1 is summarized in Table 4. The starting aniline 31 and maleimide 32 were reacted in a 2 : 1 molar ratio. The solvent screening revealed acetone was the best solvent, and irradiation and the presence of PCs are also essential for the reaction. Carrying out the annulation with the optimized catalyst loading of 0.5 mol% and the reaction time of 2.2 h (as monitored by GC/MS) afforded the desired product 33 in 95% isolated yield. Further extension of the reaction time led to the undesired oxidation of unreacted 31 to *N*-methyl-*N*-phenylformamide.

**Table 4:** Optimization of the annulation reaction

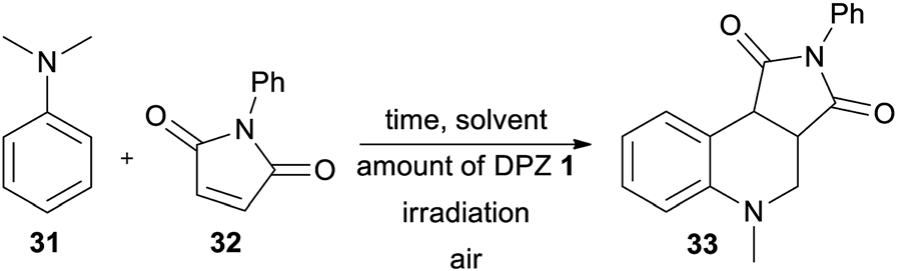				
Solvent	DPZ loading [mol%]	Time [h]	Irradiation with blue LED	Isolated yield [%]
AcCN	2	24	Yes	66
Acetone	2	24	Yes	80
DCM	2	24	Yes	59
1,4-Dioxane	2	24	Yes	55
Acetone	2	24	No	0
Acetone	0	24	Yes	0
Acetone	3	2.2	Yes	80
Acetone	1	2.2	Yes	83
Acetone	0.5	2.2	Yes	95

In contrast to the CDC reaction, the catalytic performance of DPZs 1–14 in the annulation reaction differed considerably. The best conversion and isolated yields of product 33 were achieved with 2,3-isomers 1 and 3 with OMe and SMe donors, respectively. Both catalysts showed very similar and the most bathochromically shifted absorption maxima (440 and 443 nm, respectively, Table 1), which most likely ensure the efficient absorption of light and facilitate one-electron oxidation of the starting aniline 31. The plausible mechanism further involves the formation of an α-aminoalkyl radical, its reaction with maleimide and final cyclization/rearomatization.^48*e*,*f*^ The calculated optical and electrochemical HOMO–LUMO gaps of the successful catalysts 1 and 3 are the lowest within the entire DPZ series (2.73/2.63 and 2.92/2.81 eV, respectively). Removal of the OMe or SMe peripheral groups, as in 2, resulted in a hypsochromic shift of the CT-band, enlarged HOMO–LUMO gaps and, on the contrary, deepened *E*^T^(βHOMO). However, the catalytic performance of 2 was worse than that of 1 and 3. It should also be noted that the excited state reduction potentials, *E*_red_*, of 1 and 3 are very similar and closest to the oxidation potential of *N*,*N*-dimethylaniline (Fig. S19 in the ESI†). The 2,6-isomeric DPZ derivatives proved much less efficient than the 2,3-isomers. A noticeable yield of 33 was only achieved with DPZs 9 and 11. When comparing the catalytic performance of the most active DPZs 1 and 3 with the formerly applied PCs (see above), they proved superior especially in terms of much shorter reaction time (generally 18–36 h) and low catalyst loading (generally 1–20 mol%) (Table 5).

**Table 5:** Visible light-induced annulation reaction catalysed by DPZ catalysts 1–14

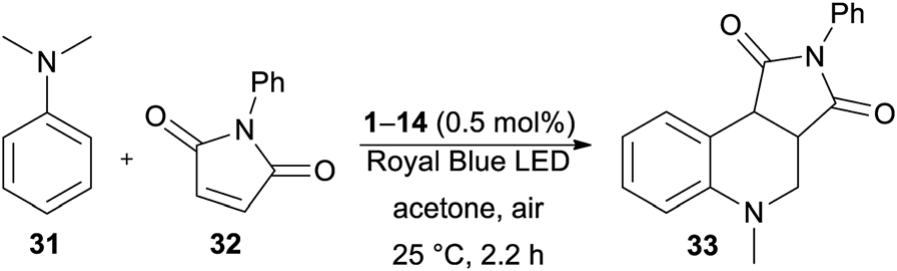			
Catalyst	Isolated yield [%]	Catalyst	Isolated yield [%]
1	95	8	10
2	73	9	36
3	94	11	32
4	7	12	13
5	14	13	11
6	7	14	7
7	14		

The recent reports^48^ focused mostly on the variation of the maleimide *N*-substituents (phenyl, substituted aryls, alkyls, *etc.*) and partially also on (4-)substituted *N*,*N*-dialkylaniline. Only Wu *et al.*^48*e*^ examined different acceptors such as maleic anhydride and acyclic olefins and found them unsuitable. Hence, we turned our attention towards expanding the original maleimide to the six-membered 1,2-dimethyl-1,2-dihydropyridazine-3,6-dione 34 (Table 6), which is easily accessible from maleic anhydride and dimethylhydrazine (see the ESI†). However, under the conditions optimized for the annulation of *N*-phenylmaleimide 32, no cyclization product was observed. Although acetonitrile also proved to be unsuitable solvent, the reaction in 1,4-dioxane afforded an inseparable mixture of the desired product 35 and the noncyclic intermediate. Upon further solvent optimization and testing various Lewis acids (ZnCl_2_, SmOTf, and LiPF_6_), we successfully isolated the target pyridazino[4,5-*c*]quinoline derivative 35 in 40% yield. Utilizing a dioxane : H_2_O (1 : 2) mixture and 0.1 eq. of LiPF_6_ allowed the reaction of α-aminoalkyl radical generated from 31 with 34 and subsequent cyclization to 35. An increase in the amount of Lewis acid, elevated temperature and increase in the amount of catalyst (0.5–10 mol%) did not significantly change the isolated yield (36–40%). The photoredox activity of DPZ 1 was further compared with common PCs such as Rose Bengal, Eosin Y, Ru(bpy)_3_Cl_2_ and acridinium perchlorate (Table 6). Although there was limited available data for the commercial catalyst, we can reasonably assume that the diminished catalytic activity of Ru(bpy)_3_Cl_2_ is due to the low oxidation capability Ru^II+^. On the other hand, the excited state reduction potential of acridinium perchlorate (*E*_0,0_ = 2.67 eV)^49^ is too far from the oxidation potential of the aniline 3. Eosin Y possesses *E*_0,0_ = 2.31 eV,^50^ which is close to that of DPZ, provides 35 in diminished 20% yield. However, Rose Bengal with similar redox properties was inactive in the formation of 35. Table 7 summarizes the catalytic activity of all the DPZ catalysts under the optimized conditions and 72 h reaction time. Similarly to previous observations, catalysts 1 and 3 proved to be the most efficient and afforded 35 in the highest yields. Moreover, these two catalysts directed the reaction solely towards 35 with diminished side reactions, such as oxidation and dimerization of the starting aniline 31. Using 1 and 3, the reaction could be completed within 10 days with the isolated yields of 93% and 90%, respectively. Quinoline polycyclic systems represent structural motifs often found in medicinal chemistry, drugs, alkaloids and DNA-binders. For instance, Catto *et al.* demonstrated the cytotoxic activity of pyridazino[4,3-*c*]quinoline derivatives against HeLa and MCF-7 human cancer cell lines.^51^ Pyridazino-quinoline derivatives have also been proven to be useful in the treatment of neurological disorders.^52^ Hence, we believe that the developed photoredox strategy will open a new synthetic pathway towards pyridazino-quinoline derivatives.

**Table 6:** Optimization of the annulation reaction with pyridazine-3,6-dione

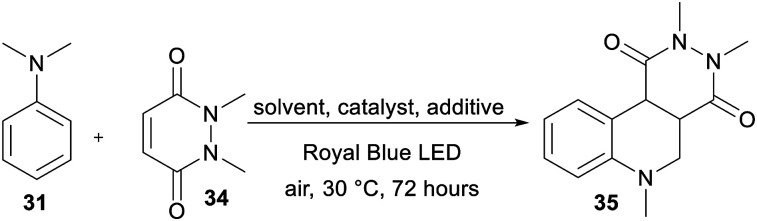				
Catalyst	Catalyst loading [mol%]	Solvent	Additive^a^	Yield [%]
DPZ 1	0.5	Acetone	—	—
DPZ 1	0.5	AcCN	—	—
DPZ 1	0.5	1,4-Dioxane	—	20^b^
DPZ 1	0.5	Dioxane : H_2_O	LiPF_6_	40
DPZ 1	0.5	Dioxane : H_2_O	ZnCl_2_	34
DPZ 1	0.5	Dioxane : H_2_O	SmOTf	35
DPZ 1	2	Dioxane : H_2_O	LiPF_6_	36
DPZ 1	5	Dioxane : H_2_O	LiPF_6_	38
DPZ 1	10	Dioxane : H_2_O	LiPF_6_	39
Rose Bengal^c^	0.5	Dioxane : H_2_O	LiPF_6_	—
Eosin Y	0.5	Dioxane : H_2_O	LiPF_6_	20
Ru(bpy)_3_Cl_2_	0.5	Dioxane : H_2_O	LiPF_6_	Traces
Acridinium perchlorate^d^	0.5	Dioxane : H_2_O	LiPF_6_	Traces

a 0.1 eq.

b According to GC/MS, accompanied by noncyclic intermediate.

c Irradiated by the green LED (530 nm).

d 9-Mesityl-10-methylacridinium perchlorate.

**Table 7:** Visible light-induced annulation reaction catalysed by DPZ catalysts 1–14

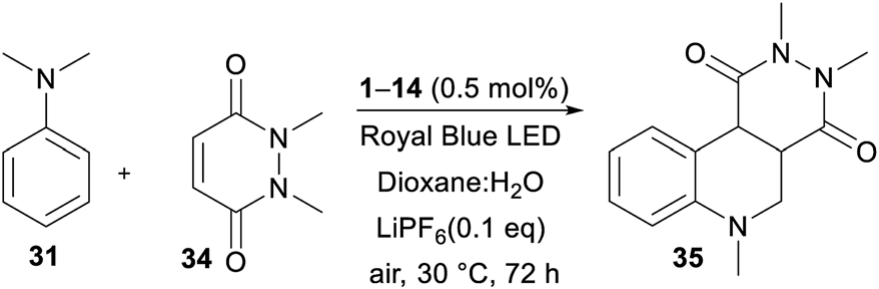			
Catalyst	Isolated yield [%]	Catalyst	Isolated yield [%]
1	40/93^a^	8	20
2	5	9	25
3	37/90^a^	11	28
4	5	12	35
5	5	13	10
6	5	14	5
7	5		

a Reaction times of 72/240 h.

## Conclusion

A series of dicyanopyrazine-derived X-shaped push–pull molecules was designed and prepared. The target D–π–A molecules possess a systematically altered structure with varying positions of the CN groups and different donors and linkers. The X-ray analysis showed significant changes in the spatial arrangements of the target dicyanopyrazines depending on the substituents. The structural changes were also reflected in the optical and electrochemical properties of all the PCs. Their optical and electrochemical gaps could be tuned within the wide range of 1.64 eV and 0.67 V, respectively, and the excited state reduction/oxidation potentials were found to be up to 2.47/−1.18 V. The DFT calculations, especially the frontier molecular orbital and spin orbital diagrams, allowed interpretation of the photoredox fundamental properties of all the PCs. A close correlation between the electrochemically measured and DFT-calculated data with novel physico-chemical interpretation was provided. Finally, the photoredox activities of the target PCs were examined in a benchmark cross-dehydrogenative coupling and novel annulation reactions. We demonstrated the superior photoredox activity of the DPZ catalysts and first VL-induced formation of pyridazino[4,5-*c*]quinoline derivatives.

## Experimental

All reagents and solvents were reagent grade and were purchased from Penta, Aldrich, and TCI and used as received. The starting 4,5-dichloropyrazine-3,6-dicarbonitrile 15, thiophen-2-yl boronic acid 19, 2-thiophenethiol 22, and sodium thiomethoxide 24 were commercially available. All cross-coupling reactions were carried out in flame-dried flasks under an argon atmosphere. Thin layer chromatography (TLC) was conducted on aluminium sheets coated with silica gel 60 F254 with visualization by a UV lamp (254 or 360 nm). Column chromatography was carried out with silica gel 60 (particle size 0.040–0.063 mm, 230–400 mesh) and commercially available solvents. ^1^H and ^13^C NMR spectra were recorded on a Bruker AVANCE II/III 400/500 spectrometer (400/500 MHz or 100/125 MHz, respectively). Chemical shifts are reported in ppm relative to the signal of Me_4_Si (0.00 ppm). The residual solvent signal was used as an internal reference (CDCl_3_ 7.25 and 77.23 ppm). Apparent resonance multiplicities are described as s (singlet), d (doublet), dd (doublet of doublet), and m (multiplet). ^1^H NMR signals of thiophene are denoted as Th. The coupling constants, *J*, are reported in Hertz (Hz). High-resolution MALDI mass spectroscopy data were collected on an LTQ Orbitrap XL. Absorption spectra were measured on a UV/Vis PerkinElmer Lambda 35 spectrophotometer at room temperature. Fluorescence spectra were measured on a PTI Quantamaster 40 steady state spectrofluorimeter, and dilute solutions with absorption bands were used for these fluorescence measurements. The fluorescence kinetics were measured on Horiba Jobin Yvon Fluorolog TCSPC spectrofluorimeter with excitation using an IBH NanoLED 405 nm of about 250 ps FWHM pulse duration. The fluorescence lifetimes were obtained using the iterative reconvolution procedure.

### General procedure for the Suzuki–Miyaura cross-coupling

In a Schlenk flask, 5,6-dichloropyrazine-2,3-dicarbonitrile 15 (199 mg, 1.0 mmol) or 3,5-dichloropyrazine-2,6-dicarbonitrile 16 (199 mg, 1.0 mmol) and a suitable amount of boronic acid or boronic acid pinacol ester (2.2 eq.) were dissolved in a mixture of THF/H_2_O (50 mL, 4 : 1). Argon was bubbled through the solution for 10 min, whereupon Pd_2_(dba)_3_ (46 mg, 0.05 mmol, 5%), SPhos (20.5 mg, 0.05 mmol, 5%), and Cs_2_CO_3_ (684 mg, 2.1 mmol) were added and the resulting reaction mixture was stirred at 65 °C for the indicated time. The reaction mixture was diluted with water (40 mL) and extracted with CH_2_Cl_2_ (3 × 40 mL). The combined organic extracts were dried over anhydrous Na_2_SO_4_, the solvents were evaporated *in vacuo* and the crude product was purified by column chromatography.

### General procedure for the synthesis of compounds 5, 6 and 12

In a Schlenk flask, 2-methoxythiophene (400 mg, 3.5 mmol) or 2-methylthiothiophene (458 mg, 3.5 mmol) was dissolved in dry THF (20 mL) at −78 °C. *n*BuLi (1.62 mL, 2.5 M sol. in hexane, 4 mmol) was added dropwise and the mixture was stirred 1 hour at −78 °C. 1,2-Bis(dimethylamino)ethane (41 mg, 0.35 mmol) and sulphur (112 mg, 3.5 mmol) were added and stirring was continued at −45 °C for 1 h. Thereafter, 5,6-dichloropyrazine-2,3-dicarbonitrile 15 (358 mg, 1.8 mmol) or 3,5-dichloropyrazine-2,6-dicarbonitrile 16 (358 mg, 1.8 mmol) was added and the resulting solution was stirred overnight. The reaction mixture was diluted with water (20 mL) and extracted with CH_2_Cl_2_ (3 × 20 mL). The combined organic extracts were dried over anhydrous Na_2_SO_4_, the solvents were evaporated *in vacuo* and the crude product was purified by flash chromatography.

### General procedure for the synthesis of compounds 4 and 11

Thiophene-2-thiol 22 (100 mg, 0.5 mmol) and 5,6-dichloropyrazine-2,3-dicarbonitrile 15 (199 mg, 1 mmol) or 3,5-dichloropyrazine-2,6-dicarbonitrile 16 (199 mg, 1 mmol) were dissolved in acetone (10 mL) and pyridine (0.1 mL, 1.5 mmol) was added. The resulting mixture was stirred for the indicated time. The reaction mixture was diluted with water (10 mL) and extracted with CH_2_Cl_2_ (3 × 10 mL). The combined organic extracts were dried over anhydrous Na_2_SO_4_, the solvents were evaporated *in vacuo* and the crude product was purified by flash chromatography.

### General procedure for the synthesis of compounds 7 and 13

A solution of triethylamine (0.15 mL, 1.1 mmol) in methanol (1 mL) was added dropwise to a solution of 5,6-dichloropyrazine-2,3-dicarbonitrile 15 (100 mg, 0.5 mmol) or 3,5-dichloropyrazine-2,6-dicarbonitrile 16 (100 mg, 0.5 mmol) in methanol 23 (2.5 mL). The resulting mixture was stirred for the indicated time. The reaction was diluted with water and the crude product was extracted with Et_2_O (3 × 5 mL). The combined organic extracts were dried over anhydrous Na_2_SO_4_, the solvents were evaporated *in vacuo* and the crude product was purified by flash chromatography.

### General procedure for the synthesis of compounds 8 and 14

A solution of 5,6-dichloropyrazine-2,3-dicarbonitrile 15 (100 mg, 0.5 mmol) or 3,5-dichloropyrazine-2,6-dicarbonitrile 16 (100 mg, 0.5 mmol) was dissolved in acetone (1.5 mL) at 0 °C, and sodium thiomethoxide 24 (0.35 g, 21% aq. sol.) was added dropwise. The resulting reaction mixture was stirred at 0 °C for 1 h. Then the solvent was evaporated *in vacuo* and the crude product was washed with water, followed by crystallization from ethanol.

#### 5,6-Bis(5-methoxythiophen-2-yl)pyrazine-2,3-dicarbonitrile (1)^24^

The title compound was prepared from 15 (199 mg, 1.0 mmol) and pinacol ester 17 (528 mg, 2.2 mmol) following the general method for the Suzuki–Miyaura reaction (reaction time = 6 h). Compound 1 was an orange solid (304 mg, 86%); mp = 178 °C. *R*_f_ = 0.20 (SiO_2_, CH_2_Cl_2_ : Hex = 2 : 1). Spectral data according to the literature.^24^

#### 5,6-Di(thiophen-2-yl)pyrazine-2,3-dicarbonitrile (2)^24^

The title compound was prepared from 15 (199 mg, 1.0 mmol) and 2-thienylboronic acid 19 (281 mg, 2.2 mmol) following the general method for the Suzuki–Miyaura reaction (reaction time = 3 h). Compound 2 was a yellow solid (72 mg, 28%); mp = 178 °C. *R*_f_ = 0.20 (SiO_2_, CH_2_Cl_2_ : Hex = 2 : 1). Spectral data according to the literature.^24^

#### 5,6-Bis(5-(methylthio)thiophen-2-yl)pyrazine-2,3-dicarbonitrile (3)

The title compound was prepared from 15 (199 mg, 1.0 mmol) and 4,4,5,5-tetramethyl-2-(5-(methylthio)thiophen-2-yl)-1,3,2-dioxaborolane 18 (563 mg, 2.2 mmol) following the general method for the Suzuki–Miyaura reaction (reaction time = 1 h). Compound 3 was a red solid (97 mg, 25%); mp = 168–171 °C. *R*_f_ = 0.25 (SiO_2_, CH_2_Cl_2_ : Hex = 1 : 1). ^1^H-NMR (500 MHz, CDCl_3_): *δ*(^1^H) = 7.62 (d, *J* = 4 Hz, 2H, Th), 6.86 (d, *J* = 4 Hz, 2H, Th), 2.63 (s, 6H, OCH_3_) ppm. ^13^C-NMR (125 MHz, CDCl_3_): *δ*(^13^C) = 149.87, 146.31, 137.33, 131.94, 127.43, 113.32, 19.88 ppm. HR-FT-MALDI-MS (DHB): *m*/*z* calcd. for C_16_H_10_N_4_S_4_^+^ ([M^+^]), 385.97828; found: 385.97878.

#### 5,6-Bis(thiophen-2-ylthio)pyrazine-2,3-dicarbonitrile (4)

The title compound was prepared from 15 (199 mg, 1.0 mmol) and thiophene-2-thiol 22 (100 mg, 0.5 mmol) following the general method (reaction time = 5 h). Compound 4 was a yellow solid (215 mg, 60%); mp > 220 °C (decomp.). *R*_f_ = 0.40 (SiO_2_, CH_2_Cl_2_ : Hex = 1 : 1). ^1^H-NMR (400 MHz, CDCl_3_): *δ*(^1^H) = 7.44 (dd, *J* = 1.2 and 5.6 Hz, 2H, Th), 7.35 (dd, *J* = 1.2 and 3.6 Hz, 2H, Th), 7.19 (dd, *J* = 3.6 and 9.2 Hz, 2H, Th) ppm. ^13^C-NMR (125 MHz, CDCl_3_): *δ*(^13^C) = 159.01, 138.45, 134.50, 128.68, 120.31, 113.30 ppm. HR-FT-MALDI-MS (DHB): *m*/*z* calcd. for C_14_H_7_N_4_S_4_^+^ ([M + H^+^]), 358.95481; found: 358.95496.

#### 5,6-Bis((5-methoxythiophen-2yl)thio)pyrazine-2,3-dicarbonitrile (5)

The title compound was prepared from 15 (358 mg, 1.8 mmol) and 2-methoxythiophene (400 mg, 3.5 mmol) following the general method (reaction time = 14 h). Compound 5 was a yellow solid (300 mg, 40%); mp = 209–212 °C. *R*_f_ = 0.15 (SiO_2_, CH_2_Cl_2_ : Hex = 1 : 1). ^1^H-NMR (500 MHz, CDCl_3_): *δ*(^1^H) = 7.02 (d, *J* = 4 Hz, 2H, Th), 6.26 (d, *J* = 4 Hz, 2H, Th), 3.96 (s, 6H, OCH_3_) ppm. ^13^C-NMR (125 MHz, CDCl_3_): *δ*(^13^C) = 173.00, 159.96, 138.31, 128.56, 116.65, 105.90, 105.05, 60.56 ppm. HR-FT-MALDI-MS (DHB): *m*/*z* calcd. for C_16_H_10_O_2_N_4_S_4_^+^ ([M^+^]), 419.96811; found: 419.96794.

#### 5,6-Bis((5-(methylthio)thiophen-2yl)thio)pyrazine-2,3-dicarbonitrile (6)

The title compound was prepared from 15 (358 mg, 1.8 mmol) and thiophene-2-thiol 22 (458 mg, 3.5 mmol) following the general method (reaction time = 14 h). Compound 6 was a yellow solid (267 mg, 33%); mp = 145–148 °C. *R*_f_ = 0.85 (SiO_2_, CH_2_Cl_2_ : Hex = 2 : 1). ^1^H-NMR (500 MHz, CDCl_3_): *δ*(^1^H) = 7.19 (d, *J* = 4 Hz, 2H, Th), 7.06 (d, *J* = 4 Hz, 2H, Th), 2.58 (s, 6H, SCH_3_) ppm. ^13^C-NMR (125 MHz, CDCl_3_): *δ*(^13^C) = 158.89, 148.12, 138.97, 129.82, 128.48, 119.75, 113.34, 21.04 ppm. HR-FT-MALDI-MS (DCTB): *m*/*z* calcd. for C_16_H_10_N_4_S_6_^+^ ([M^+^]), 449.92242; found: 449.92258.

#### 5,6-Dimethoxypyrazine-2,3-dicarbonitrile (7)

The title compound was prepared from 15 (100 mg, 0.5 mmol) and methanol 23 (2.5 mL) following the general method (reaction time = 4 h). Compound 6 was a pale yellow solid (80 mg, 85%); mp = 162–165 °C. *R*_f_ = 0.80 (SiO_2_, Et_2_O : Hex = 2 : 1). ^1^H-NMR (400 MHz, CDCl_3_): *δ*(^1^H) = 4.12 (s, 6H, OCH_3_) ppm. ^13^C-NMR (125 MHz, CDCl_3_): *δ*(^13^C) = 152.12, 123.02, 113.30, 56.13 ppm. Spectral data according to the literature.^36^

#### 5,6-Bis(methylthio)pyrazine-2,3-dicarbonitrile (8)

The title compound was prepared from 5,6-dichloropyrazine-2,3-dicarbonitrile 15 (100 mg, 0.5 mmol) and sodium thiomethoxide 24 (0.35 g, 21% aq. sol.) following the general method (reaction time = 1 h). Compound 8 was a pale green solid (52 mg, 47%); mp = 157–160 °C. *R*_f_ = 0.9 (SiO_2_, CH_2_Cl_2_).·^1^H-NMR (400 MHz, CDCl_3_): *δ*(^1^H) = 2.66 (s, 6H, SCH_3_) ppm. ^13^C-NMR (125 MHz, CDCl_3_): *δ*(^13^C) = 160.77, 126.50, 113.93, 14.10 ppm. HR-FT-MALDI-MS (none): *m*/*z* calcd. for C_8_H_6_N_4_S_2_^+^ ([M^+^]), 222.00284; found: 222.00287.

#### 3,5-Di(thiophen-2-yl)pyrazine-2,6-dicarbonitrile (9)

The title compound was prepared from 16 (199 mg, 1.0 mmol) and 2-thienylboronic acid 19 (281 mg, 2.2 mmol) following the general method for the Suzuki–Miyaura reaction (reaction time = 14 h). Compound 8 was a yellow solid (132 mg, 45%); mp = 237–238 °C. *R*_f_ = 0.63 (SiO_2_, CH_2_Cl_2_ : Hex = 1 : 1). ^1^H-NMR (400 MHz, CDCl_3_): *δ*(^1^H) = 8.48 (dd, *J* = 1 and 4 Hz, 2H, Th), 7.88 (dd, *J* = 0.4 and 4.8 Hz, 2H, Th), 7.29 (dd, *J* = 4 and 4.8 Hz, 2H, Th) ppm. ^13^C-NMR (125 MHz, CDCl_3_): *δ*(^13^C) = 150.45, 138.10, 135.51, 132.50, 130.10, 120.36, 115.72 ppm. HR-FT-MALDI-MS (DHB): *m*/*z* calcd. for C_14_H_7_N_4_S_2_^+^ ([M + H^+^]), 295.01066; found: 295.01054.

#### 3,5-Bis(5-methoxythiophen-2-yl)pyrazine-2,6-dicarbonitrile (10)

The title compound was prepared from 16 (199 mg, 1.0 mmol) and pinacol ester 17 (528 mg, 2.2 mmol) following the general method for the Suzuki–Miyaura reaction (reaction time = 5 h). Compound 10 was an orange solid (106 mg, 30%) which gradually decomposed during storage; mp = 183–186 °C. *R*_f_ = 0.39 (SiO_2_, CH_2_Cl_2_ : Hex = 1 : 1). ^1^H-NMR (400 MHz, CDCl_3_): *δ*(^1^H) = 8.23 (d, *J* = 4.8 Hz, 2H, Th), 6.37 (d, *J* = 4.8 Hz, 2H, Th), 4.03 (s, 6H, OCH_3_) ppm. ^13^C-NMR (125 MHz, CDCl_3_): *δ*(^13^C) = 175.74, 150.01, 133.17, 124.29, 117.28, 116.36, 107.96, 60.80 ppm. HR-FT-MALDI-MS (DHB): *m*/*z* calcd. for C_16_H_12_N_4_O_2_S_2_^+^ ([M + 2H^+^]), 356.03962; found: 356.04000.

#### 3,5-Bis(thiophen-2-ylthio)pyrazine-2,6-dicarbonitrile (11)

The title compound was prepared from thiophene-2-thiol 22 (100 mg, 0.5 mmol) and 16 (199 mg, 1 mmol) following the general method (reaction time = 14 h). Compound 11 was a yellow solid (86 mg, 24%); mp = 183–186 °C. *R*_f_ = 0.49 (SiO_2_, CH_2_Cl_2_ : Hex = 1 : 1). ^1^H-NMR (500 MHz, CDCl_3_): *δ*(^1^H) = 7.49 (dd, *J* = 1 and 5.5 Hz, 2H, Th), 7.06 (dd, *J* = 1 and 3.5 Hz, 2H, Th), 6.98 (dd, *J* = 4 and 5.5 Hz, 2H, Th) ppm. ^13^C-NMR (125 MHz, CDCl_3_): *δ*(^13^C) = 164.17, 138.20, 134.54, 128.34, 121.89, 120.06, 113.11 ppm. HR-FT-MALDI-MS (DHB): *m*/*z* calcd. for C_14_H_6_N_4_S_4_^+^ ([M + H^+^]), 358.95481; found: 358.95486.

#### 3,5-Bis((5-methoxythiophen-2-yl)thio)pyrazine-2,6-dicarbonitrile (12)

The title compound was prepared from 2-methoxythiophene (400 mg, 3.5 mmol) and 16 (358 mg, 1.8 mmol) following the general method (reaction time = 24 h). Compound 12 was a yellow solid (308 mg, 41%); mp = 168–172 °C. *R*_f_ = 0.36 (SiO_2_, CH_2_Cl_2_ : Hex = 1 : 1). ^1^H-NMR (400 MHz, CDCl_3_): *δ*(^1^H) = 6.84 (d, *J* = 4 Hz, 2H, Th), 6.10 (d, *J* = 4 Hz, 2H, Th), 3.92 (s, 6H, OCH_3_) ppm. ^13^C-NMR (125 MHz, CDCl_3_): *δ*(^13^C) = 172.71, 165.39, 138.13, 121.63, 113.23, 105.78, 104.88, 60.35 ppm. HR-FT-MALDI-MS (DCTB): *m*/*z* calcd. for C_16_H_8_N_4_O_2_S_4_^+^ ([M − H^+^]),416.96028; found: 416.96046.

#### 3,5-Dimethoxypyrazine-2,6-dicarbonitrile (13)

The title compound was prepared from 16 (100 mg, 0.5 mmol) and methanol 23 (2.5 mL) following the general method (reaction time = 12 h). Compound 12 was a yellow solid (68 mg, 72%); mp = 142–146 °C. *R*_f_ = 0.75 (SiO_2_, Et_2_O : Hex = 2 : 1). ^1^H-NMR (400 MHz, CDCl_3_): *δ*(^1^H) = 4.18 (s, 6H, OCH_3_) ppm. ^13^C-NMR (125 MHz, CDCl_3_): *δ*(^13^C) = 162.32, 112.78, 110.90, 56.03 ppm. Spectral data according to the literature.^40^

#### 3,5-Bis(methylthio)pyrazine-2,6-dicarbonitrile (14)

The title compound was prepared 5,6-dichloropyrazine-2,3-dicarbonitrile 16 (100 mg, 0.5 mmol) and sodium thiomethoxide 24 (0.35 g, 21% aq. sol.) following the general method (reaction time = 1 h). Compound 14 was a pale yellow solid (44 mg, 40%); mp = 219–220 °C. *R*_f_ = 0.86 (SiO_2_, CH_2_Cl_2_).·^1^H-NMR (500 MHz, CDCl_3_): *δ*(^1^H) = 2.70 (s, 6H, SCH_3_) ppm. ^13^C-NMR (125 MHz, CDCl_3_): *δ*(^13^C) = 164.42, 121.64, 113.51, 13.55 ppm. HR-FT-MALDI-MS (none): *m*/*z* calcd. for C_8_H_6_N_4_S_2_^+^ ([M^+^]), 222.00284; found: 222.00302.

### General method for the CDC reaction

In a 10 mL snap vial equipped with a magnetic stirring bar, THIQ 29 (31 mg, 0.15 mmol) was dissolved in nitromethane (1.5 mL) and then photocatalyst 1–14 (1.5 μmol) was added. The reaction mixture was stirred under irradiation with a Royal Blue LED at 25 °C from a distance of 5 cm. The temperature was maintained using a Peltier cooler. After 24 h, the solvent was removed *in vacuo* and the residue was purified by flash chromatography (SiO_2_; petroleum ether (PE) : ethyl acetate (EA) 10 : 1) to obtain pure product 30.

### General method for the annulation reaction

#### Method A

In a 5 mL snap vial equipped with a magnetic stirring bar, *N*,*N*-dimethylaniline 31 (63 μL, 0.5 mmol) and *N*-phenylmaleimide 32 (43 mg, 0.25 mmol) were dissolved in acetone (3 mL) and then photocatalyst 1–14 (0.5 mol%) was added. The reaction mixture was stirred for 2 h and 20 min at 25 °C (Peltier cooler) under irradiation by a Royal Blue LED from a distance of 5 cm. The solvent was removed *in vacuo* and the crude product was purified by column chromatography (Al_2_O_3_, PE : EA 3 : 1) to obtain pure product 33.

#### Method B

In a 5 mL snap vial equipped with a magnetic stirring bar, *N*,*N*-dimethylaniline 31 (63 μL, 0.5 mmol) and 1,2-dimethyl-1,2-dihydropyridazine-3,6-dione 34 (35 mg, 0.25 mmol) were dissolved in a mixture of 1,4-dioxane : H_2_O (1 mL : 2 mL), and thereafter photocatalyst 1–14 (0.5 mol%) and LiPF_6_ (4 mg, 0.025 mmol) were added. The reaction mixture was stirred 72 h at 30 °C (Peltier cooler) under irradiation by a Royal Blue LED from a distance of 5 cm. The solvent was removed *in vacuo* and the crude product was purified by flash chromatography (SiO_2_, EA) to obtain pure product 35.

## Conflicts of interest

There are no conflicts to declare.

## Supplementary Material

RA-009-C9RA04731J-s001

RA-009-C9RA04731J-s002
