# Supplementary material for: Structural elaboration of dicyanopyrazine: towards push–pull molecules with tailored photoredox activity
Source: RSC Adv. 2019 Jul 31;9(41):23797–809. doi: 10.1039/c9ra04731j (PMC9069489; doi:10.1039/c9ra04731j)
Supplement: RA-009-C9RA04731J-s001 [file RA-009-C9RA04731J-s001.pdf]

This journal is © The Royal Society of Chemistry 2019

Table of contents

1. Synthesis and characterizations of intermediates 2
 1.1. 2-Methoxythiophene 2
 1.2. 2-(Methylthio)thiophene 2
 1.3. 5-Methoxythiophen-2-yl boronic acid pinacol ester (17) 2
 1.4. 5-Methylthiothiophen-2-yl boronic acid pinacol ester (18) 3
 1.5. Intermediate 25 3
 1.6. Intermediate 26 4
 1.7. Intermediate 27 4
 1.8. 3-Amino-5-chloropyrazine-2,6-dicarbonitrile (28) 4
 1.9. 3,5-Dichloropyrazine-2,6-dicarbonitrile (16) 4
 1.10. 1,2-Dimethyl-1,2-dihydropyridazin-3,6-dione (34) 5
2. Catalysis 5
3. X-Ray analysis 6
4. Electronic absorption spectra 9
5. Electrochemistry 11
6. DFT calculations 19
 6.1. Representative localizations of frontier molecular orbitals 21
 6.2. Frontier molecular orbitals and spinorbitals of DPZs 1–14 22
 6.3. Calculated spectra of DPZ derivatives 1–14 29
7. ^{1}H and ^{13}C NMR spectra 32
8. References 47

1. Synthesis and characterizations of intermediates

1.1. 2-Methoxythiophene

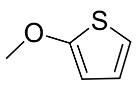

Sodium (14 g; 0.689 mol) was dissolved in CH_3OH (100 ml) under reflux. 2-Bromothiophene (11.9 ml; 0.122 mol) was added dropwise followed by CuBr (3.5 g; 0.024 mol). The resulting mixture was heated in oil bath at 135 °C overnight. The reaction mixture was allowed to reach 25 °C, a solution of NaCN (6 g; 0.122 mol) in water (100 ml) was added and the reaction was stirred for 15 minutes. The reaction mixture was extracted with DCM (3 × 100 ml), the combined organic extracts were dried (Na_2SO_4), and the solvents were evaporated *in vacuo*. The crude product was purified by vacuum distillation (b.p. 70–80 °C; 67 Torr). 2-Methoxythiophene is a colorless liquid (6.65 g, 48 %); n_D^{25} = 1.5250. R_f = 0.48 (SiO_2; hexane). ^1H-NMR (400 MHz, 25 °C, $CDCl_3$): δ = 6.76 (dd, J = 3.6 a 5.6 Hz, 1H, Th); 6.57 (dd, J = 1.2 a 5.6 Hz, 1H, Th); 6.25 (dd, J = 1.2 a 3.6 Hz, 1H, Th); 3.92 (s, 3H, OCH_3) ppm. Spectral data according to the literature.[1]

1.2. 2-(Methylthio)thiophene

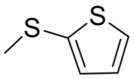

To a solution of NaOH (0.24 g; 6 mmol) in ethanol (5 ml), thiophene-2-thiol (0.5 ml; 5 mmol) was added. The resulting reaction mixture was stirred at room temperature for 2 hours. Subsequently, iodomethane (0.34 ml; 5.5 mmol) was added and the solution was stirred for 12 hours. The solvent was evaporated *in vacuo*, followed by addition of water (5 ml), extraction with Et_2O (3 × 5 ml). The combined organic extracts were dried (Na_2SO_4) and the solvents were evaporated *in vacuo*. The crude product was purified by column chromatography (SiO_2, pentane). 2-(methylthio)thiophene is a colorless liquid (0.2 g, 67 %); n_D^{25} = 1.5949. R_f = 0.85 (SiO_2;pentane). ^1H-NMR (400 MHz, 25 °C, $CDCl_3$): δ = 7.29 (dd, J = 1.2 and 5.3 Hz, 1H, Th); 7.08 (dd, J = 1.2 and 3.6 Hz, 1H, Th); 6.93 (dd, J = 3.6 a 5.3 Hz, 1H, Th); 2.49 (s, 3H, SCH_3); 2.15 (s, 12H, CH_3) ppm. Spectral data according to the literature.[2]

1.3. 5-Methoxythiophen-2-yl boronic acid pinacol ester (17)

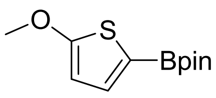

A solution of 2-methoxythiophene (500 mg; 4.38 mmol) in dry THF (20 ml) was treated with *n*BuLi (3.1 ml; 5.04 mmol, 1.6 M sol. in hexane) at –78 °C under argon for 1 hour. 2-Isopropoxy-4,4,5,5-tetramethyl-1,3,2-dioxaborolane (1.03 ml, 5.04 mmol; *i*PrOBpin) was added, and the reaction mixture was allowed to reach 25 °C and stirred for 1 hour. NH_4Cl (10 ml, sat. aq. sol.) was added and the reaction mixture was extracted with Et_2O (3 × 10

ml). The combined organic extracts were dried (Na_2SO_4) and the solvents were evaporated *in vacuo*. Additional purification of the product is possible by a flash chromatography (SiO_2, DCM:Hex 1:1). The title product is a yellowish solid (936 mg, 89 %); mp 36– 42 °C. R_f = 0.38 (SiO_2, DCM:Hex 1:1). ^{1}H-NMR (400 MHz, 25 °C, $CDCl_3$): δ = 7.31 (d, 1H, J = 4 Hz, Th); 6.28 (d, 1H, J = 4 Hz, Th); 3.89 (s, 3H, OCH_3); 2.15 (s, 12H, CH_3) ppm. Spectral data according to the literature.[3]

1.4. 5-Methylthiothiophen-2-yl boronic acid pinacol ester (18)

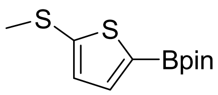

To the solution of 2-(methylthio)thiophene (85 mg; 0.65 mmol) in dry THF (10 ml), *n*BuLi (0.3 ml; 0.8 mmol, 2.5 M sol. In hexane) at –78 °C under argon atmosphere for 1 hour. Reaction mixture was stirred additional 1 hour at 25 °C, then 2-isopropoxy-4,4,5,5-tetramethyl-1,3,2-dioxaborolane (0.3 g, 1.4 mmol; *i*PrOBpin) was added at –60 °C and the reaction mixture was allowed to reach 25 °C and stirred for 1 hour. NH_4Cl (10 ml, sat. aq. sol.) was added and the reaction mixture was extracted with Et_2O (3 × 10 ml). The combined organic extracts were dried (Na_2SO_4) and the solvents were evaporated *in vacuo*. The crude product was purified by flash chromatography (SiO_2, DCM:Hex 1:1). The title product is a violet liquid (115 mg, 69 %); n_D^{25} = 1.5459. R_f = 0.56 (SiO_2; DCM:Hex = 1:2). ^{1}H-NMR (400 MHz, 25 °C, $CDCl_3$): δ = 7.46 (d, J = 3.6 Hz, 1H, Th); 7.02 (d, J = 3.6 Hz, 1H, Th); 2.52 (s, 3H, SCH_3); 1.32 (s, 12H, CH_3) ppm. Spectral data according to the literature.[4]

1.5. Intermediate 25

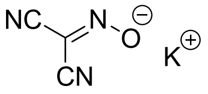

To the solution of malononitrile (6.4 g; 0.098 mol) in acetic acid (14 ml) and water (36 ml), $NaNO_2$ (10.1 g; 0.146 mol) was added dropwise at 0–5 °C. The resulting reaction mixture was stirred for 45 minutes at 3 °C. Thereafter, 25 % aq. sol. HCl (70 ml) was added and resulting solution was extracted with Et_2O (3 × 50 ml). The combined organic extracts were dried (Na_2SO_4) and the solvents were evaporated *in vacuo*. The crude brown oil was slowly dropping into the ice cold solution of KOH (2.85 g) in MeOH (70 ml). Solution reacted 20 minutes at 3°C, after that Et_2O (300 ml) was added and crystals grew immediately. Product **25** was filtered to obtain 6.56 g (50 %) of bright yellow crystals; mp = 207–210 °C. ^{13}C-NMR (125 MHz, 25 °C, d_4-MeOH): δ = 117.64; 112.07; 109.01 ppm. Spectral data according to the literature.[5]

1.6. Intermediate 26

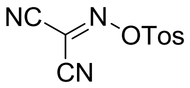

Precursor **25** (3.17 g; 0.024 mol) was dissolved in acetonitrile (30 ml), and *p*-toluenesulfonyl chloride was added in a few portions. Stirring of resulting solution continued for 2 hours at 30 °C, with subsequent addition of water (130 ml). After 30 minutes without stirring, white crystals were filtered off. Product **26** is a white solid (4.5 g, 76 %); mp = 109–112 °C. ^{1}H-NMR (400 MHz, 25 °C, $CDCl_3$): δ = 7.90 (d, J = 8 Hz, 2H, Ph); 7.45 (d, J = 8 Hz, 2H, Ph); 2.50 (s, 3H, CH_3) ppm. Spectral data according to the literature.[6]

1.7. Intermediate 27

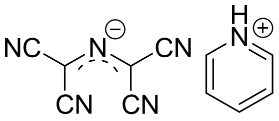

Malononitrile (0.7 g; 10 mmol) was dissolved in pyridine (2 ml; 25 mmol) and Et_2O (2.5 ml) at 0 °C. Into this solution, compound **26** (2.5 g, 10 mmol) in Et_2O (75 ml) was added dropwise. Resulting reaction mixture stand for 1 day, after that precipitate was filtered off. The crude product was purified by stirring with Et_2O and hexane for 15 minutes and subsequent filtration. The title product **27** is a white solid (1.9 g, 89 %); mp = 150–153 °C. ^{1}H-NMR (400 MHz, 25 °C, d_6-DMSO): δ = 8.93 (m, 2H); 8.54 (m, 1H); 8.03 (m, 2H) ppm.

1.8. 3-Amino-5-chloropyrazine-2,6-dicarbonitrile (28)

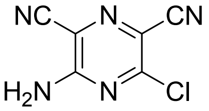

Precursor **27** (100 mg; 0.4 mmol) was dissolved in 18 % aq. sol. HCl (2 ml) and heated to 80 °C for 15 minutes. Formed precipitate was filtered off. The title product **28** is brown solid (33 mg, 45 %); mp = 234–236 °C (decomp.). ^{1}H-NMR (400 MHz, 25 °C, d_6-aceton): δ = 8.10 (s, 2H, NH_2) ppm. Spectral data according to the literature.[7]

1.9. 3,5-Dichloropyrazine-2,6-dicarbonitrile (16)

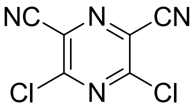

In a vacuum dried Schlenk flask under argon precursor **28** (100 mg; 0.6 mmol), $CuCl_2$ (113 mg; 0.8 mmol) was dissolved in dry acetonitrile (5 ml). Then *iso*amylnitrite (98 mg; 0.8 mmol) was added and reaction mixture was stirred 10 hours at 65 °C. 20% aq. sol. HCl (50 ml) was added and resulting solution was extracted with DCM (3 × 30 ml). The combined organic extracts were dried (Na_2SO_4) and the solvents were evaporated *in vacuo*. The crude product was purified by flash chromatography (SiO_2, DCM). Pyrazine **16** is white solid (56 mg, 50 %); mp = 170–172 °C. ^{13}C-NMR (125 MHz, 25 °C, d_6-aceton): δ = 153.69; 130.13; 113.81 ppm. Spectral data according to the literature.[8]

1.10. 1,2-Dimethyl-1,2-dihydropyridazin-3,6-dione (34)

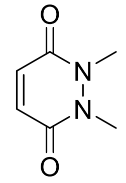

Maleic anhydride (4.45 g; 0.05 mol) and *N,N*-dimethylhydrazine (4.95 g; 0.037 mol) were dissolved in hot water (50 ml). Resulting reaction mixture was heated to reflux for 3 hours. After cooling to room temperature, the mixture was neutralized to pH 8 with K_2CO_3. Resulting reaction mixture was extracted with DCM (5 × 50 ml), combined organic extracts were dried (Na_2SO_4) and the solvents were evaporated *in vacuo*.[9] Pyridazine **34** is grey solid (1.7 g; 33 %); mp = 136–138 °C. ^{1}H-NMR (400 MHz, 25 °C, $CDCl_3$): δ = 6.86 (s, 2H); 3.59 (s, 6H, CH_3) ppm. ^{13}C-NMR (400 MHz, 25 °C, $CDCl_3$): δ = 156.83; 134.42; 32.82 ppm. HR-FT-MALDI-MS (DCTB): *m/z* calcd. for $C_6H_9N_2O_2^+$ ([M+H] $^+$), 141.06585; found 141.06583.

2. Catalysis

2.1. Product 30

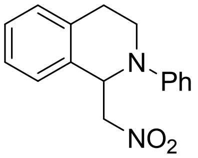

Yellow oil; ^{1}H-NMR (400 MHz, $CDCl_3$): δ = 7.33–7.29 (m, 2H); 7.28–7.26 (m, 1H); 7.24–7.21 (m, 2H); 7.16 (d, *J* = 7.0 Hz, 1H); 7.02 (d, *J* = 8.1 Hz, 2H); 6.88 (t, *J* = 7.3 Hz, 1H); 5.58 (t, *J* = 7.2 Hz, 1H), 4.90 (dd, *J* = 11.8, 7.9 Hz, 1H); 4.59 (dd, *J* = 11.8, 6.6 Hz, 1H); 3.73–3.61 (m, 2H); 3.16–3.08 (m, 1H); 2.82 (dt, *J* = 16.3, 4.9 Hz, 1H) ppm. ^{13}C-NMR (100 MHz, $CDCl_3$): δ = 148.4; 135.2; 132.8; 129.5; 129.2; 128.1; 127.0; 126.6; 119.4; 115.0; 78.7; 58.2; 42.0; 26.4 ppm; HR-FT-MALDI-MS (DCTB): *m/z* calcd. for 269.1283 ([M+H]$^+$), $C_{16}H_{17}N_2O_2$; found 269.1290. Spectral data according to the literature.[10]

2.2. Product 33

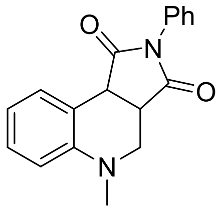

White solid; ^{1}H-NMR (500MHz, $CDCl_3$): δ = 7.54 (d, *J* = 7.5 Hz, 1H); 7.43 (m, 2H), 7.37 (td, *J* = 7.5, 15 Hz, 1H), 7.26 (m, 3H), 6.92 (t, *J* = 15 Hz, 1H), 6.76 (d, *J* = 8 Hz, 1H), 4.17 (d, *J* = 9,6 Hz, 1H), 3.62 (dd, *J* = 2.7, 11.5 Hz, 1H), 3.57–3.53 (m, 1H), 3.13 (dd, *J* = 4.4, 11.5 Hz, 1H), 2.85 (s, 3H); ^{13}C-NMR (125 MHz, $CDCl_3$): δ = 177.94; 175.98; 148.69; 132.12; 130.51; 129.20; 128.88; 128.72; 126.54; 119.86; 118.72; 50.83; 43.76; 42.30; 39.64; HR-FT-MALDI-MS (DCTB): *m/z* calcd. for $C_{18}H_{16}N_2O_2^+$ ([M]$^+$), 292.12063; found 292.11997. Spectral data according to the literature.[11]

2.3. Product 35

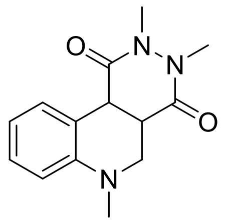

White solid; ^{1}H-NMR (400 MHz, 25 °C, $CDCl_3$): δ = 7.13 (m, 1H); 6.99 (d, J = 7,6 Hz, 1H); 6.56 (m, 2H); 3.90 (d, J = 8 Hz, 1H); 3.69 (dd, J = 4 a 12 Hz, 1H); 3.47 (dd, J = 4 a 12 Hz,1H); 3.28 (s, 3H); 3.17 (q, 1H); 2.95 (s, 3H); 2.94 (s, 3H) ppm. ^{13}C-NMR (100 MHz, 25 °C, $CDCl_3$): δ = 129.39; 126.67; 116.19; 111.13; 76.44; 71.33; 48.43; 43.20; 38.61; 37.40; 33.16; 32.92; 29.89; 18.75 ppm. HR-FT-MALDI-MS (none): *m/z* calcd. for $C_{14}H_{17}N_3O_2^+$ ($[M]^+$), 259.13153; found 259.13140.

3. X-Ray analysis

Full-sets of diffraction data for **7**, **12** and **27** were collected at 150(2)K with a Bruker D8-Venture diffractometer equipped with Mo (Mo/K_α radiation; λ = 0.71073 Å) microfocus X-ray (IμS) sources, Photon CMOS detector and Oxford Cryosystems cooling device was used for data collection.

The frames were integrated with the Bruker SAINT software package using a narrow-frame algorithm. Data were corrected for absorption effects using the Multi-Scan method (SADABS). Obtained data were treated by XT-version 2014/5 and SHELXL-2014/7 software implemented in APEX3 v2016.5-0 (Bruker AXS) system.[12]

Hydrogen atoms were mostly localized on a difference Fourier map, however to ensure uniformity of treatment of crystal, all hydrogen were recalculated into idealized positions (riding model) and assigned temperature factors $H_{iso}(H)$ = 1.2 U_{eq} (pivot atom) or of 1.5U_{eq} (methyl). Hydrogen atoms in methyl moiety and in aromatic rings were placed with C-H distances of 0.96 and 0.93Å.

$R_{int} = \sum|F_o^2 - F_{o,mean}^2|/\sum F_o^2$, $S = [\sum(w(F_o^2 - F_c^2)^2)/(N_{diffrs} - N_{params})]^{½}$ for all data, $R(F) = \sum||F_o| - |F_c||/\sum|F_o|$ for observed data, $wR(F^2) = [\sum(w(F_o^2 - F_c^2)^2)/(\sum w(F_o^2)^2)]^{½}$ for all data.

Crystallographic data for structural analysis have been deposited with the Cambridge Crystallographic Data Centre, CCDC no. 1897538, 1897539 and 1897540 for **7**, **12** and **27**, respectively. Copies of this information may be obtained free of charge from The Director, CCDC, 12 Union Road, Cambridge CB2 1EY, UK (fax: +44-1223-336033; e-mail: deposit@ccdc.cam.ac.uk or www: http://www.ccdc.cam.ac.uk).

Relevant crystallographic data and structural refinement parameters for **7**: $C_8H_6N_4O_2$, M = 190.17 g/mol, orthorhombic, *Pnma*, *a* = 9.3323(7), *b* = 14.8768(11), *c* = 6.3126(5) Å, β = 90°, Z = 4, V = 876.41(12) $Å^3$, D_c = 1.441 g.cm^{-3}, μ = 0.109 mm^{-1}, T_{min}/T_{max} = 0.6553/0.7456; -12≤ h≤ 11, -19≤ k≤ 18, -8≤ l≤ 8; 7842 reflections measured (θ_{max} = 27.51°), 1043 independent (R_{int} = 0.0557), 826 with $I > 2\sigma(I)$, 65 parameters, S = 1.073, *R1*(obs. data) = 0.0431, *wR2*(all data) = 0.1016; max., min. residual electron density = 0.310, -0.235 e Å^{-3}.

Crystallographic data and structural refinement parameters for **12**: $C_{10}H_{12}N_2O_2S_2$, M = 256.34 g/mol, monoclinic, *C2/c*, *a* = 8.762(3), *b* = 12.848(5), *c* = 16.367(7) Å, β = 101.625(18)°, Z = 8, V = 1804.5(19) $Å^3$, D_c = 1.536 g.cm^{-3}, μ = 0.544 mm^{-1}, T_{min}/T_{max} = 0.3675 /0.7456; -11 ≤ h ≤ 11, -16 ≤ k ≤ 16, -21 ≤ l ≤ 21; 17327 reflections measured (θ_{max} = 27.58°), 2081 independent (R_{int} = 0.1061), 1744 with $I > 2\sigma(I)$, 120 parameters, S = 1.073, *R1*(obs. data) = 0.0468, *wR2*(all data) = 0.1211; max., min. residual electron density = 0.355, -0.593 e Å^{-3}.

Crystallographic data and structural refinement parameters for **27**: $C_{11}H_6N_6$, M = 222.22 g/mol, monoclinic, $P2_1/m$, *a* = 3.7497(5), *b* = 14.1534(17), *c* = 10.3386(13) Å, β = 97.855(4)°, Z = 2, V = 543.53(12) $Å^3$, D_c = 1.358 g.cm^{-3}, μ = 0.091 mm^{-1}, T_{min}/T_{max} = 0.6744/ 0.7456; -4 ≤ h ≤ 4, -18 ≤ k ≤ 18, -13 ≤ l ≤ 13; 6571 reflections measured (θ_{max} = 27.58°), 1287 independent (R_{int} = 0.0304), 1009 with $I > 2\sigma(I)$, 85 parameters, S = 1.197, R_1(obs. data) = 0.0644, wR_2(all data) = 0.1462; max., min. residual electron density = 0.201, -0.435 e Å^{-3}.

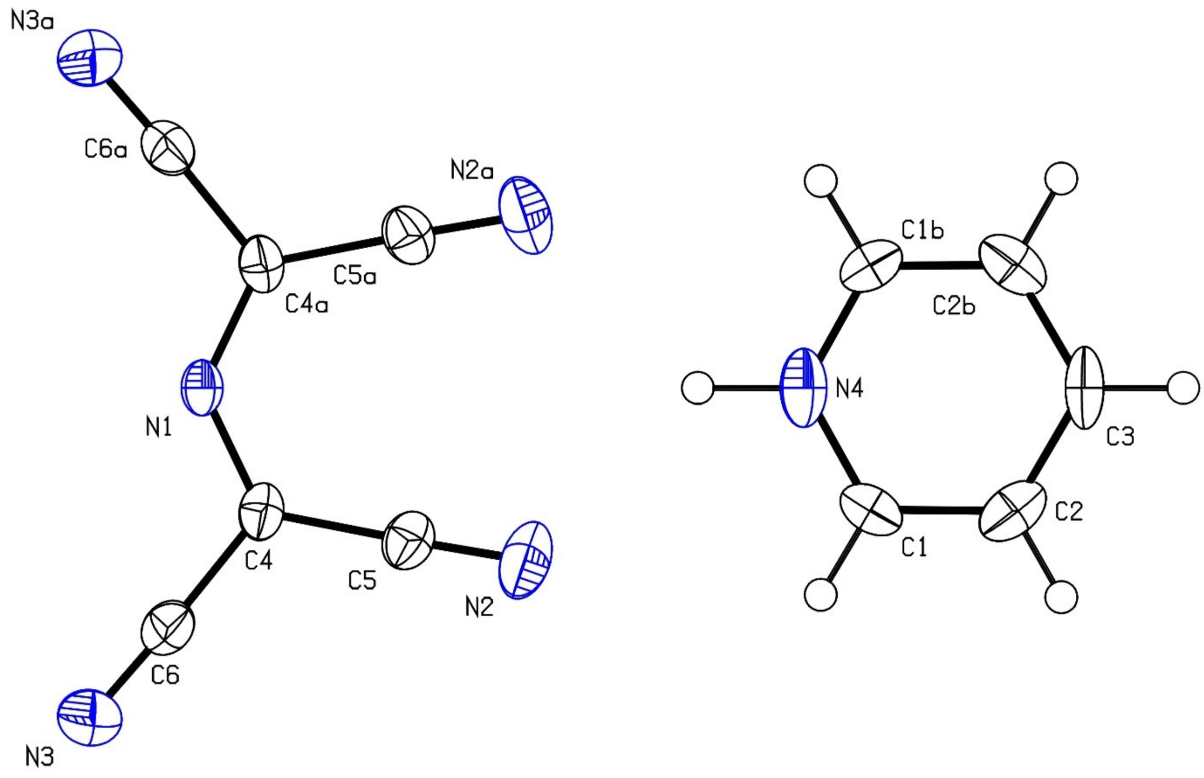

Figure S1. ORTEP representation of intermediate **27** (CCDC 1897540).

4. Electronic absorption spectra

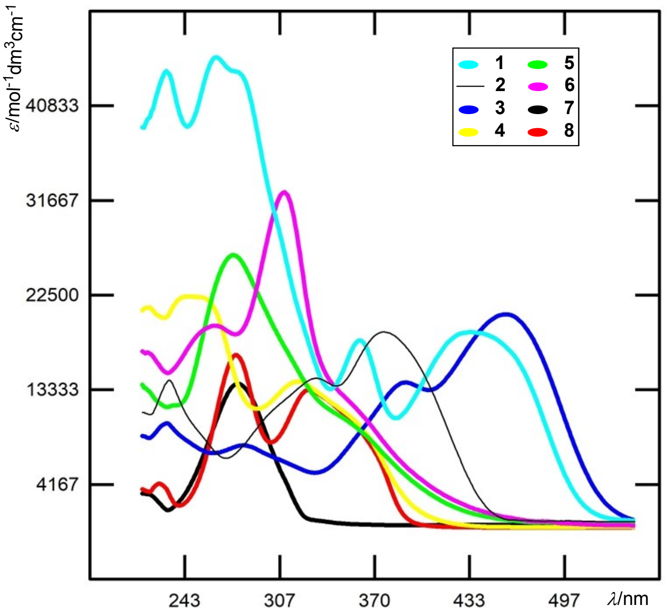

Figure S2. UV-Vis absorption spectra of catalysts **1–8** (DCM, 2 × 10^{-5} M).

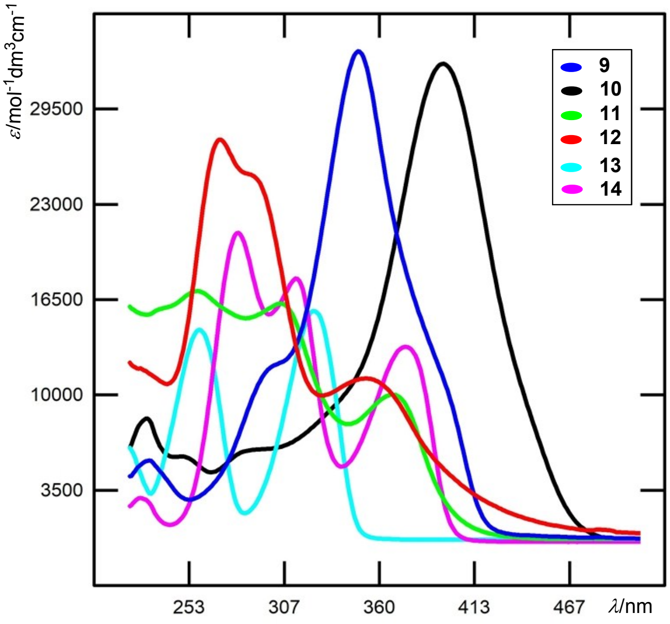

Figure S3. UV-Vis absorption spectra of catalysts **9–14** (DCM, 2 × 10^{-5} M).

Table S1. Longest-wavelength absorption/fluorescence maxima of catalysts **1**–**14** in various solvents.

**Comp.**	Dichloromethane			Acetonitrile		1,4-Dioxane	
	λ_{max}^{A} [nm]	ε [$M^{-1}cm^{-1}$]	λ_{max}^{F} [nm]	λ_{max}^{A} [nm]	λ_{max}^{F} [nm]	λ_{max}^{A} [nm]	λ_{max}^{F} [nm]
**1**	456	19 000	573	440	571	435	537
**2**	389	19 000	485	379	488	380	473
**3**	450	20 700	552	443	543	442	554
**4**	318	14 200	-	315	-	323	-
**5**	360	26 600	-	279	-	280	-
**6**	309	33 000	-	307	-	308	-
**7**	278	14 200	350	278	349	278	353
**8**	325	13 500	409	323	-	322	-
**9**	348	34 000	435	343	462	343	432
**10**	395	32 800	520	389	562	385	516
**11**	368	10 100	-	363	-	366	-
**12**	355	11 100	-	351	-	355	-
**13**	324	16 400	365	322	363	324	367
**14**	375	13 400	412	371	417	372	412

5. Electrochemistry

The electrochemical characteristics of target DPZs **1**–**14** were investigated in acetonitrile containing 0.1 M Bu_4NPBF_4 in a three electrode cell by cyclic voltammetry (CV). Voltammetric measurements were performed by using an integrated potentiostat system ER466 (eDAQ, Denistone East, Australia) operated with EChem Electrochemistry software. The working electrode was glassy carbon disk (1 mm in diameter). As the reference and auxiliary electrodes were used leakless Ag/AgCl electrode (SSCE) containing filling electrolyte (3.4 M KCl) and titanium rod with a thick coating of platinum, respectively. All peak potentials are given *vs*. SSCE. The reversible reduction of residual oxygen (≈ –0.9 V) as well as irreversible oxidation of impurity in commercial Bu_4NPBF_4 (≈ 0.75 V), respectively, can be seen in some CV diagrams.

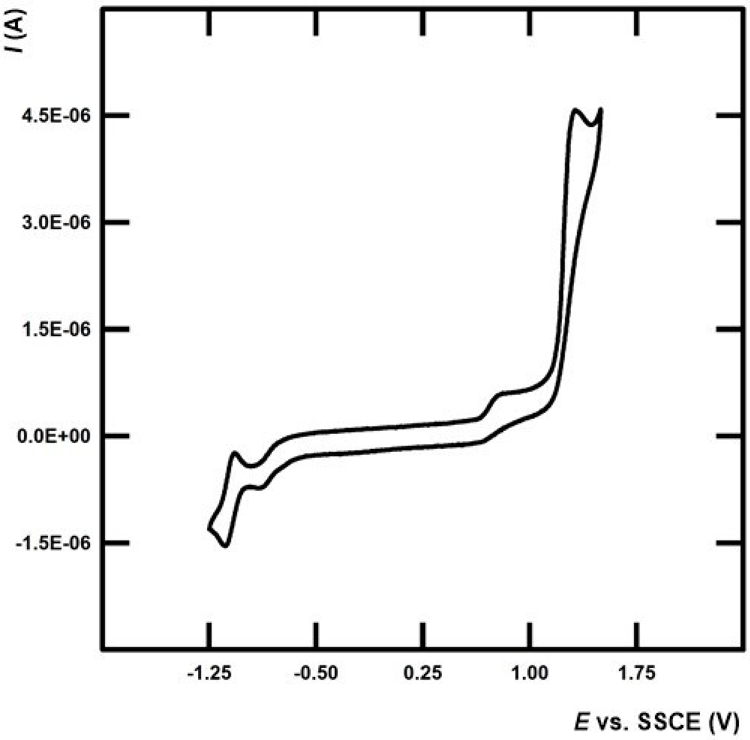

Figure S4. CV diagram of compound **1** at GC electrode in acetonitrile containing 0.1 M Bu_4NBF_4; v=100mV/s.

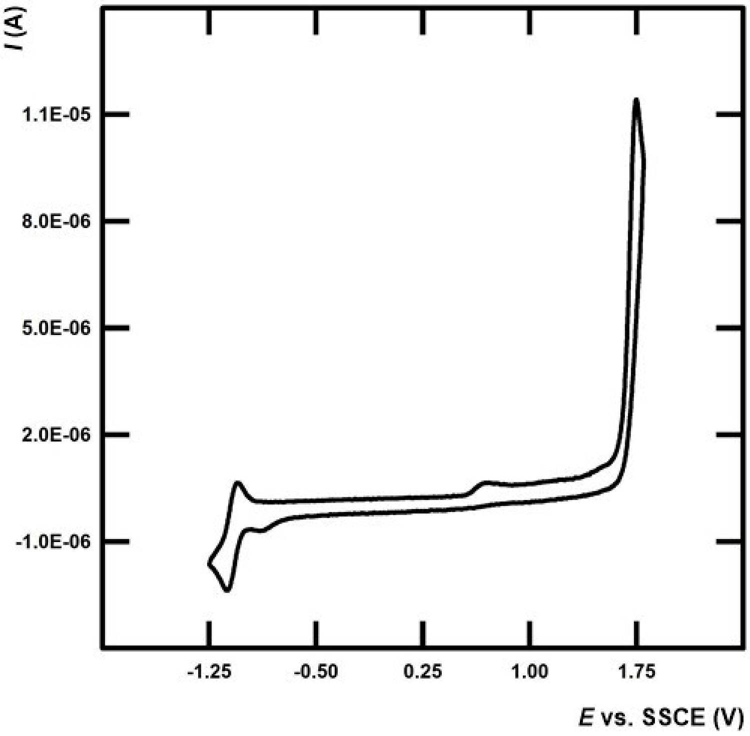

Figure S5. CV diagram of compound **2** at GC electrode in acetonitrile containing 0.1 M Bu_4NBF_4; v=100mV/s.

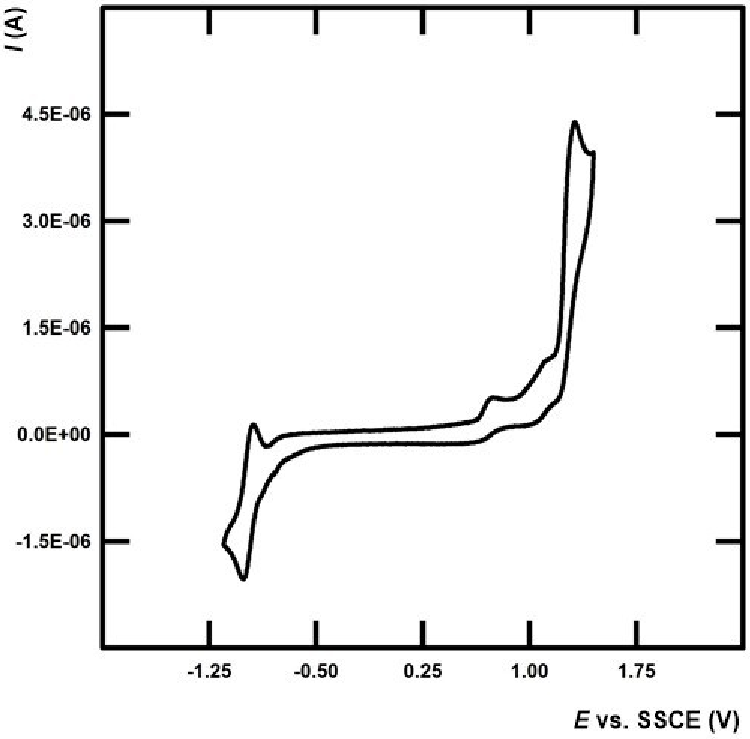

Figure S6. CV diagram of compound **3** at GC electrode in acetonitrile containing 0.1 M Bu_4NBF_4; v=100mV/s.

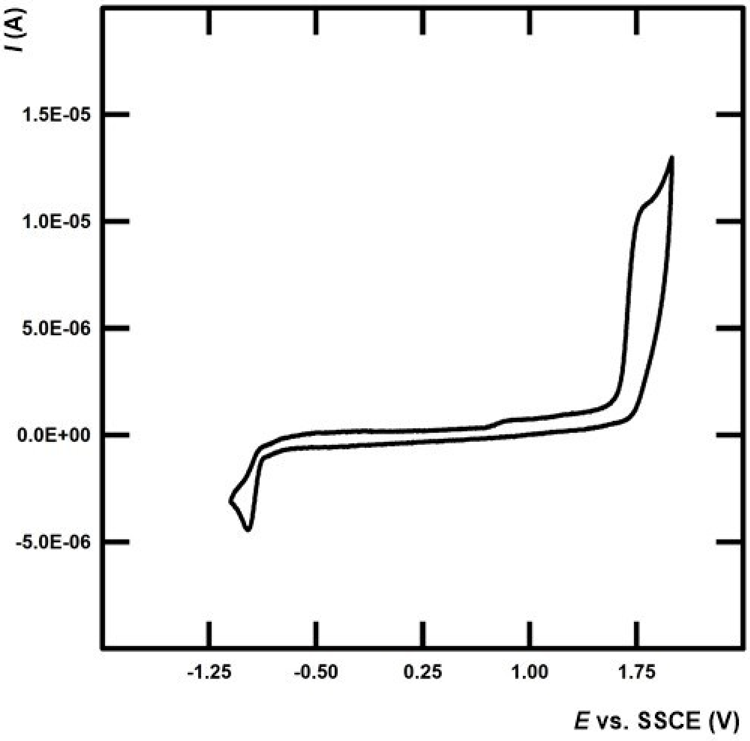

Figure S7. CV diagram of compound **4** at GC electrode in acetonitrile containing 0.1 M Bu_4NBF_4; v=100mV/s.

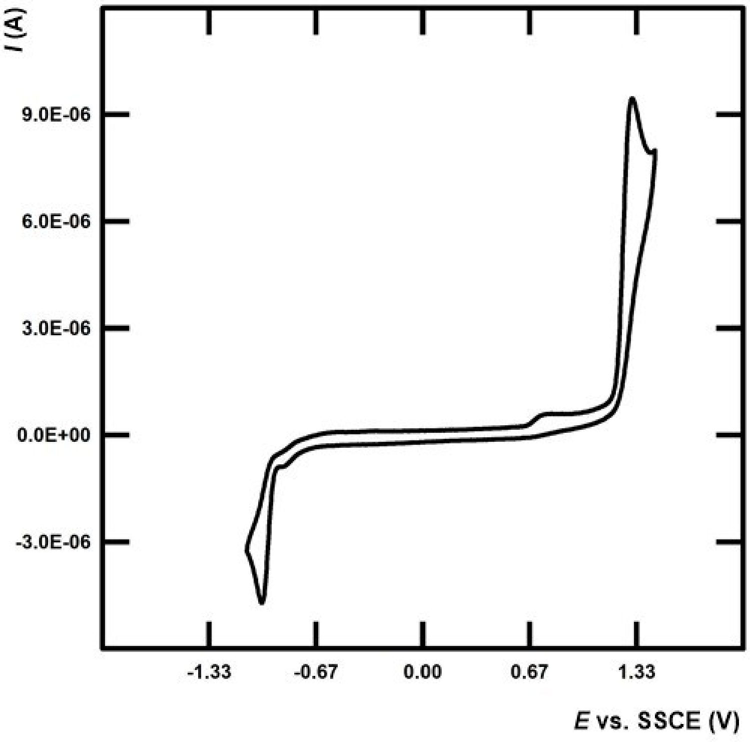

Figure S8. CV diagram of compound **5** at GC electrode in acetonitrile containing 0.1 M Bu_4NBF_4; v=100mV/s.

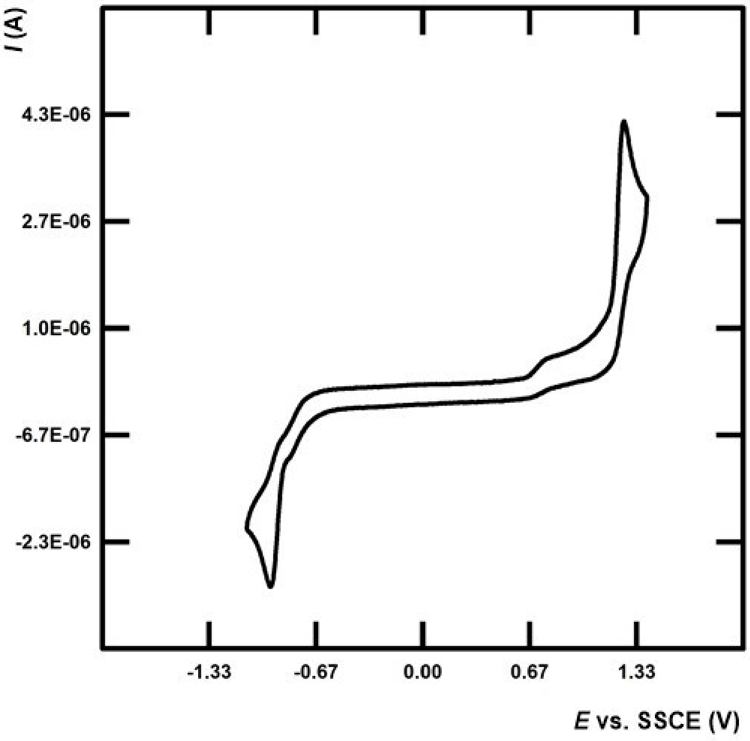

Figure S9. CV diagram of compound **6** at GC electrode in acetonitrile containing 0.1 M Bu_4NBF_4; v=100mV/s.

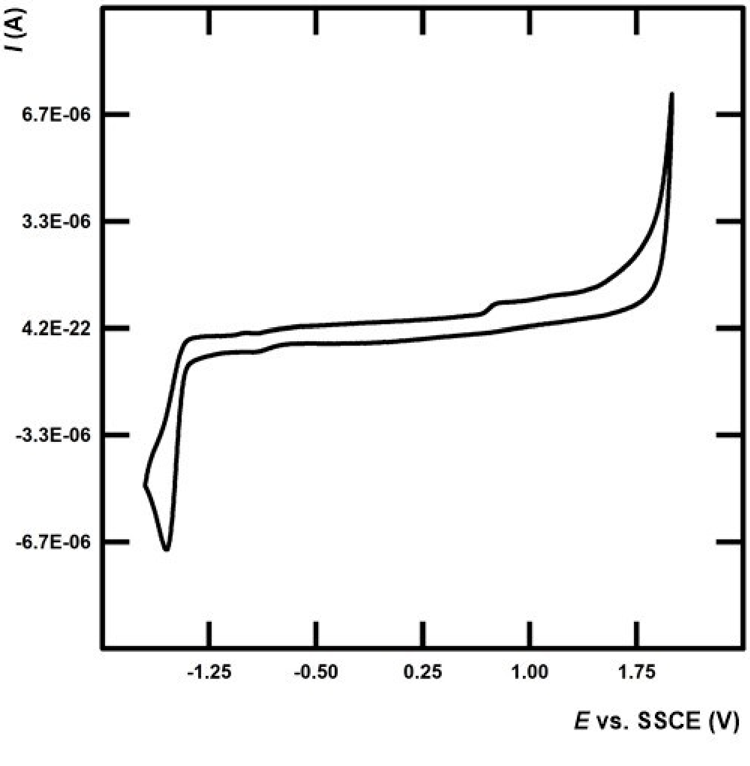

Figure S10. CV diagram of compound **7** at GC electrode in acetonitrile containing 0.1 M Bu_4NBF_4; v=100mV/s.

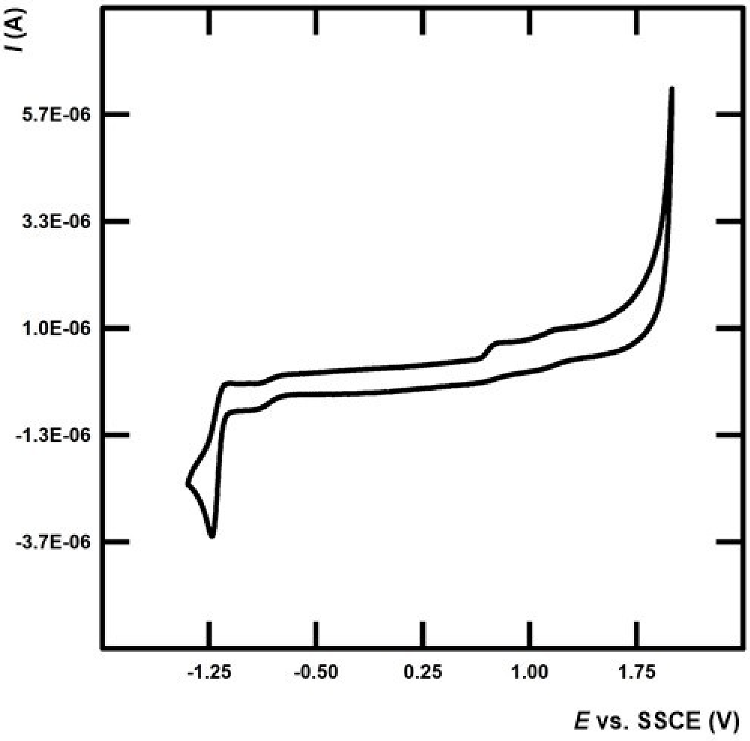

Figure S11. CV diagram of compound **8** at GC electrode in acetonitrile containing 0.1 M Bu_4NBF_4; v=100mV/s.

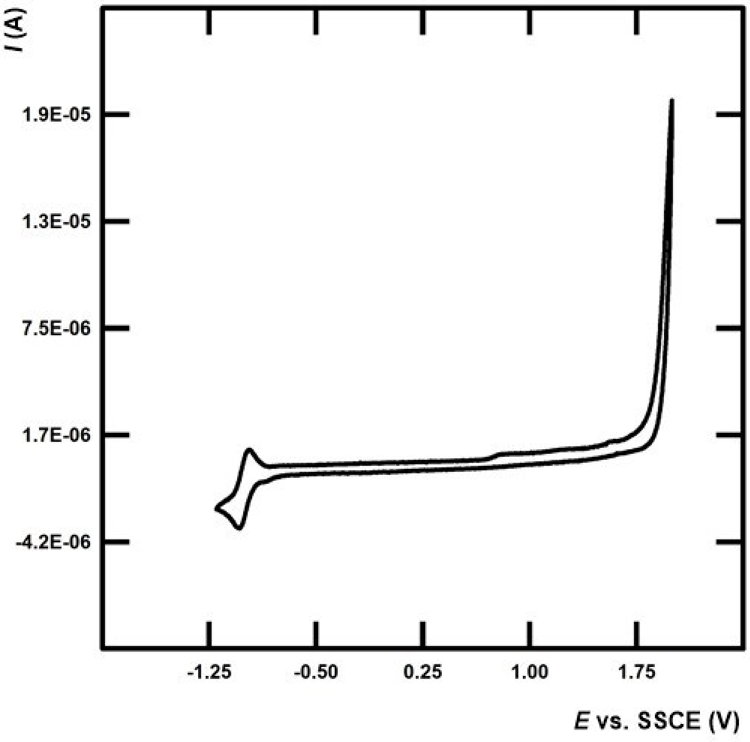

Figure S12. CV diagram of compound **9** at GC electrode in acetonitrile containing 0.1 M Bu_4NBF_4; v=100mV/s.

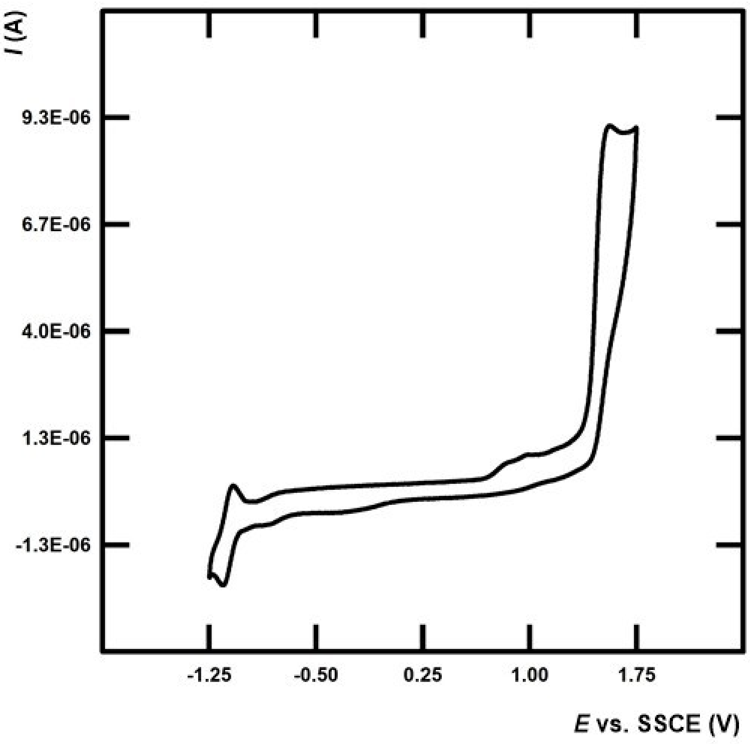

Figure S13. CV diagram of compound **10** at GC electrode in acetonitrile containing 0.1 M Bu_4NBF_4; v=100mV/s.

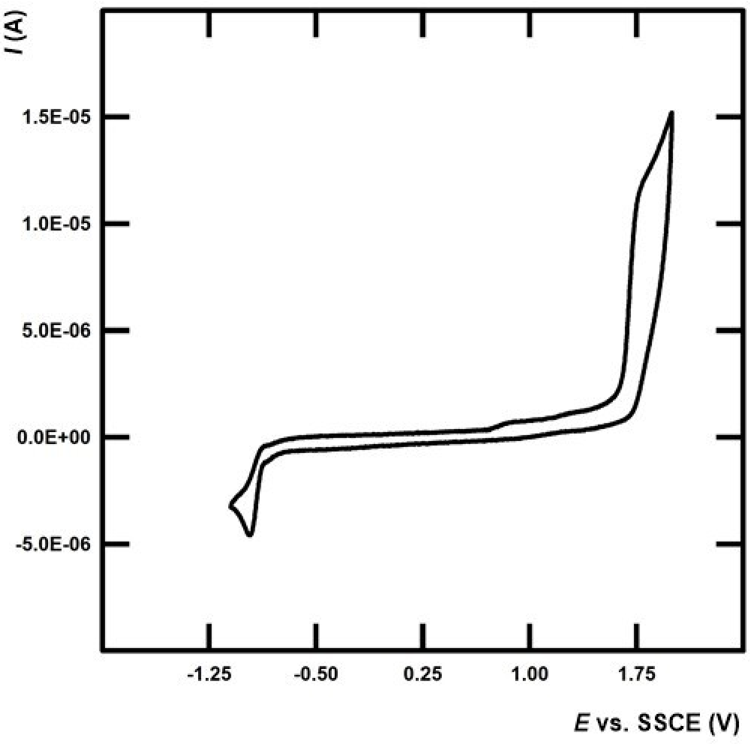

Figure S14. CV diagram of compound **11** at GC electrode in acetonitrile containing 0.1 M Bu_4NBF_4; v=100mV/s.

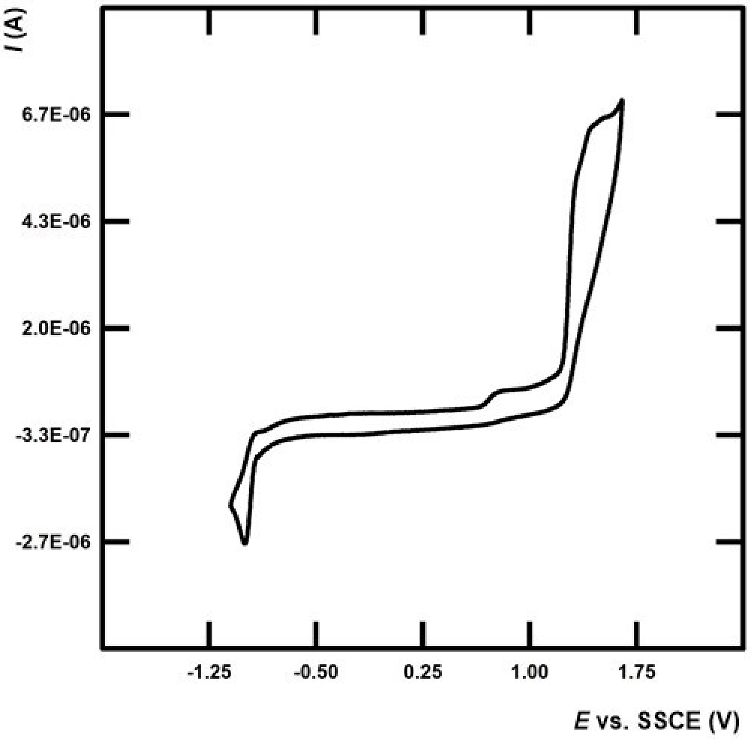

Figure S15. CV diagram of compound **12** at GC electrode in acetonitrile containing 0.1 M Bu_4NBF_4; v=100mV/s.

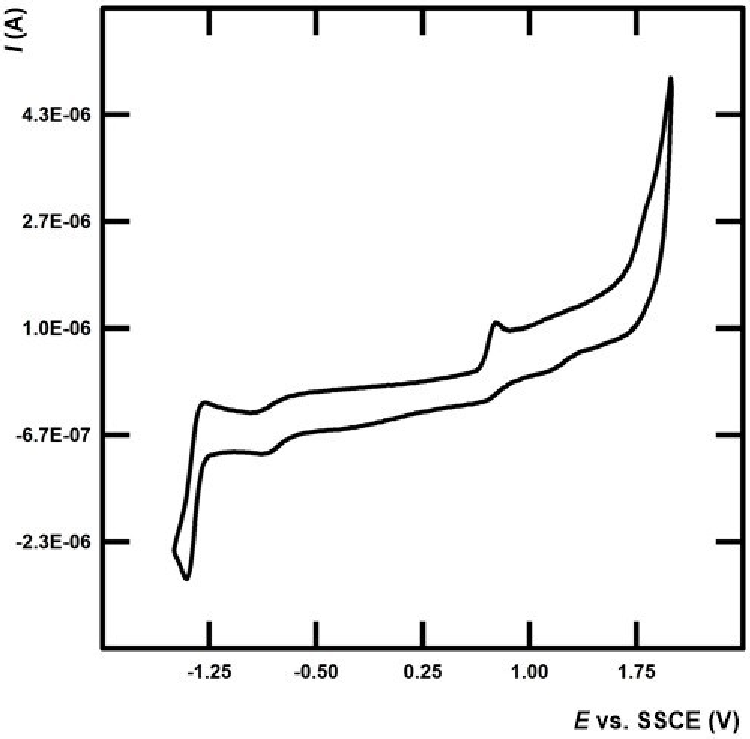

Figure S16. CV diagram of compound **13** at GC electrode in acetonitrile containing 0.1 M Bu_4NBF_4; v=100mV/s.

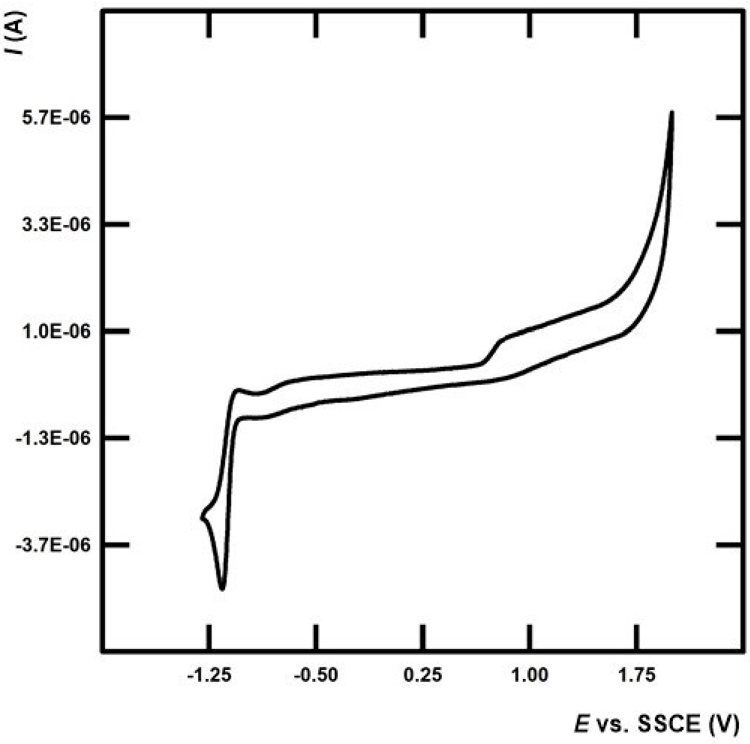

Figure S17. CV diagram of compound **14** at GC electrode in acetonitrile containing 0.1 M Bu_4NBF_4; v=100mV/s.

6. DFT calculations

Table S2. DFT calculated parameters of DPZ derivatives **1**–**14**.

Comp.	$\Delta E^{\bullet+}$ [eV]	$E^{\bullet+}$(αLUMO) [eV]	$E^{\bullet+}$(βHOMO) [eV]	$E^{\bullet+}$(βLUMO) [eV]	$\Delta E^{\bullet-}$ [eV]	$E^{\bullet-}$(αLUMO) [eV]	$E^{\bullet-}$(βHOMO) [eV]	$E^{\bullet-}$(βLUMO) [eV]
**1**	5.57	-3.61	-6.96	-5.27	-3.20	-1.91	-5.20	-2.05
**2**	6.16	-3.98	-7.78	-5.88	-3.36	-2.06	-5.60	-2.17
**3**	5.55	-3.58	-6.53	-5.28	-3.27	-2.02	-5.36	-2.05
**4**	6.28	-3.67	-6.84	-5.70	-3.22	-1.80	-5.91	-1.77
**5**	5.90	-3.43	-6.19	-5.35	-3.20	-1.76	-5.83	-1.74
**6**	5.77	-3.33	-5.96	-5.37	-3.25	-1.85	-5.94	-1.81
**7**	6.98	-4.09	-9.66	-6.73	-2.96	-1.18	-5.78	-1.42
**8**	6.53	-3.83	-8.28	-6.32	-3.11	-1.58	-5.70	-1.64
**9**	6.55	-3.96	-7.30	-6.33	-3.30	-2.23	-6.01	-2.11
**10**	5.97	-3.55	-6.52	-5.75	-3.13	-2.07	-5.57	-1.95
**11**	6.43	-3.57	-6.59	-5.92	-3.33	-1.80	-6.24	-1.69
**12**	5.91	-3.17	-6.59	-5.56	-3.28	-1.71	-5.93	-1.59
**13**	7.01	-3.89	-9.66	-6.79	-3.01	-0.99	-6.03	-1.07
**14**	6.63	-3.74	-8.01	-6.46	-3.15	-1.52	-5.88	-1.40

Comp.	ΔE^{2-} [eV]	E^{2-}(HOMO) [eV]	E^{2-}(LUMO) [eV]	ΔE^{2+} [eV]	E^{2+}(HOMO) [eV]	E^{2+}(LUMO) [eV]	ΔE^{T} [eV]	E^{T}(αLUMO) [eV]	E^{T}(βLUMO) [eV]
**1**	-5.53	-2.61	-1.16	11.81	-7.91	-5.92	1.66	-2.68	-4.52
**2**	-5.82	-2.75	-1.35	13.09	-8.83	-6.61	1.90	-2.95	-4.92
**3**	-5.98	-2.93	-1.55	11.72	-7.43	-5.88	1.68	-2.75	-4.56
**4**	-5.55	-2.84	-1.17	12.91	-7.70	-6.28	2.28	-2.75	-5.05
**5**	-5.45	-2.75	-1.14	11.99	-6.81	-5.74	2.08	-2.60	-4.73
**6**	-5.67	-2.88	-1.28	11.78	-6.54	-5.70	2.01	-2.61	-4.72
**7**	-4.72	-2.10	-0.22	15.26	-10.76	-8.03	3.02	-2.39	-5.19
**8**	-5.06	-2.28	-0.58	14.25	-9.85	-7.51	2.32	-2.49	-5.15
**9**	-5.39	-2.45	-1.54	14.15	-8.14	-7.42	2.49	-3.18	-5.55
**10**	-5.29	-2.47	-1.29	12.84	-7.28	-6.65	2.20	-2.84	-5.09
**11**	-5.40	-2.45	-1.09	13.32	-7.35	-6.67	2.44	-2.58	-5.31
**12**	-5.24	-2.41	-1.01	12.39	-6.57	-6.07	2.20	-2.33	-4.90
**13**	-4.46	-1.84	-0.06	15.73	-10.76	-8.57	2.79	-2.37	-5.56
**14**	-4.81	-2.04	-0.56	14.69	-9.26	-7.92	2.53	-2.64	-5.47

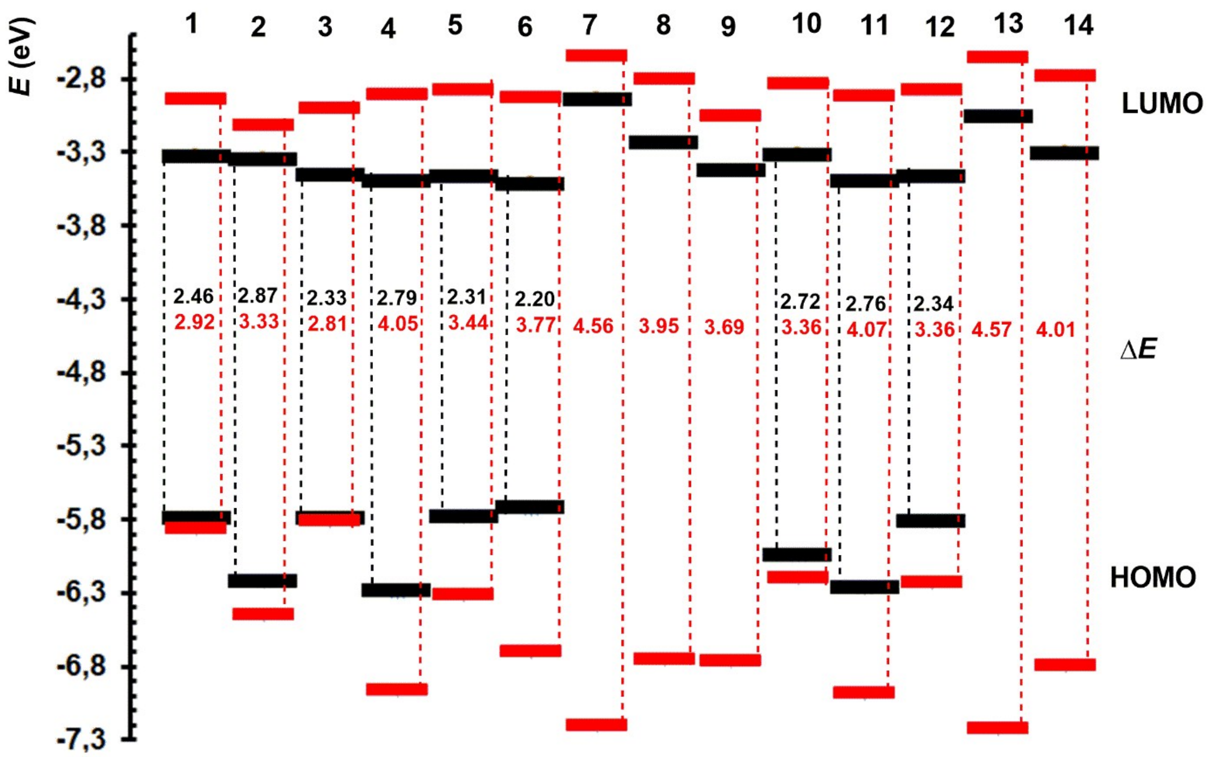

Figure S18. Energy level diagram of ground state HOMO/LUMO levels for compounds **1**–**14**.

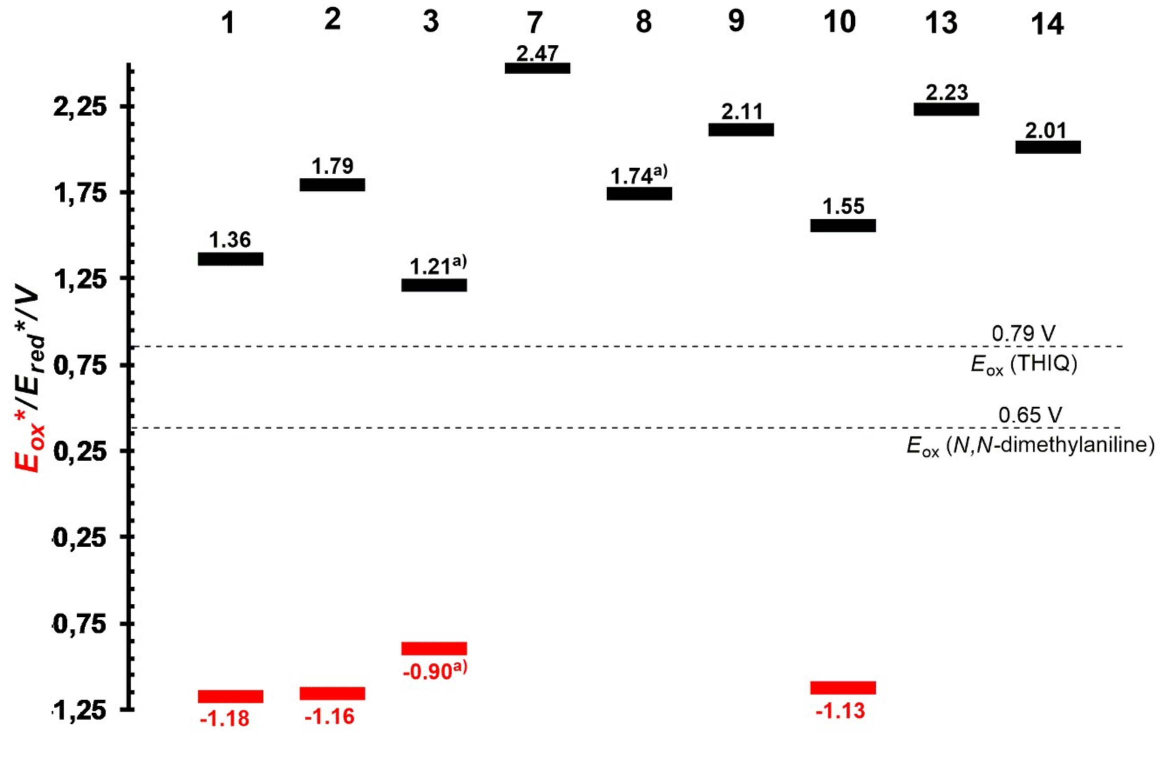

Figure S19. Energy level diagram of excited state reduction/oxidation potentials (acetonitrile).
[a] Emission maxima taken from the spectra measured in DCM

6.1. Representative localizations of frontier molecular orbitals

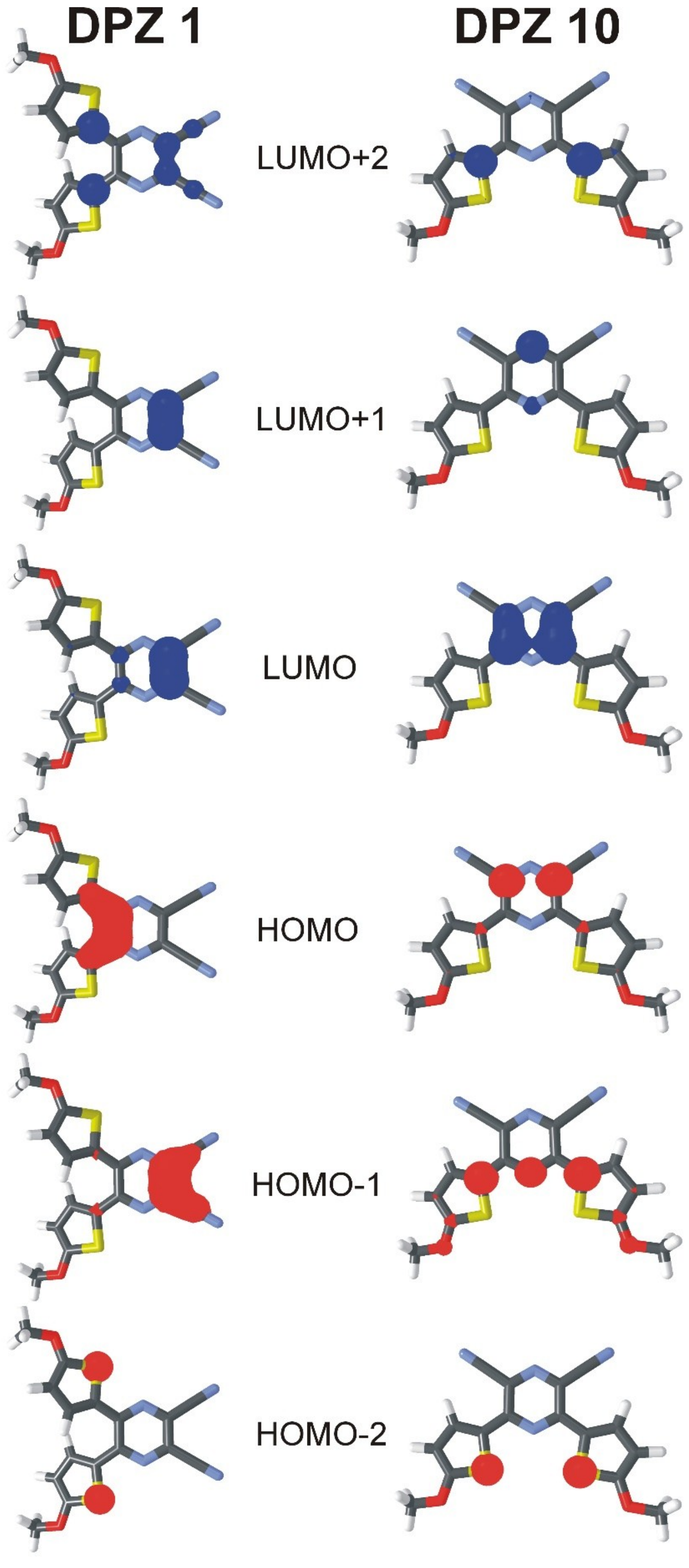

Figure S20. HOMO(-1/-2) and LUMO(+1/+2) localizations in DPZs **1** and **10**.

6.2. Frontier molecular orbitals and spinorbitals of DPZs 1–14

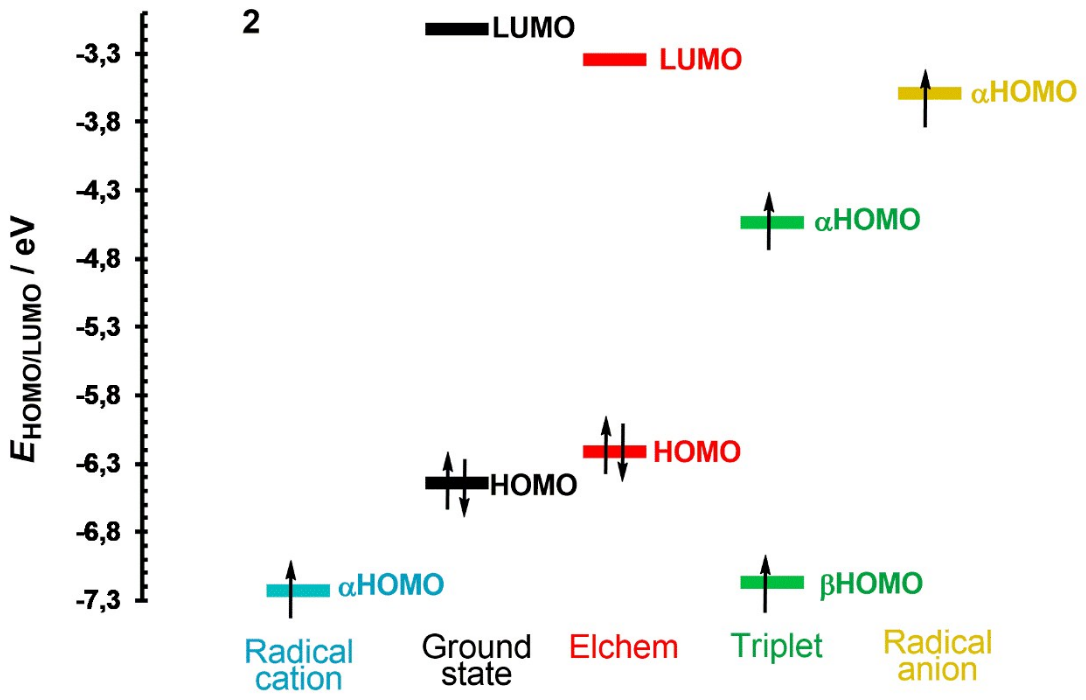

Figure S21. Energy level diagram of compound **2**.

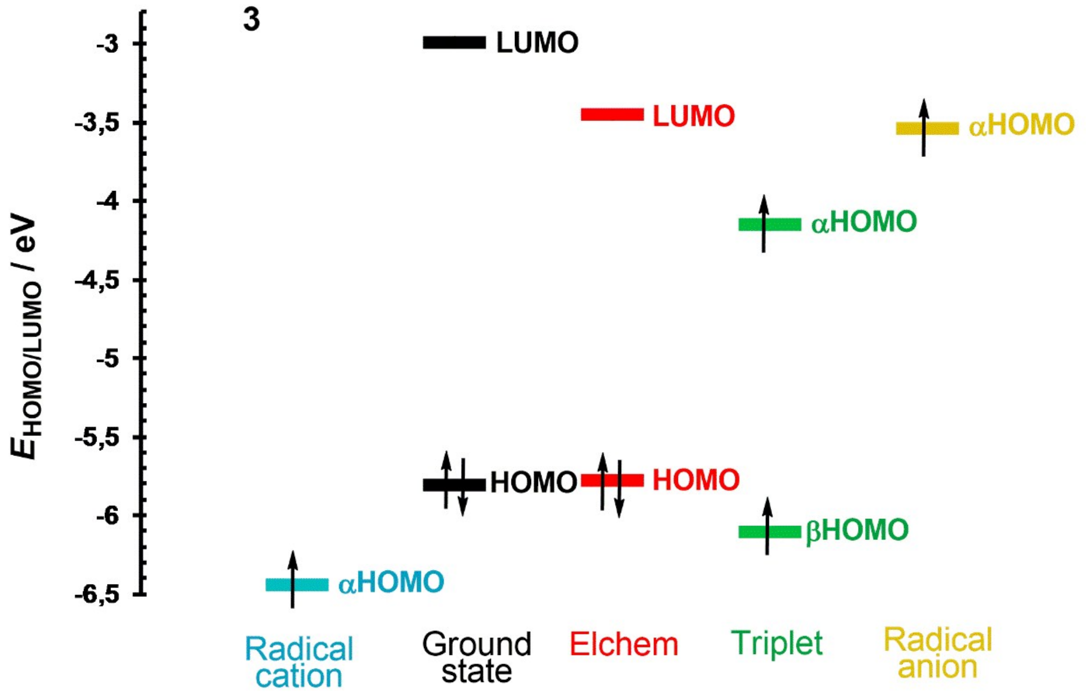

Figure S22. Energy level diagram of compound **3**.

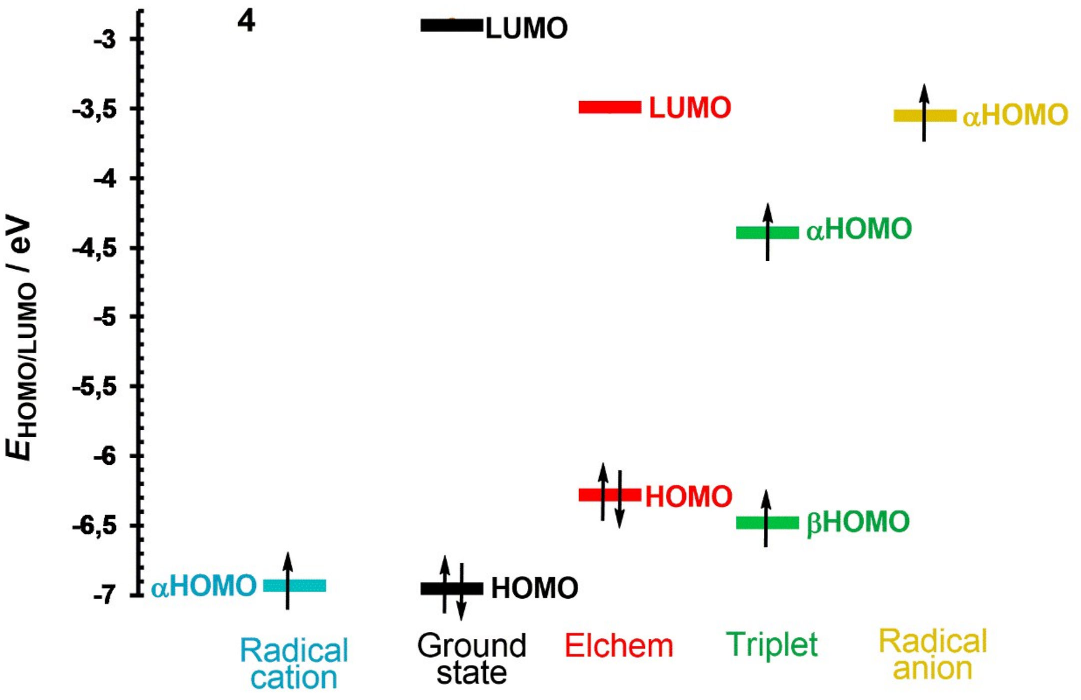

Figure S23. Energy level diagram of compound **4**.

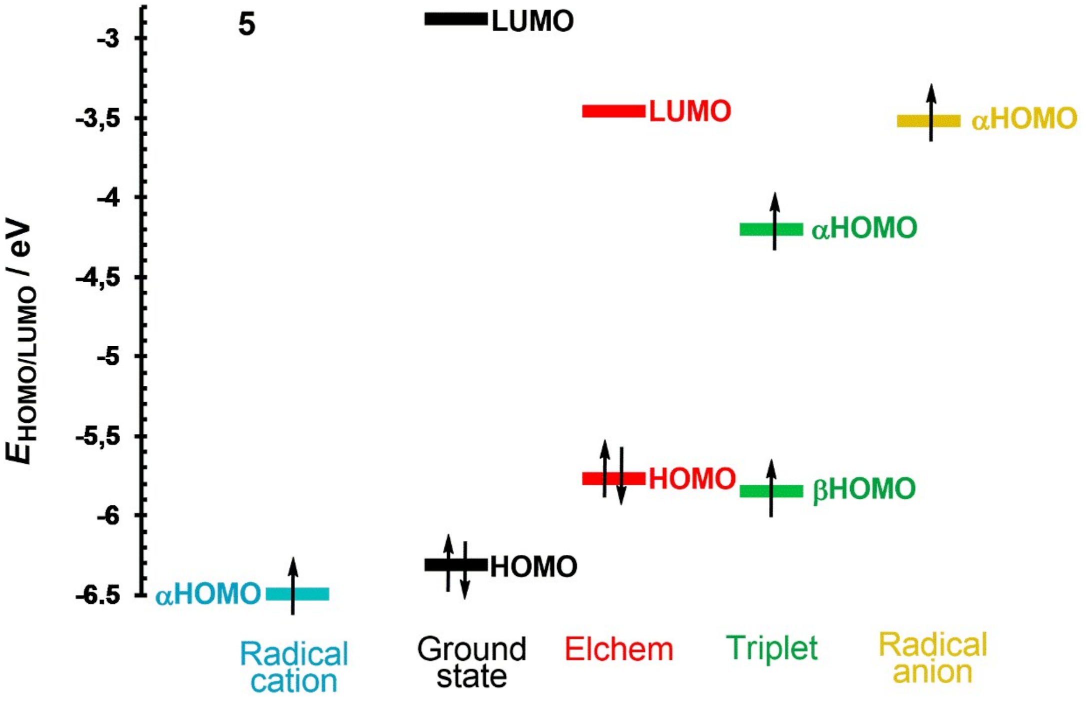

Figure S24. Energy level diagram of compound **5**.

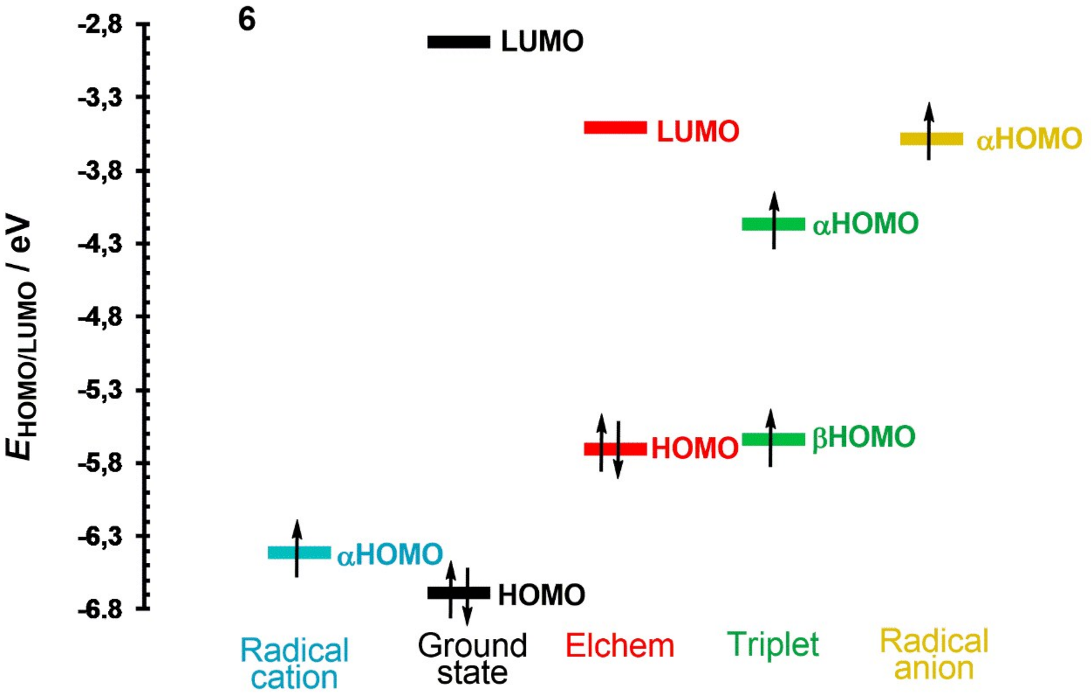

Figure S25. Energy level diagram of compound **6**.

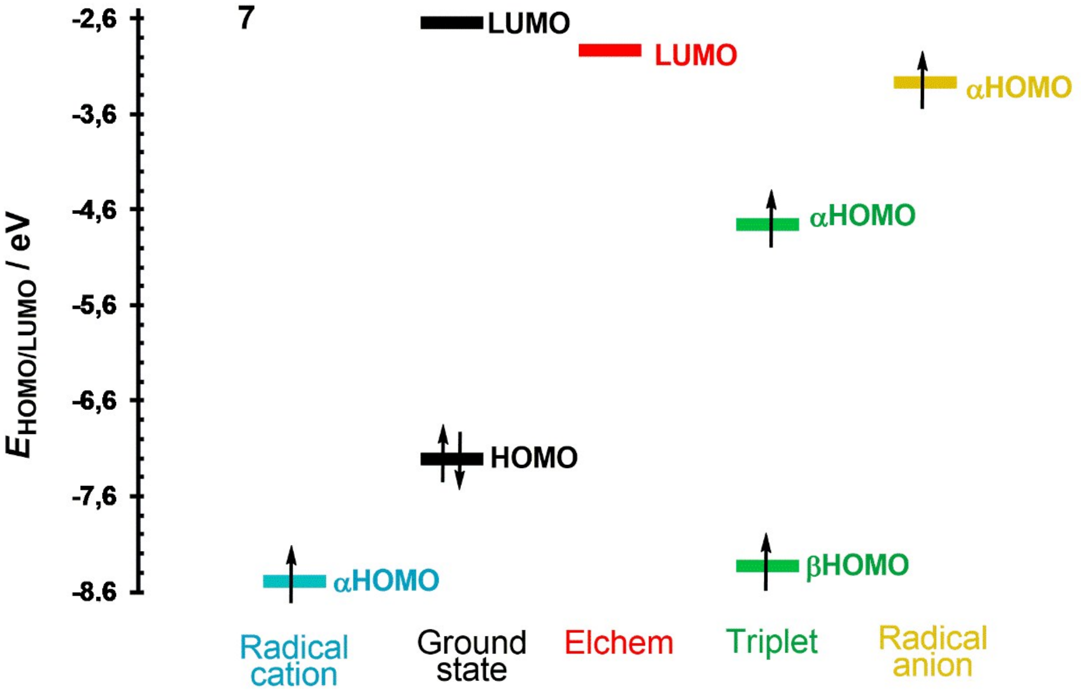

Figure S26. Energy level diagram of compound **7**.

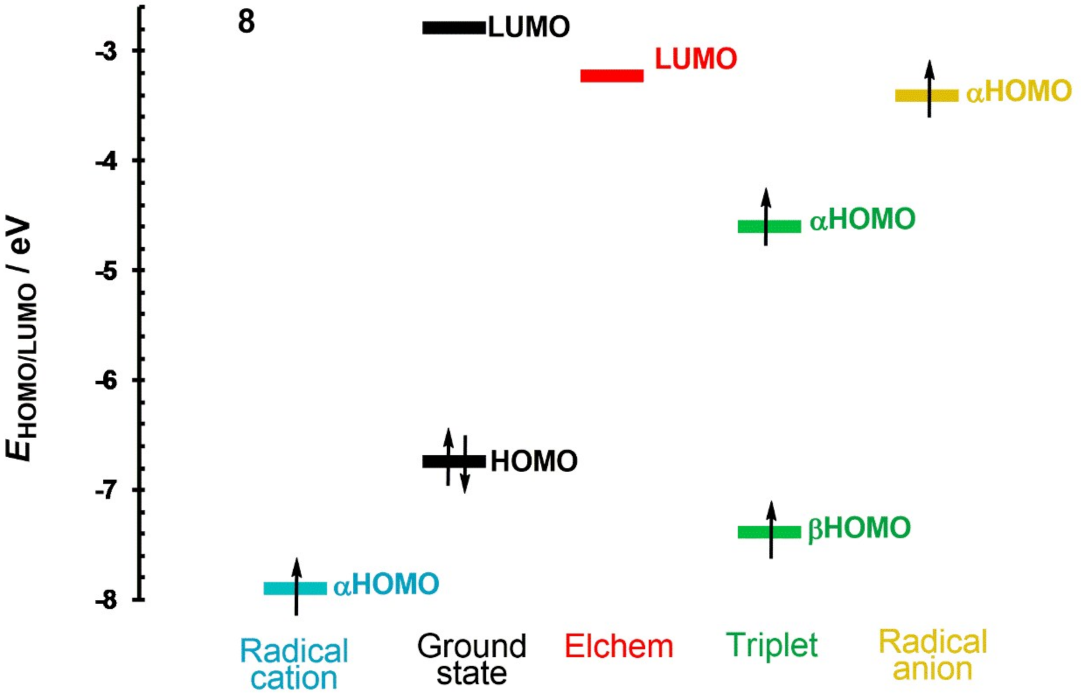

Figure S27. Energy level diagram of compound **8**.

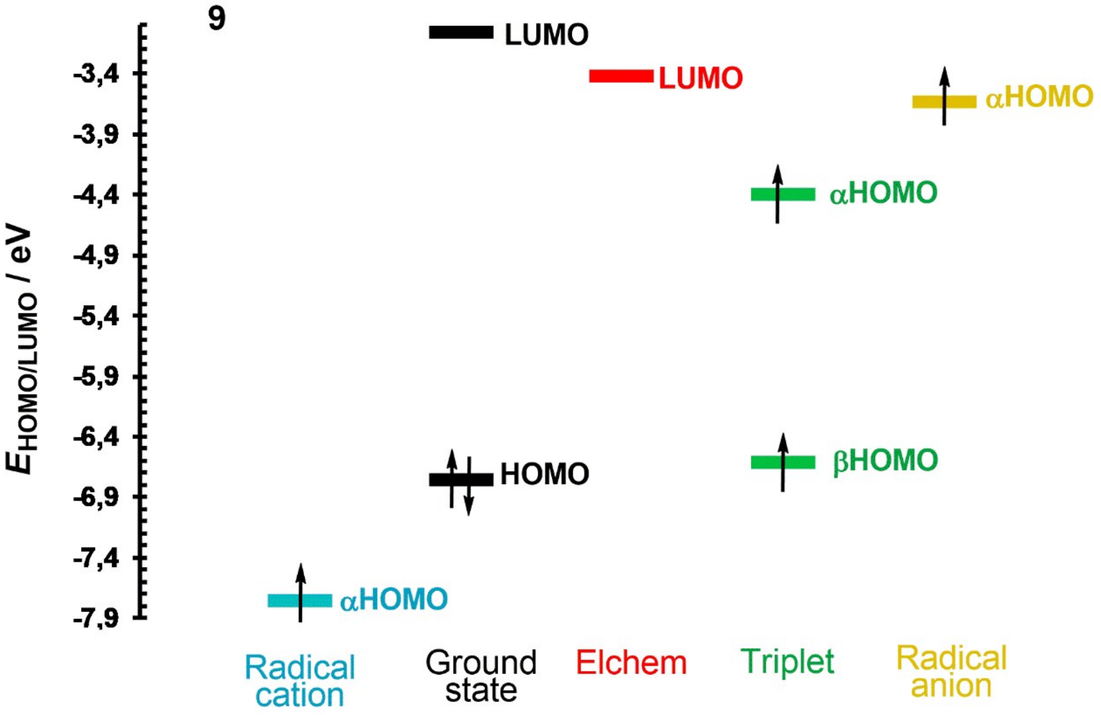

Figure S28. Energy level diagram of compound **9**.

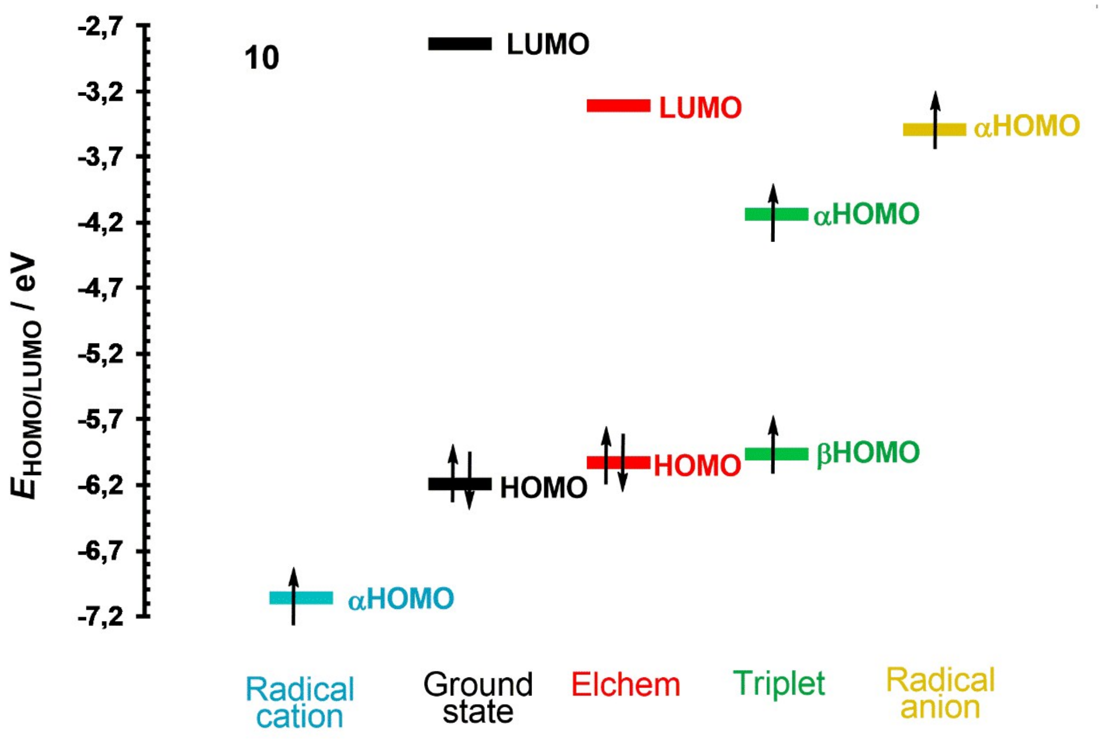

Figure S29. Energy level diagram of compound **10**.

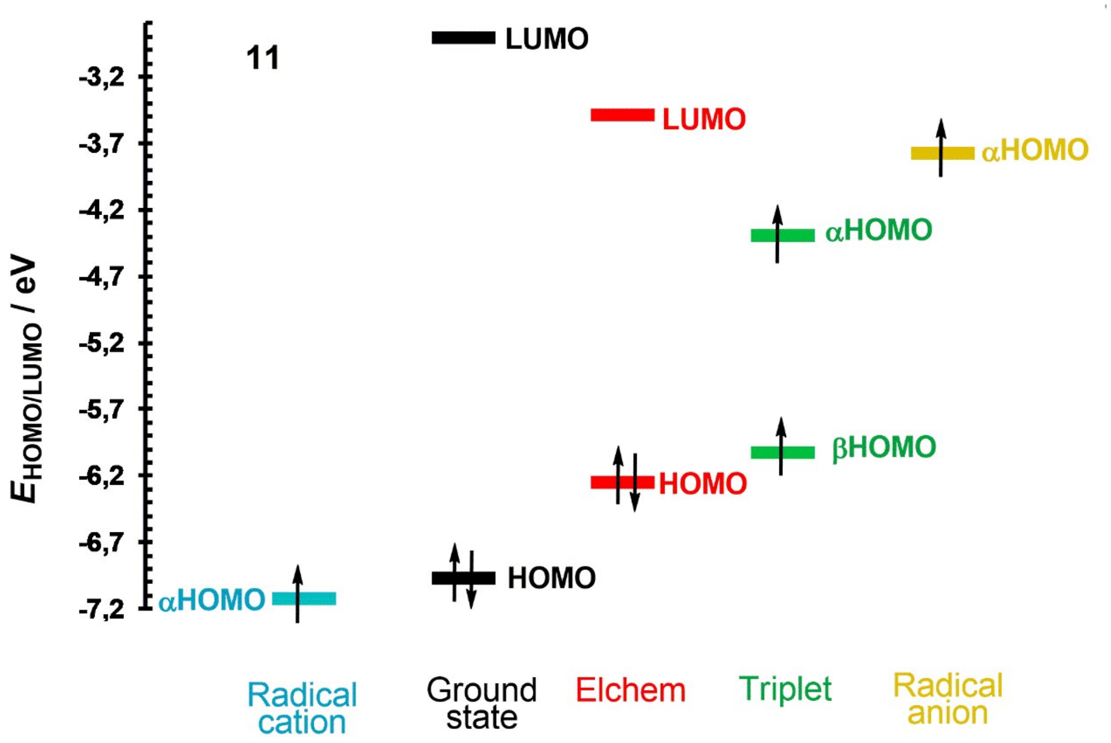

Figure S30. Energy level diagram of compound **11**.

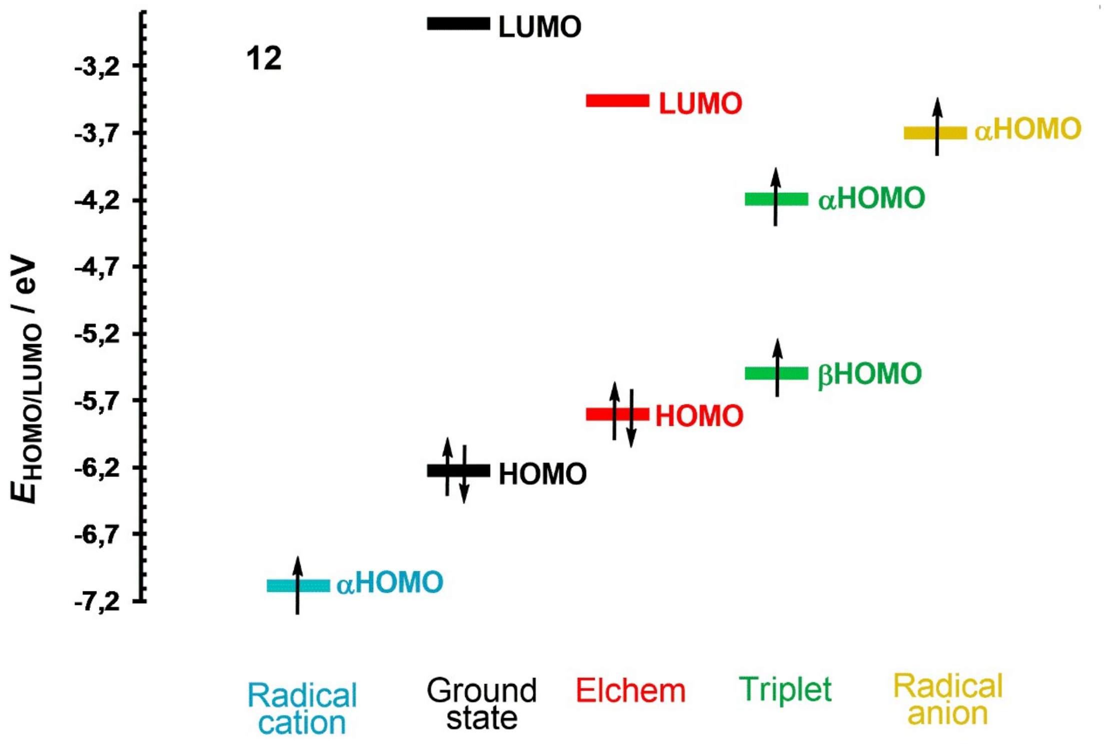

Figure S31. Energy level diagram of compound **12**.

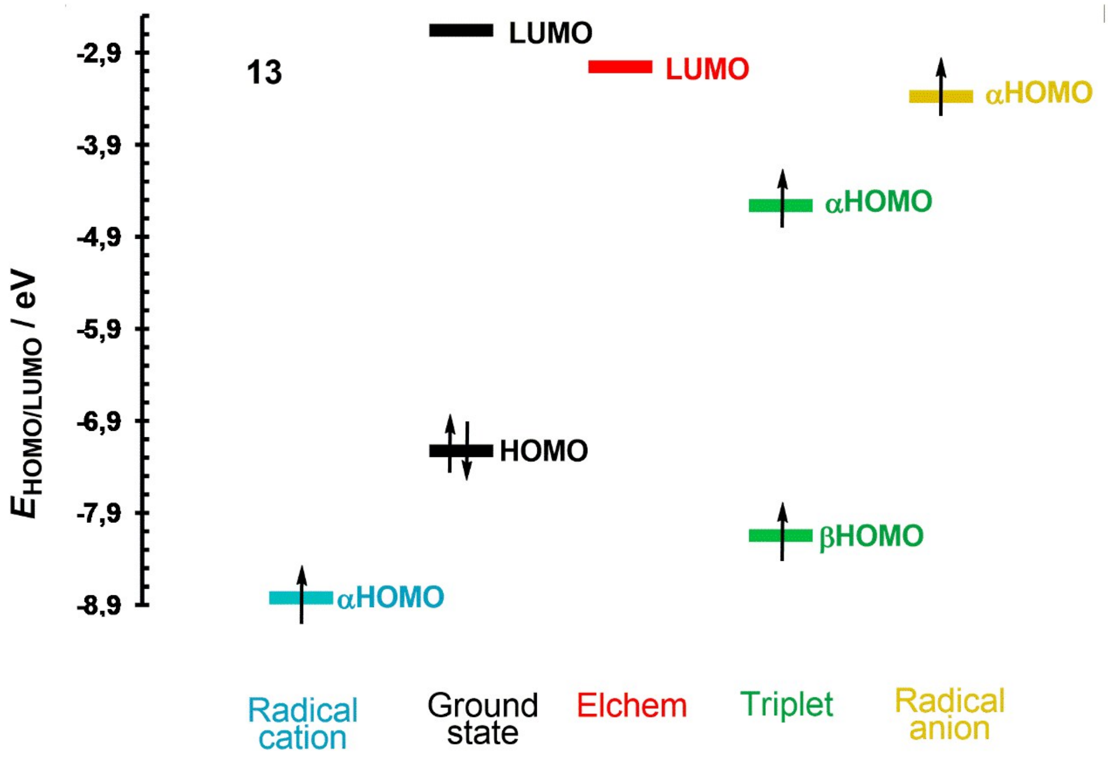

Figure S32. Energy level diagram of compound **13**.

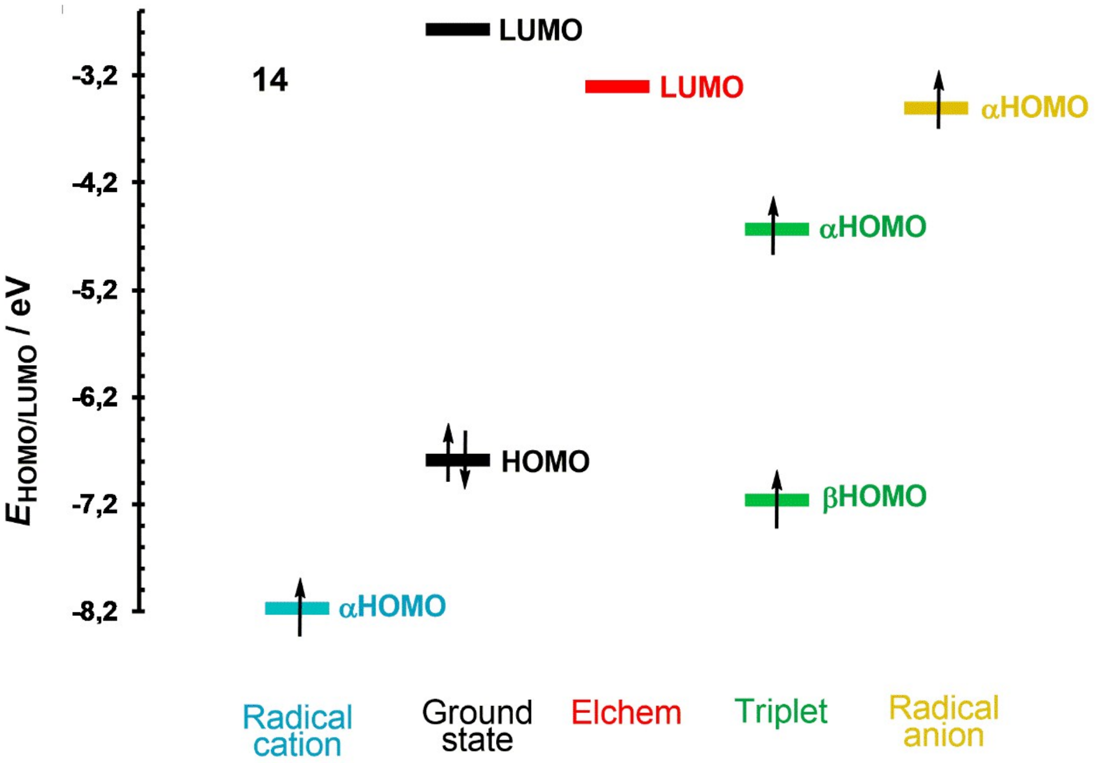

Figure S33. Energy level diagram of compound **14**.

6.3. Calculated spectra of DPZ derivatives 1–14

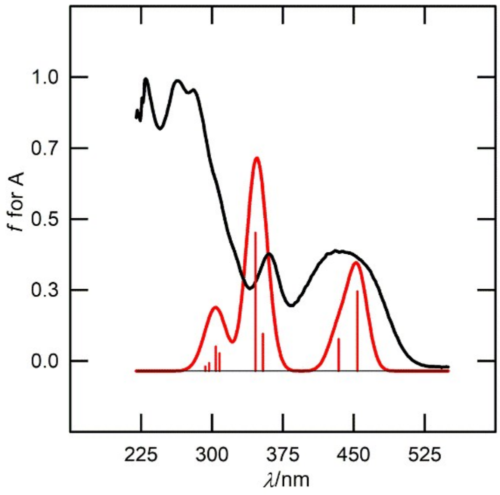

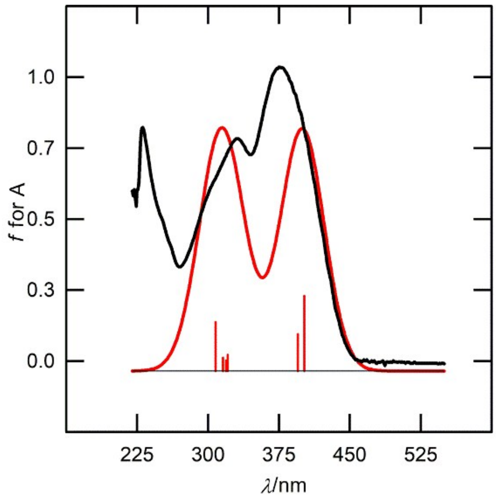

Figure S34. Experimental (black) vs. DFT calculated (red) Uv Vis absorption spectra of **1**.

Figure S35. Experimental (black) vs. DFT calculated (red) Uv Vis absorption spectra of **2**.

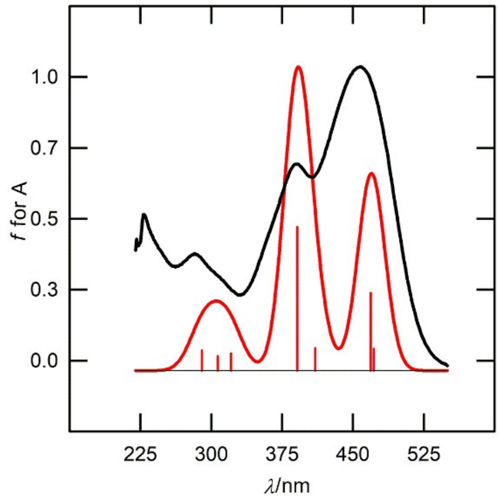

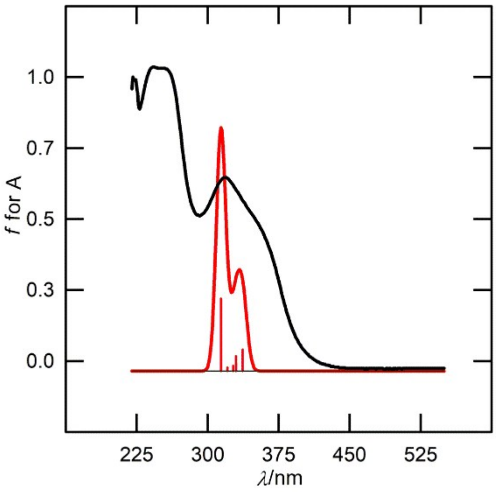

Figure S36. Experimental (black) vs. DFT calculated (red) Uv Vis absorption spectra of **3**.

Figure S37. Experimental (black) vs. DFT calculated (red) Uv Vis absorption spectra of **4**.

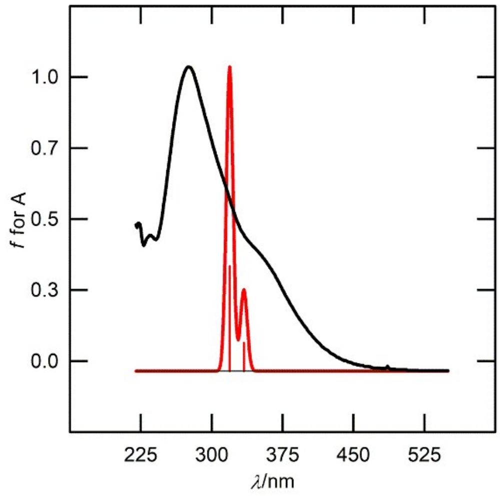

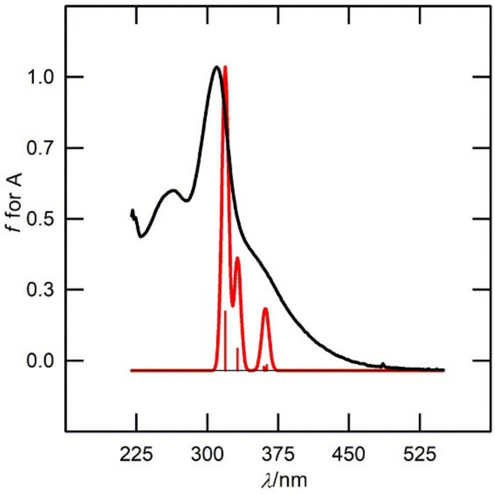

Figure S38. Experimental (black) vs. DFT calculated (red) Uv Vis absorption spectra of **5**.

Figure S39. Experimental (black) vs. DFT calculated (red) Uv Vis absorption spectra of **6**.

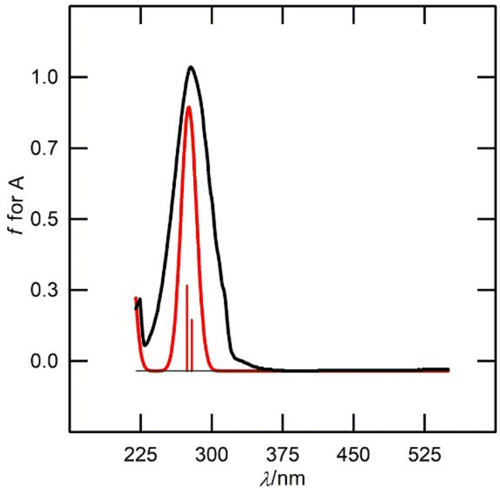

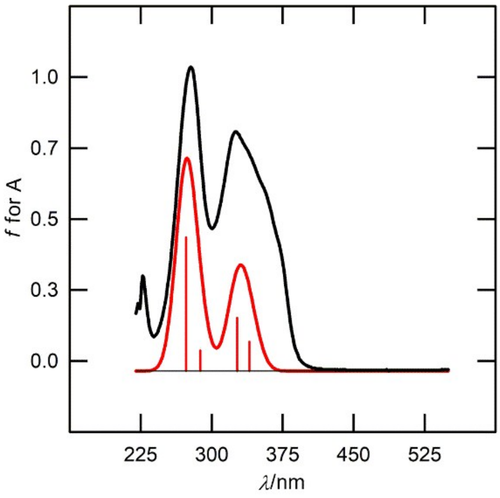

Figure S40. Experimental (black) vs. DFT calculated (red) Uv Vis absorption spectra of **7**. **Figure S41.** Experimental (black) vs. DFT calculated (red) Uv Vis absorption spectra of **8**.

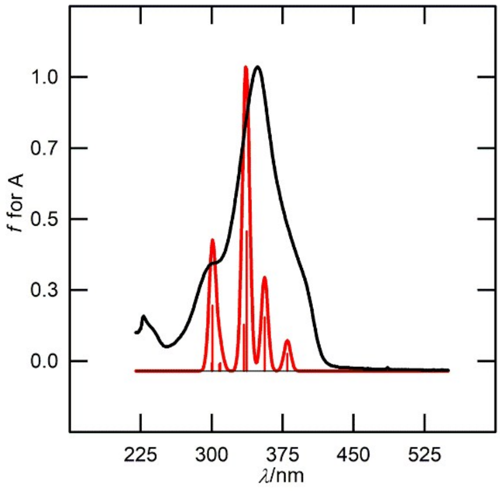

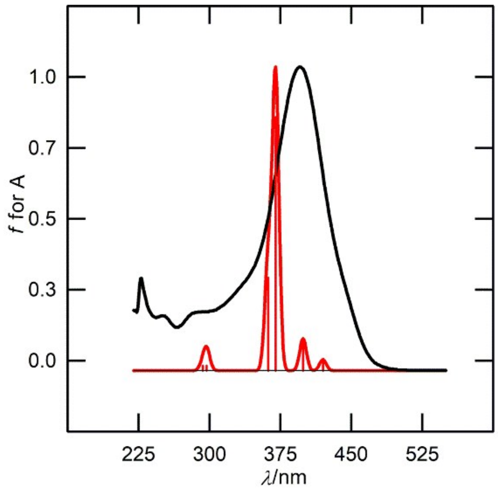

Figure S42. Experimental (black) vs. DFT calculated Uv Vis absorption spectra of **9**. **Figure S43**. Experimental (black) vs. DFT calculated Uv Vis absorption spectra of **10**.

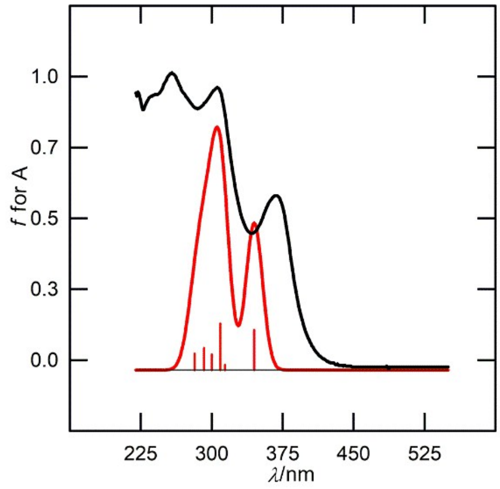

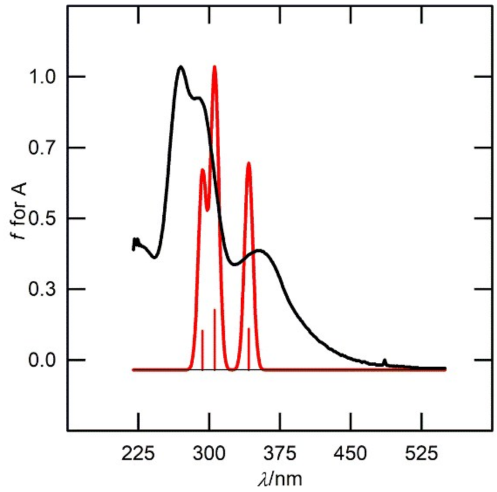

Figure S44. Experimental (black) vs. DFT calculated Uv Vis absorption spectra of **11**. **Figure S45.** Experimental (black) vs. DFT calculated Uv Vis absorption spectra of **12**.

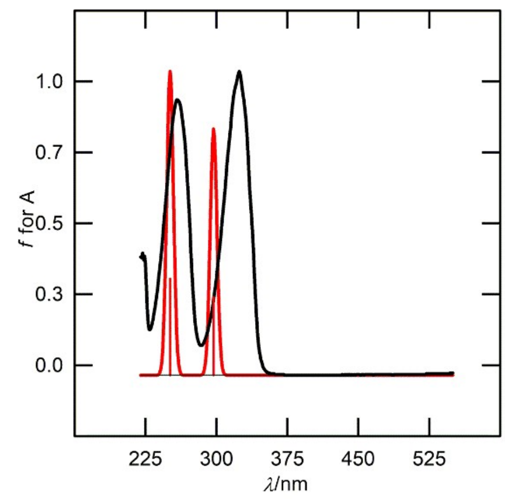

Figure S46. Experimental (black) vs. DFT calculated Uv Vis absorption spectra of **13**.

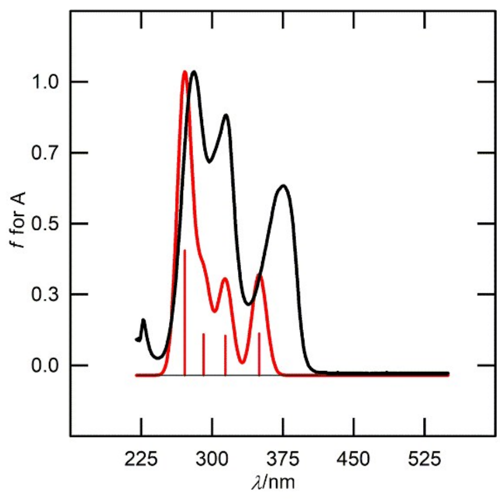

Figure S47. Experimental (black) vs. DFT calculated Uv Vis absorption spectra of **14**.

7. ^{1}H and ^{13}C NMR spectra

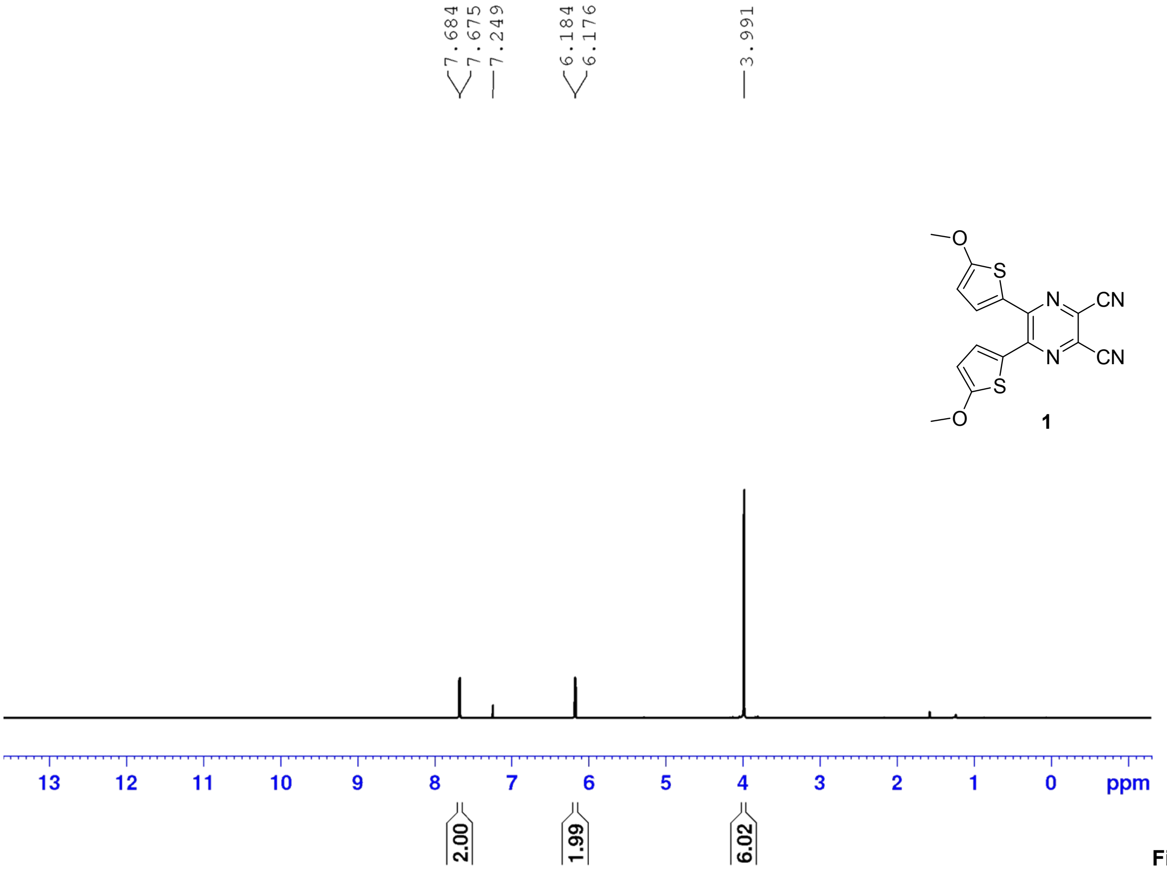

Figure S48. ^{1}H NMR spectrum of chromophore **1** (400 MHz, $CDCl_3$, 25 °C).

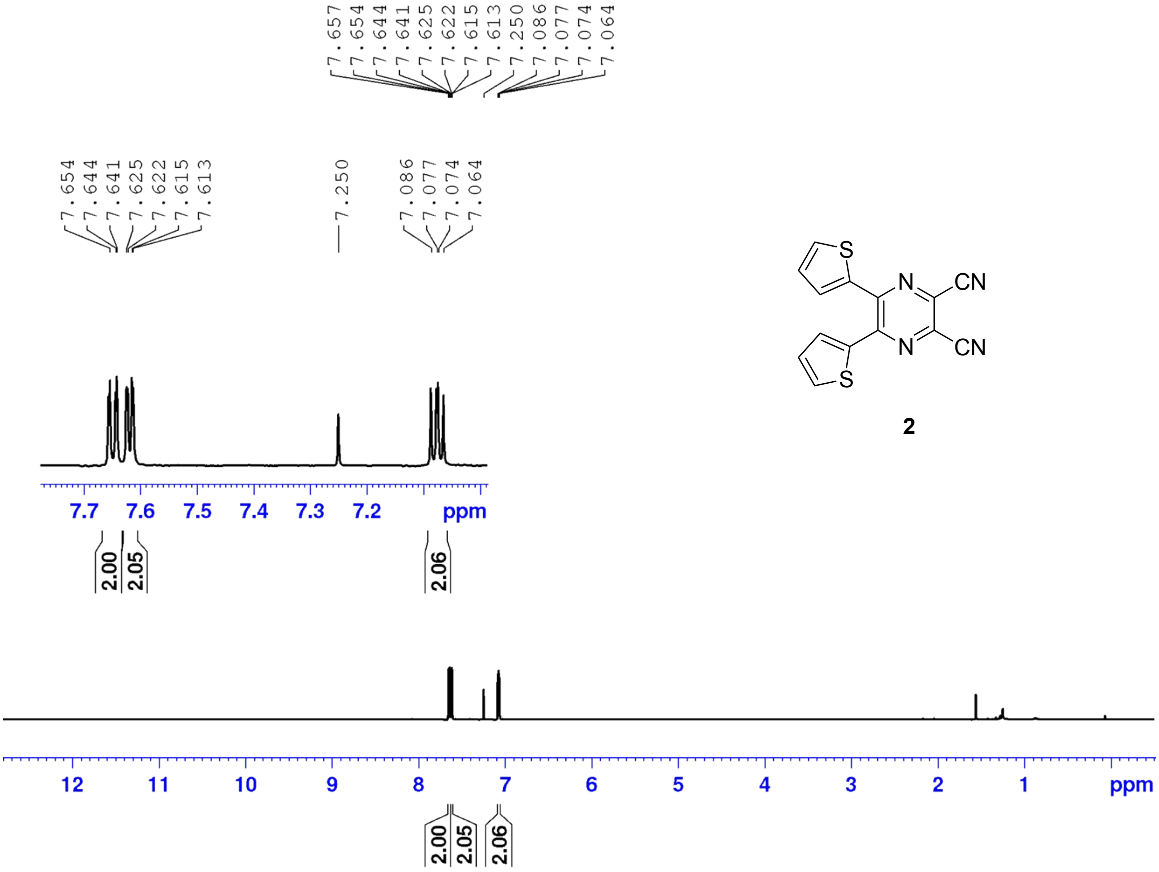

Figure S49. ^{1}H NMR spectrum of chromophore **2** (400 MHz, $CDCl_3$, 25 °C).

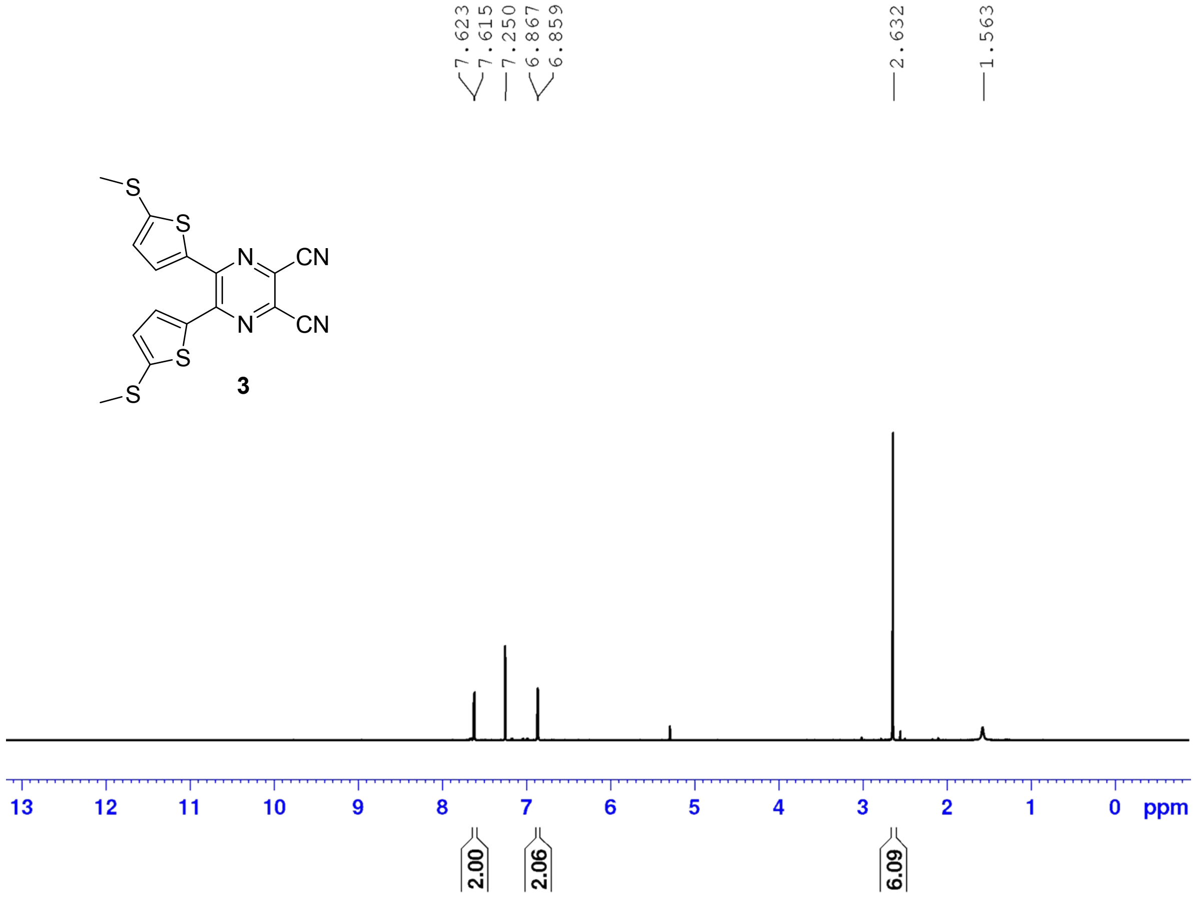

Figure S50. ^{1}H NMR spectrum of chromophore **3** (500 MHz, $CDCl_3$, 25 °C).

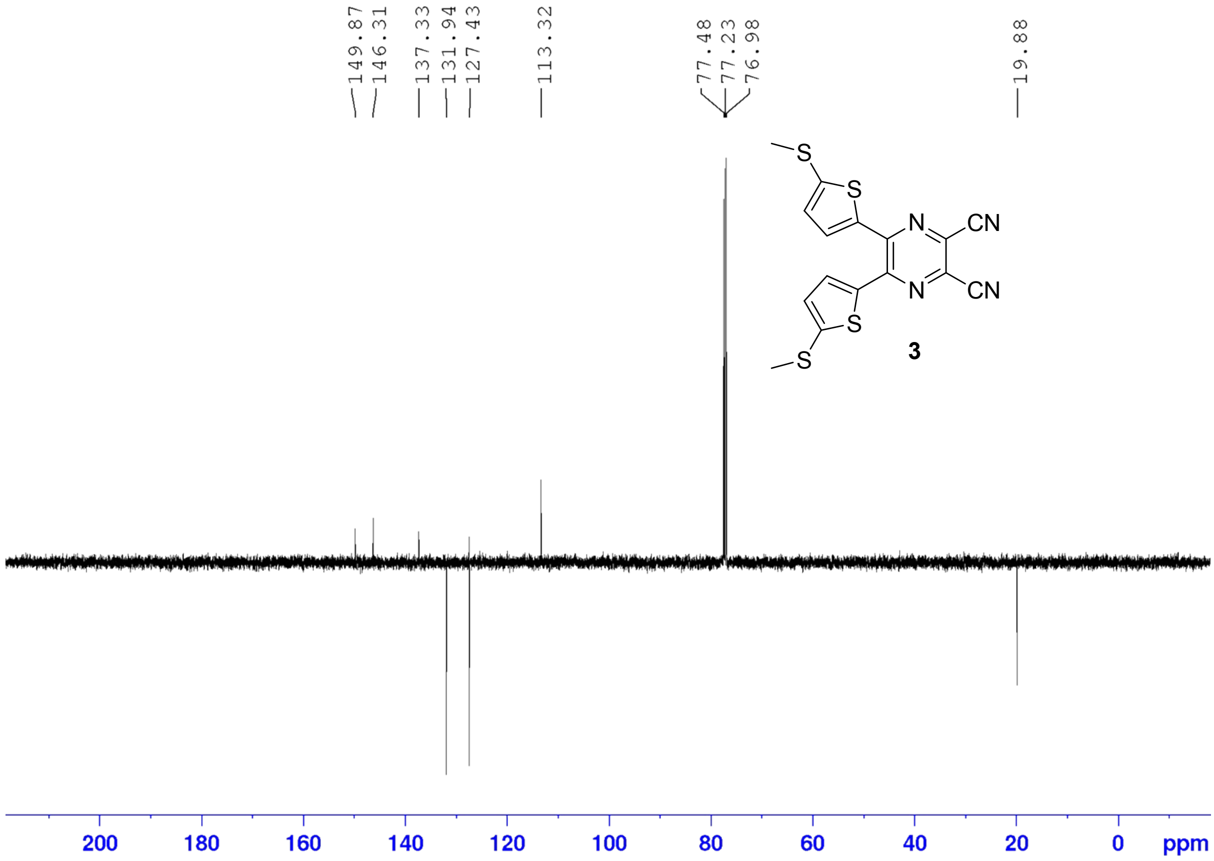

Figure S51. ^{13}C NMR APT spectrum of chromophore **3** (125 MHz, $CDCl_3$, 25 °C).

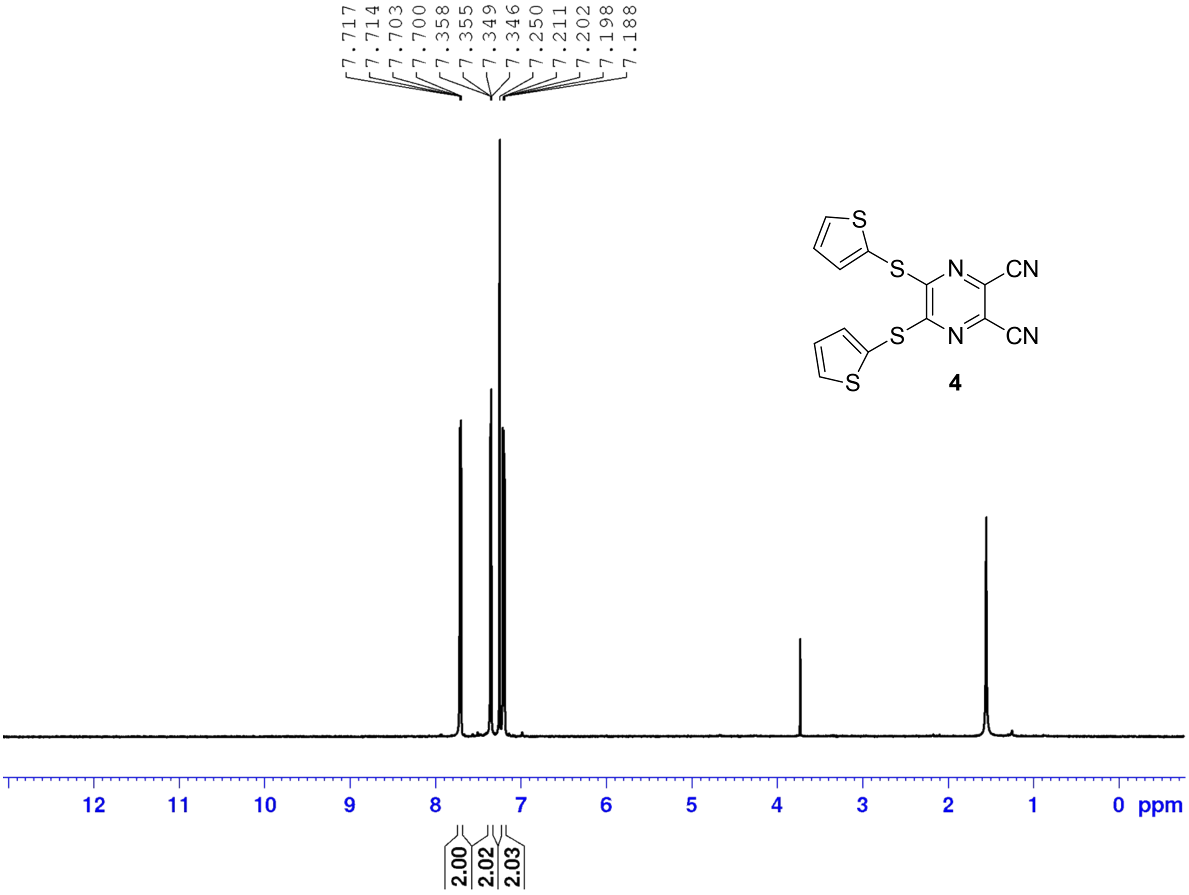

Figure S52. ^{1}H NMR spectrum of chromophore **4** (400 MHz, $CDCl_3$, 25 °C).

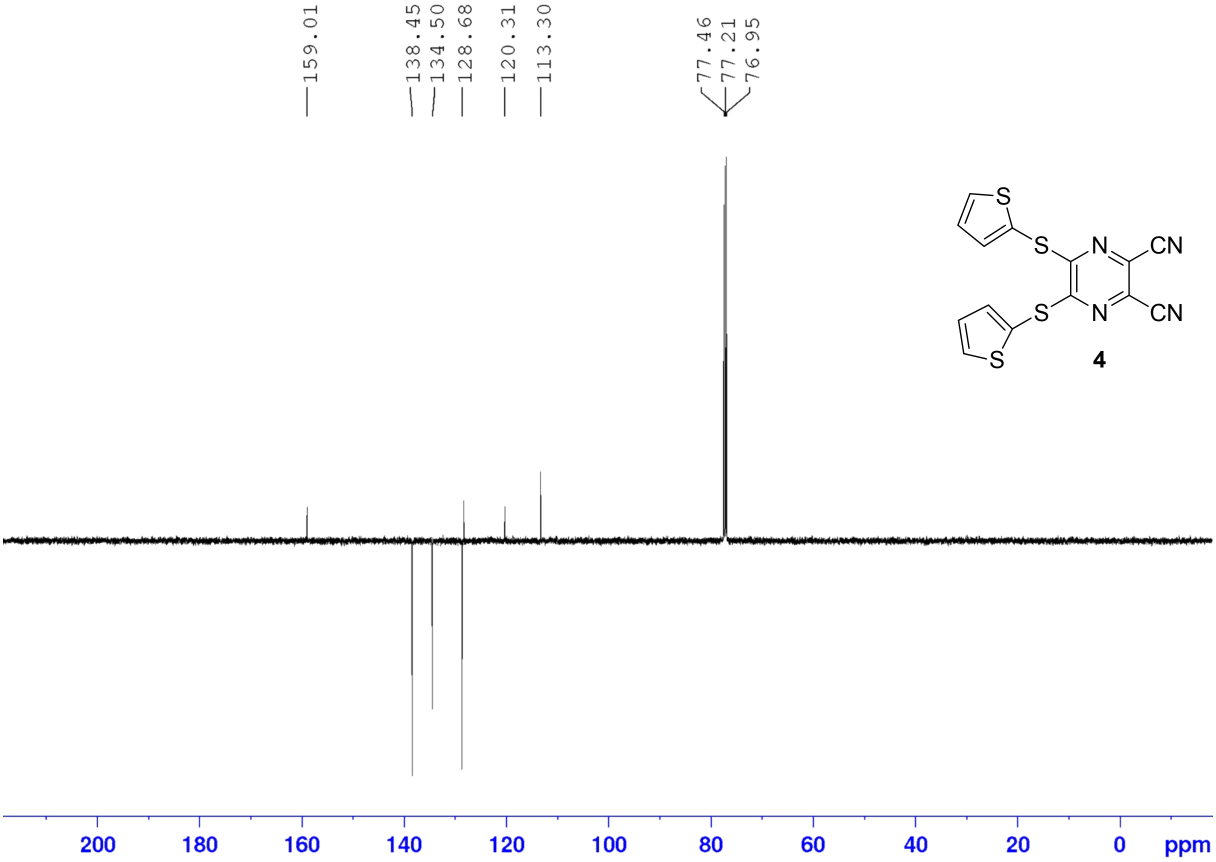

Figure S53. ^{13}C NMR APT spectrum of chromophore **4** (125 MHz, $CDCl_3$, 25 °C).

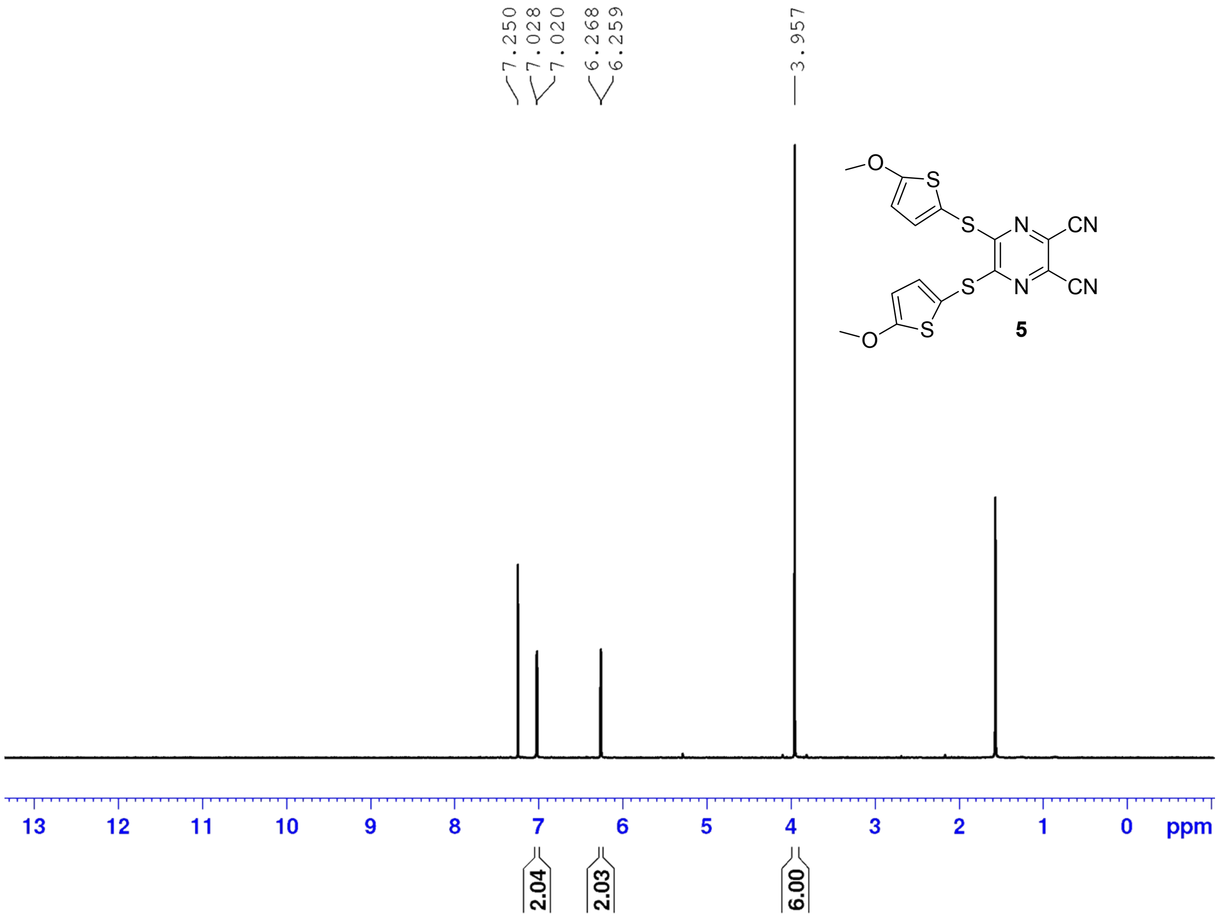

Figure S54. ^{1}H NMR spectrum of chromophore **5** (500 MHz, $CDCl_3$, 25 °C).

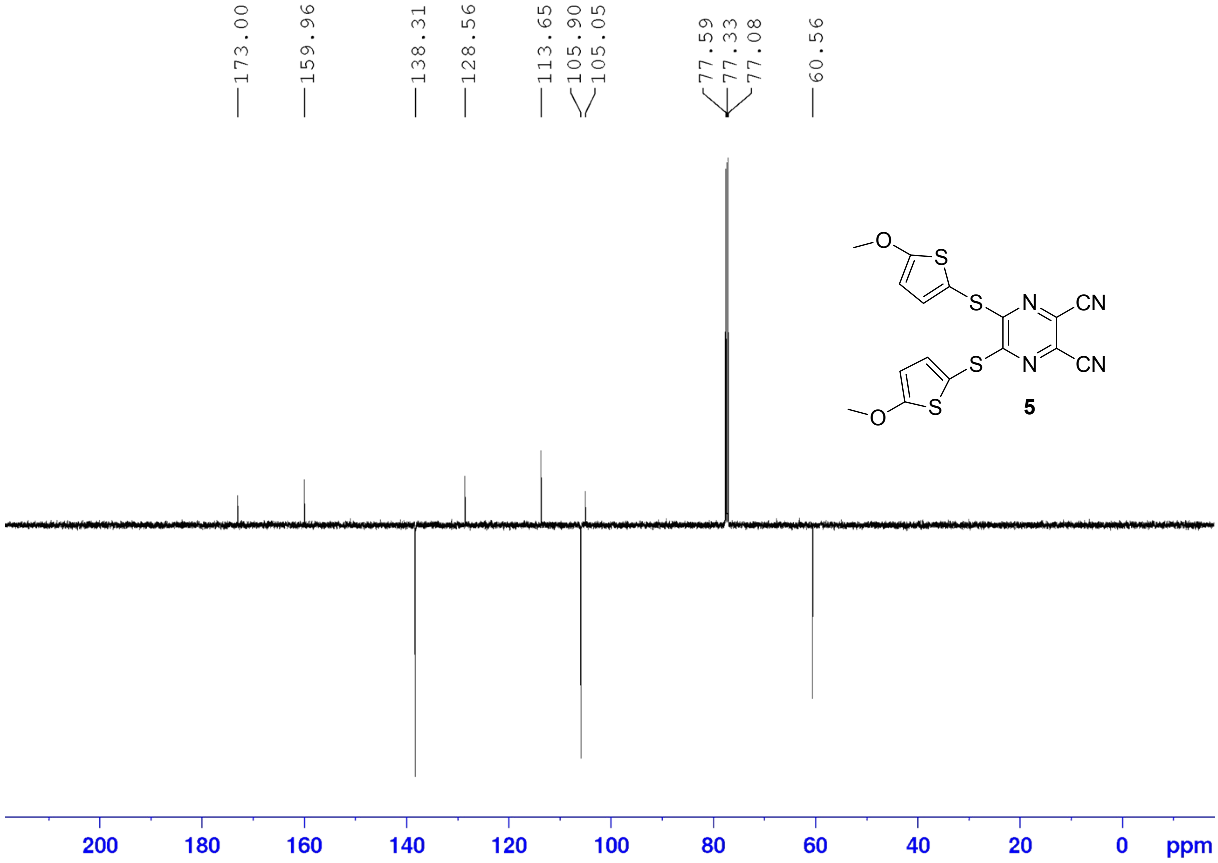

Figure S55. ^{13}C NMR APT spectrum of chromophore **5** (125 MHz, $CDCl_3$, 25 °C).

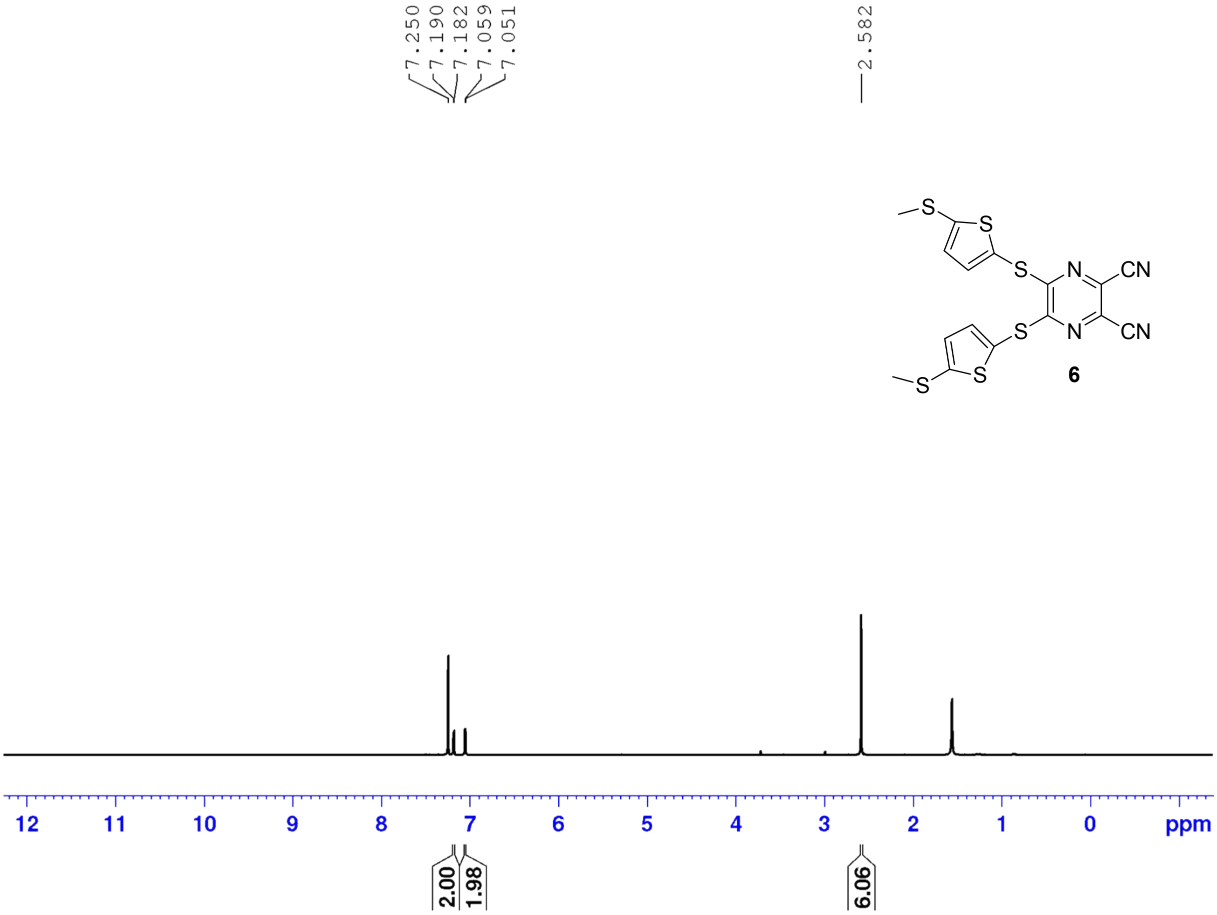

Figure S56. ^{1}H NMR spectrum of chromophore **6** (500 MHz, $CDCl_3$, 25 °C).

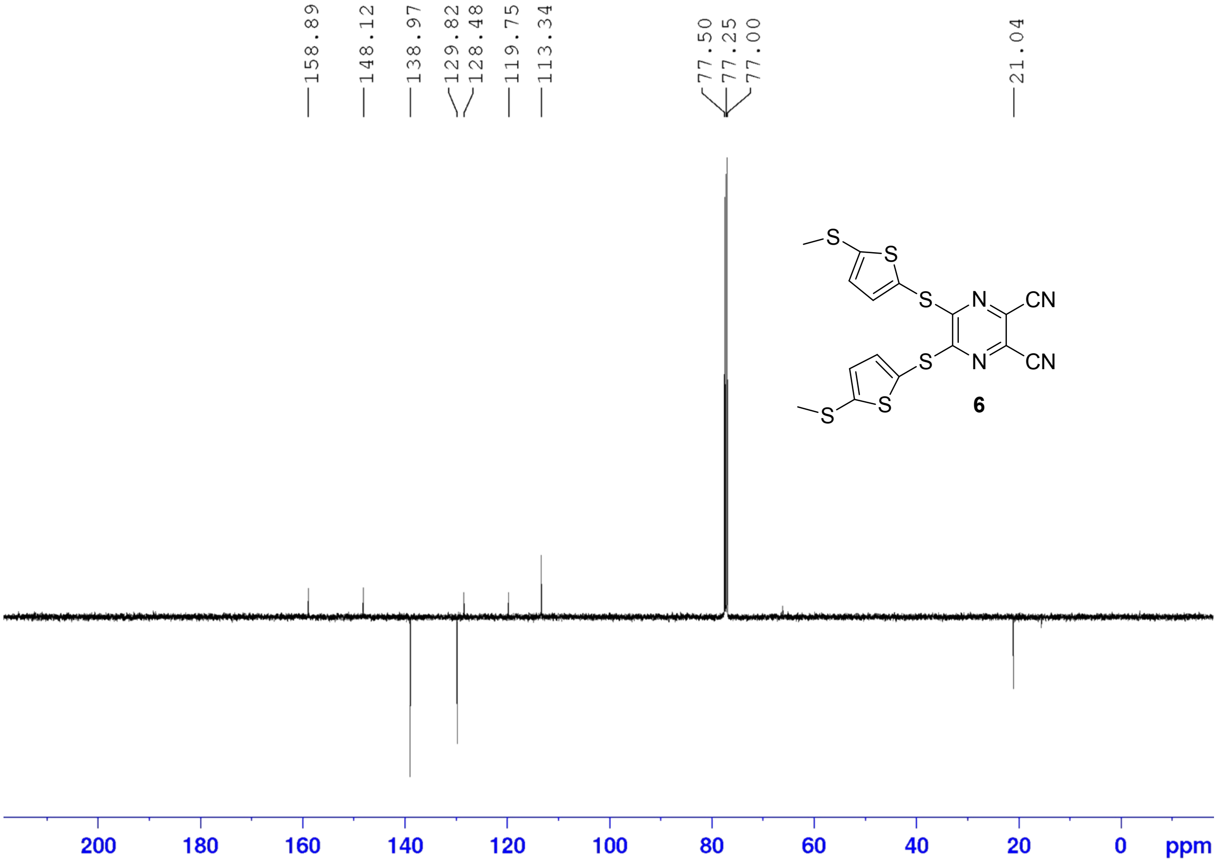

Figure S57. ^{13}C NMR APT spectrum of chromophore **6** (125 MHz, $CDCl_3$, 25 °C).

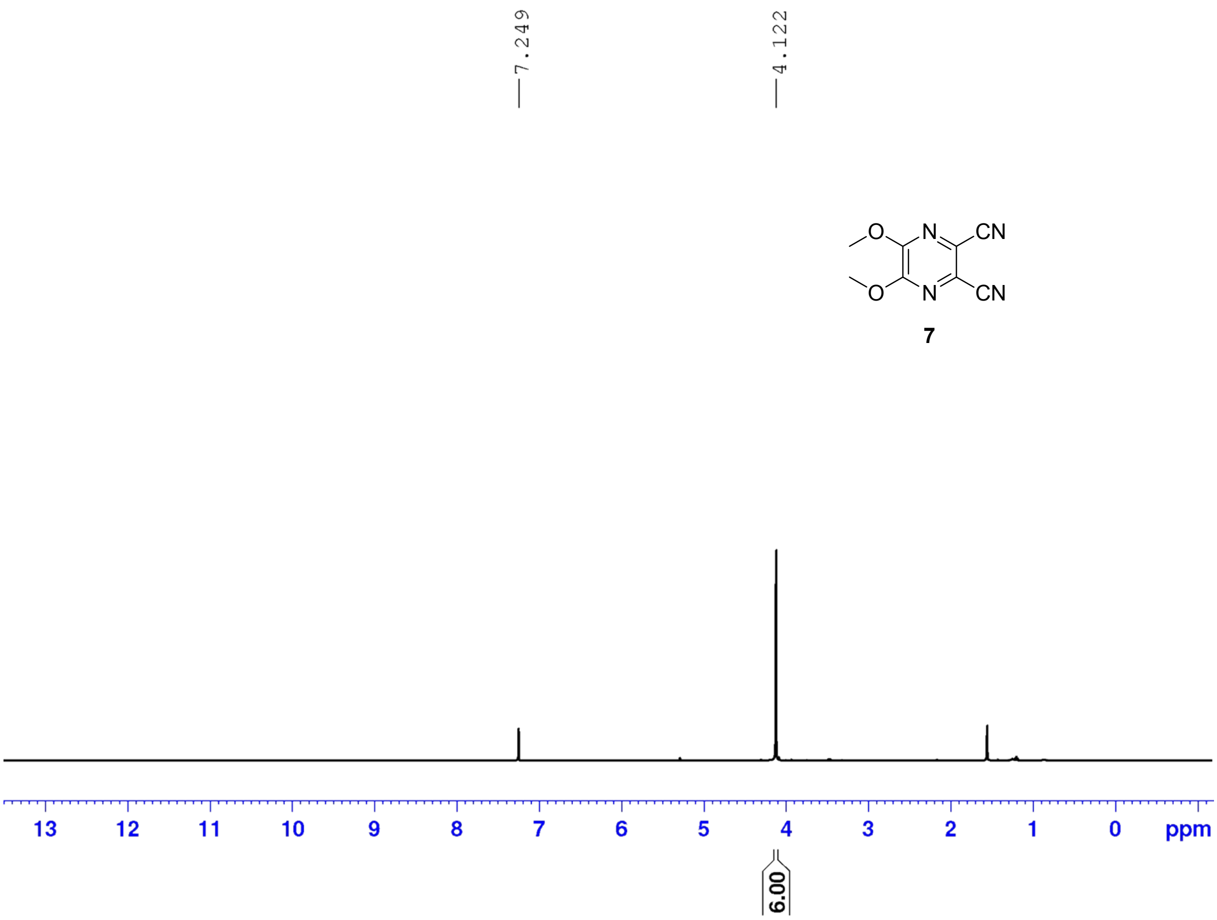

Figure S58. ^{1}H NMR spectrum of chromophore **7** (400 MHz, $CDCl_3$, 25 °C).

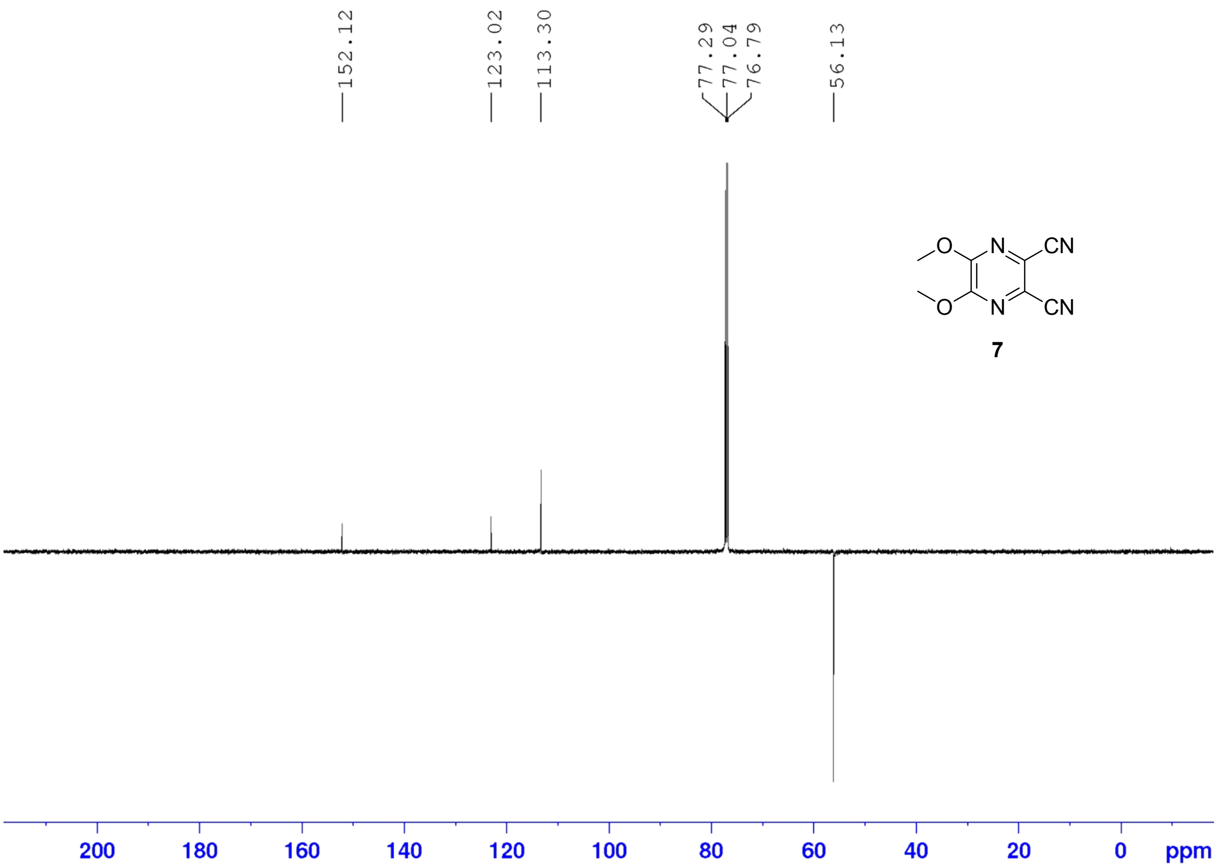

Figure S59. ^{13}C NMR APT spectrum of chromophore **7** (125 MHz, $CDCl_3$, 25 °C).

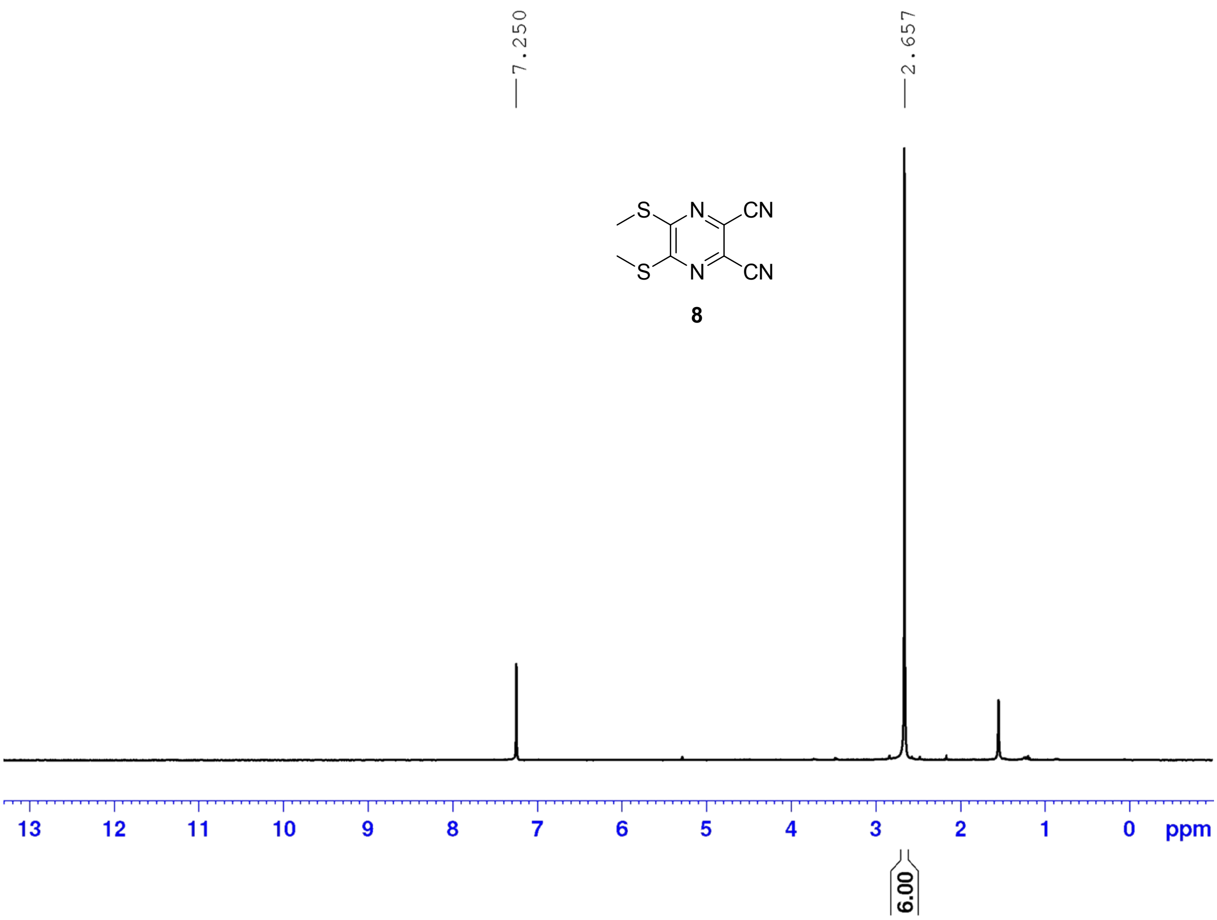

Figure S60. ^{1}H NMR spectrum of chromophore **8** (400 MHz, $CDCl_3$, 25 °C).

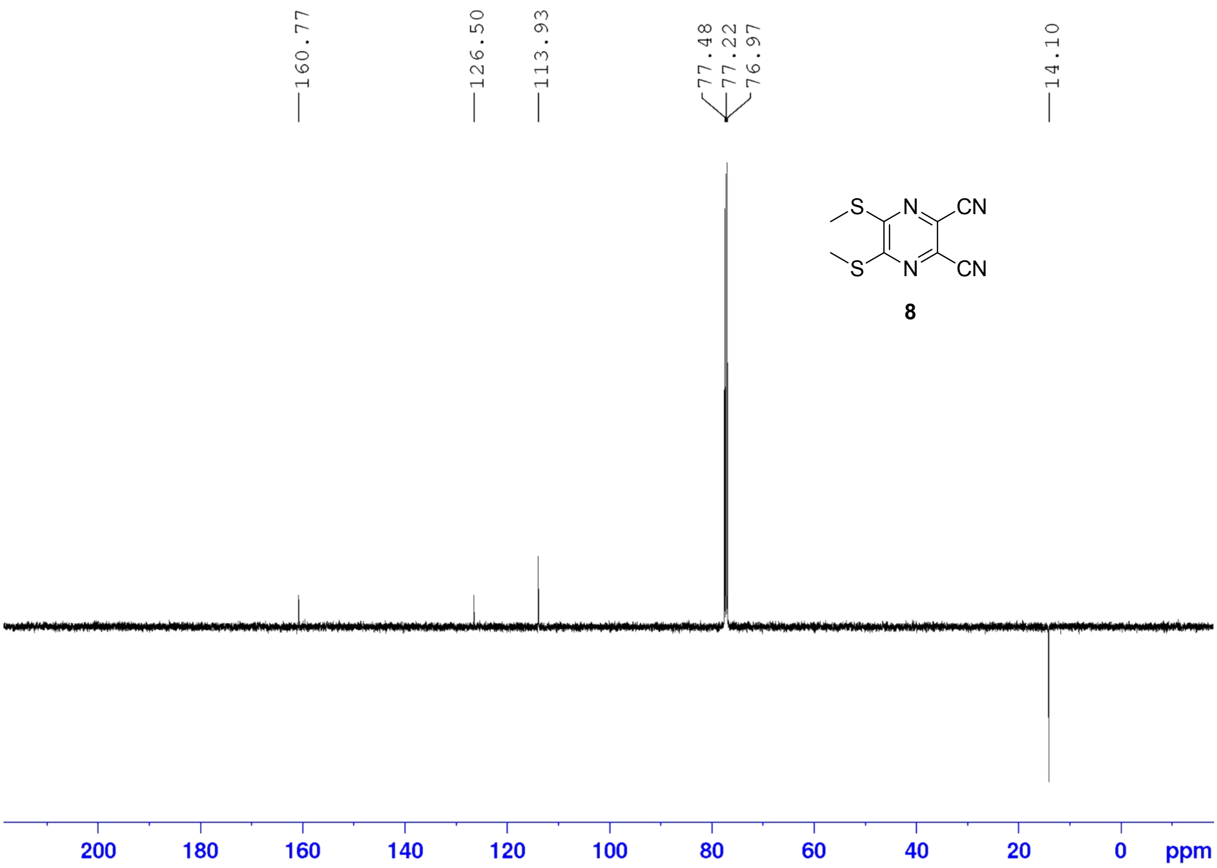

Figure S61. ^{13}C NMR APT spectrum of chromophore **8** (125 MHz, $CDCl_3$, 25 °C).

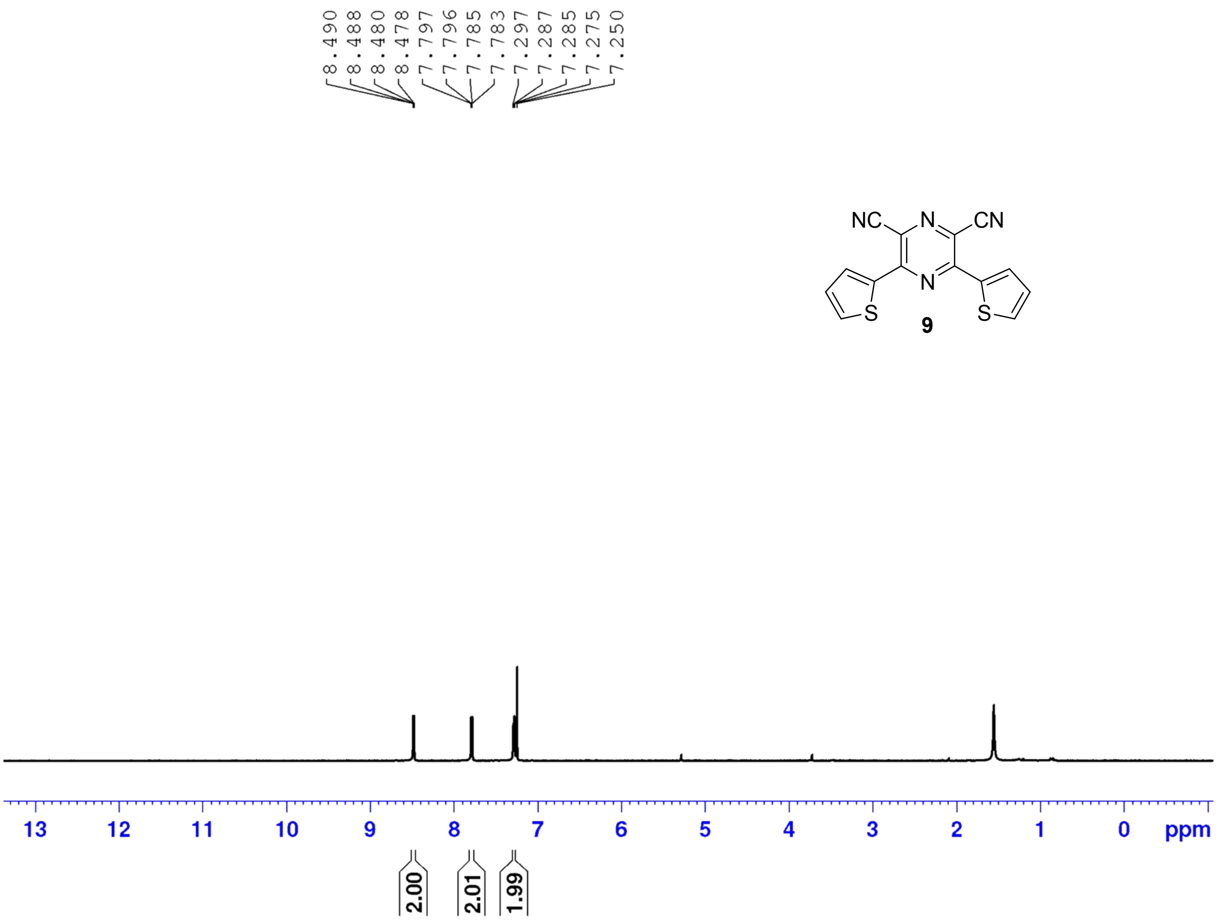

Figure S62. ^1H NMR spectrum of chromophore **9** (400 MHz, $CDCl_3$, 25 °C).

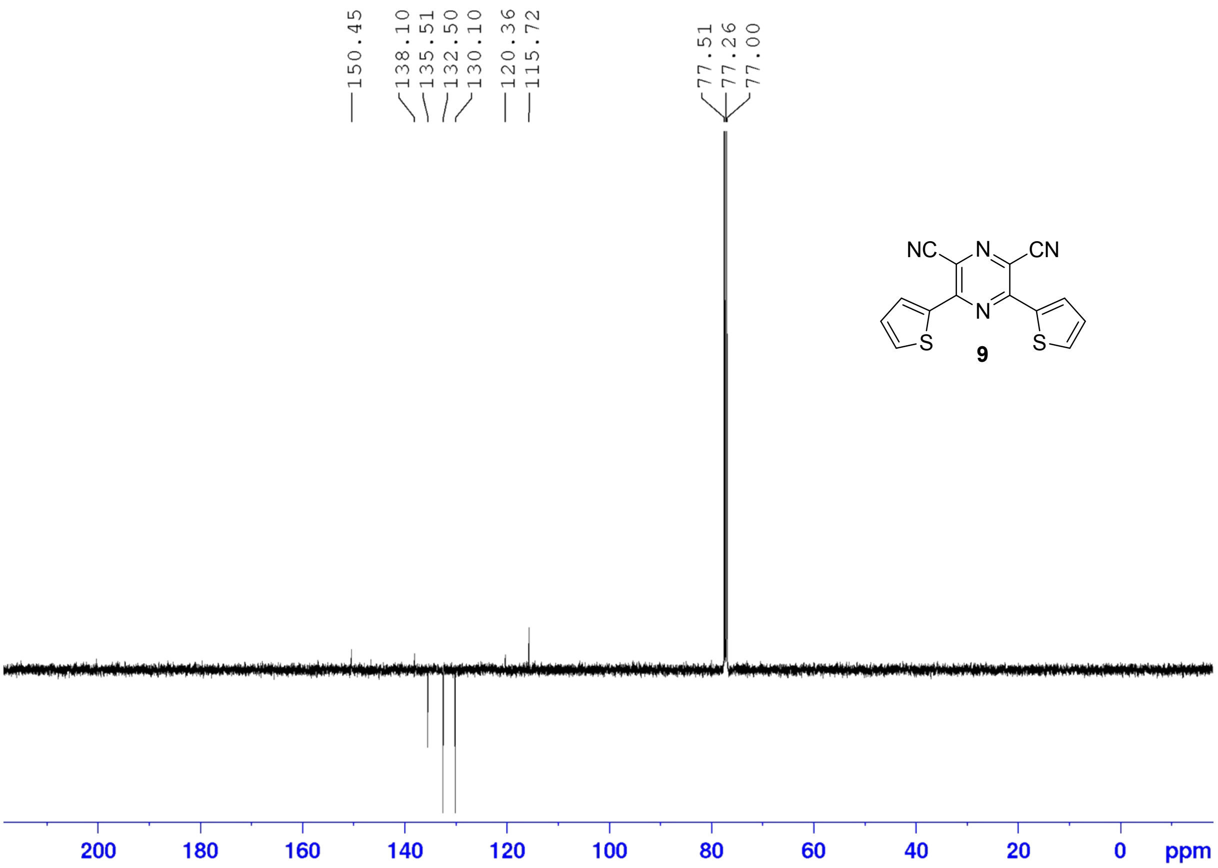

Figure S63. ^{13}C NMR APT spectrum of chromophore **9** (125 MHz, $CDCl_3$, 25 °C).

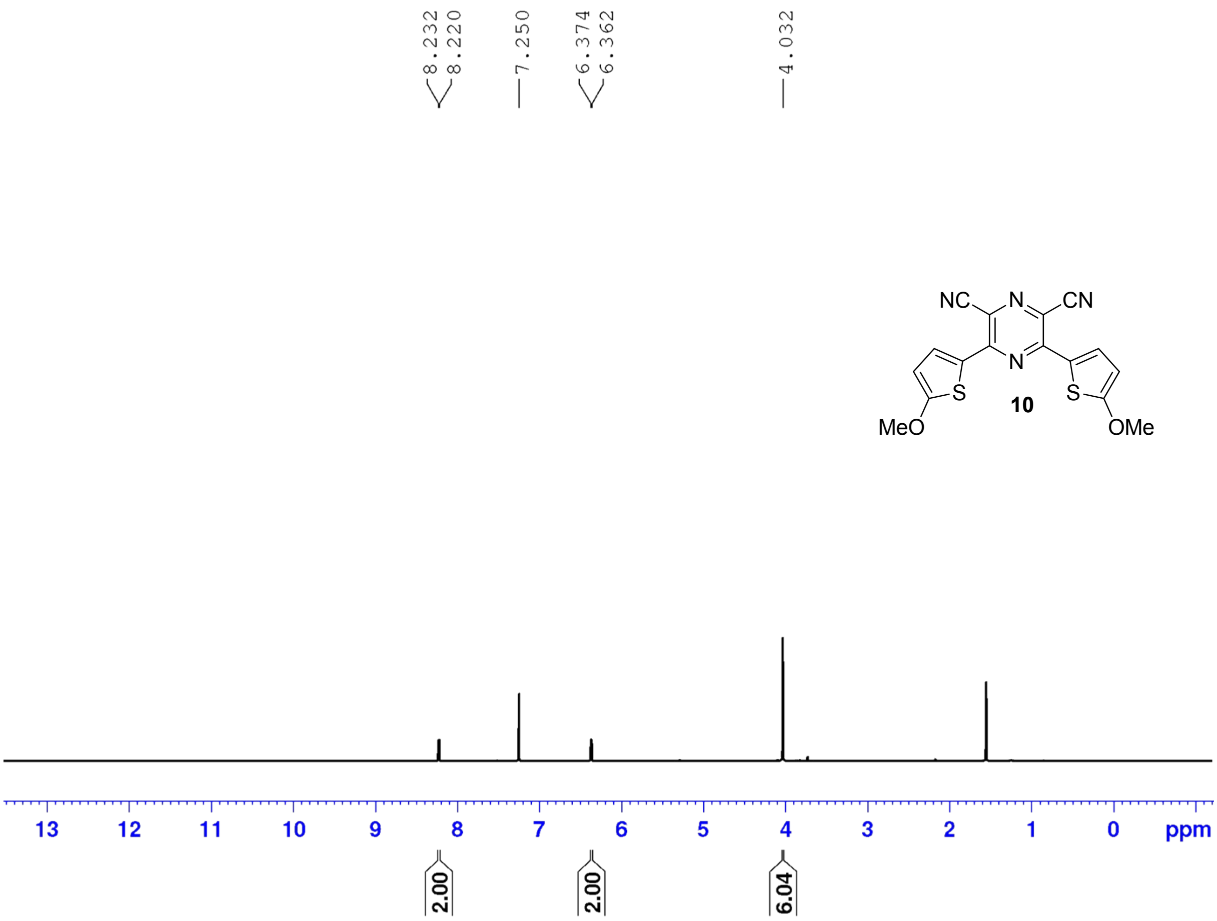

Figure S64. ^{1}H NMR spectrum of product **10** (400 MHz, $CDCl_3$, 25 °C).

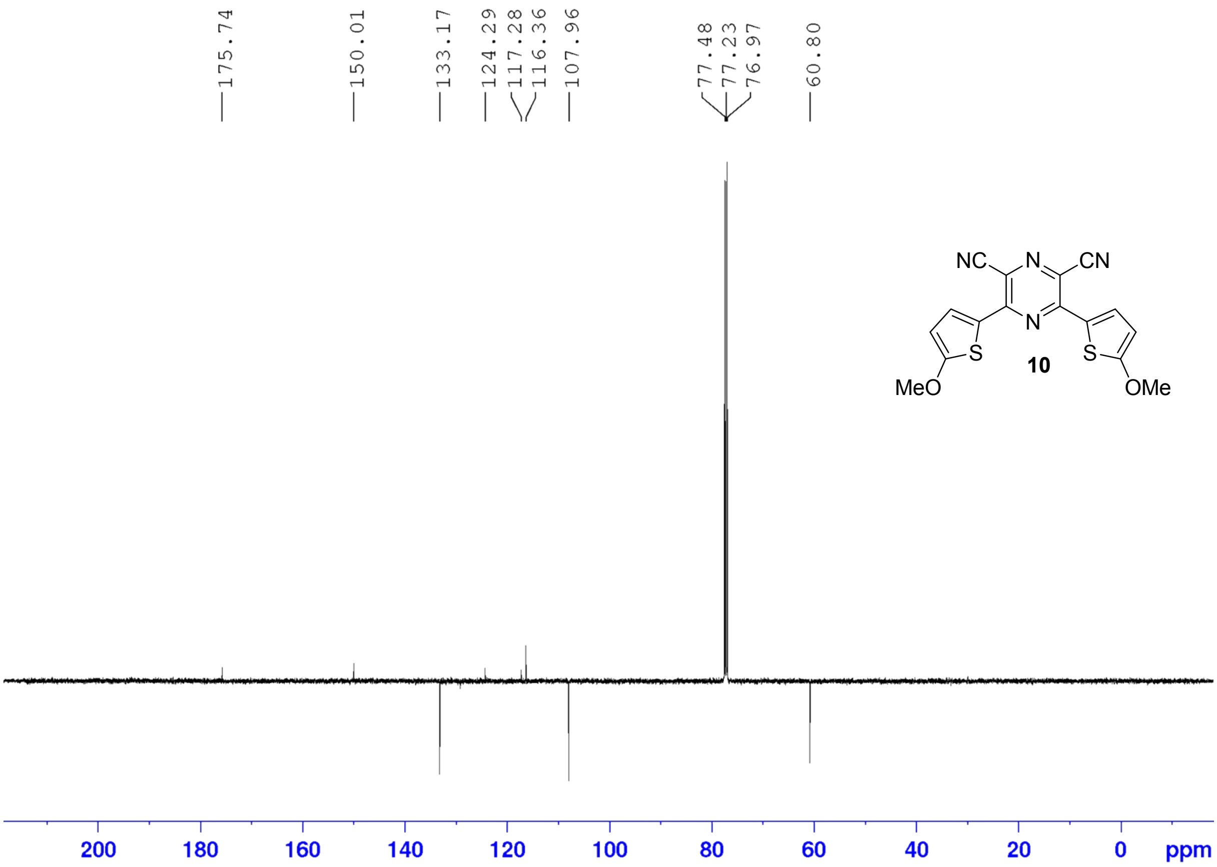

Figure S65. ^{13}C NMR spectrum of product **10** (125 MHz, $CDCl_3$, 25 °C).

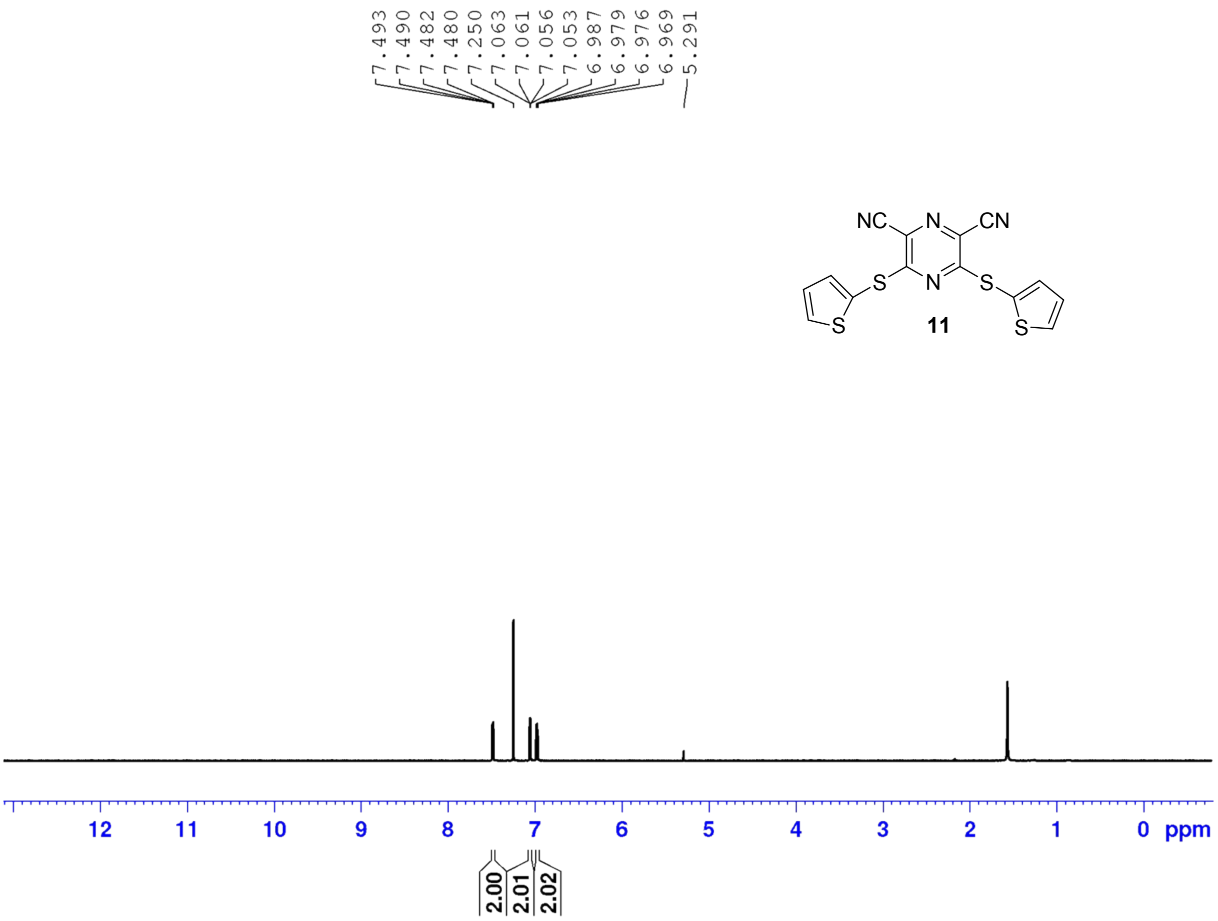

Figure S66. ^{1}H NMR spectrum of product **11** (500 MHz, $CDCl_3$, 25 °C).

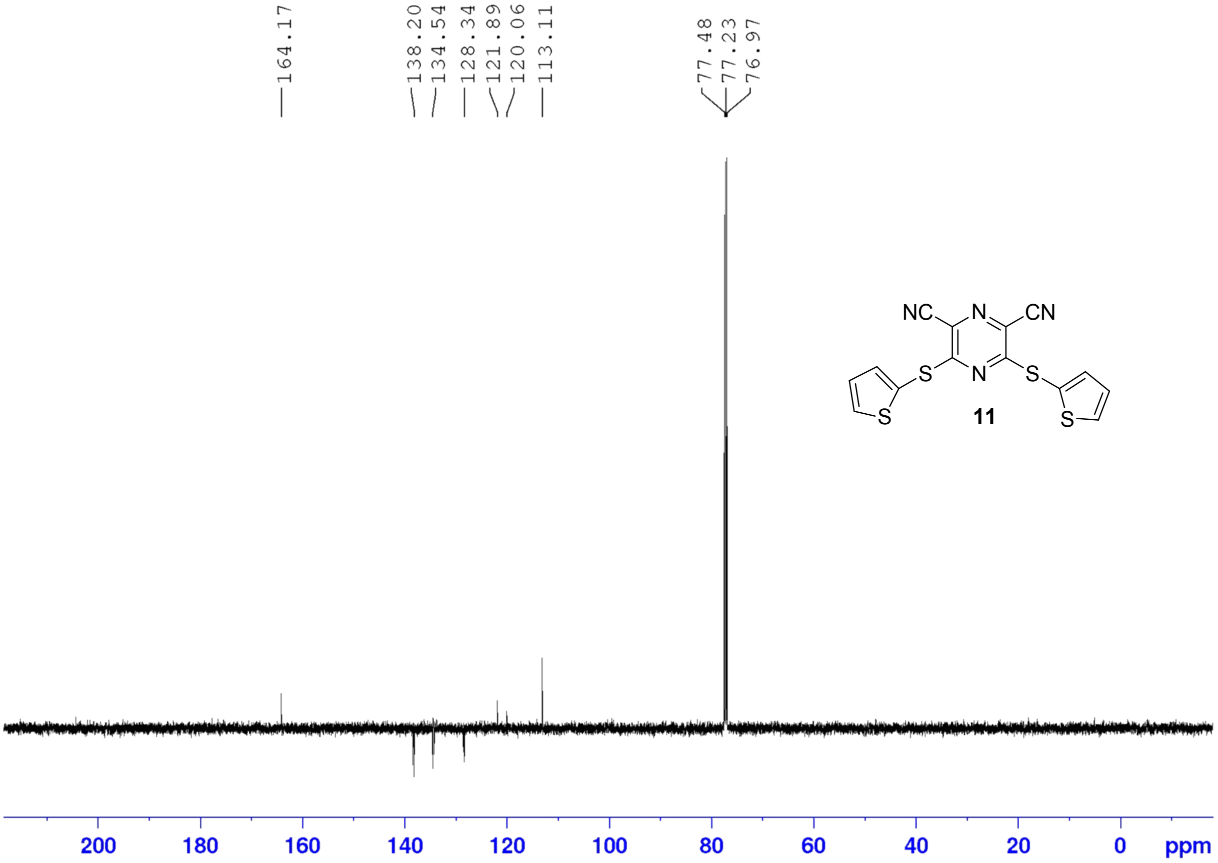

Figure S67. ^{13}C NMR spectrum of product **11** (125 MHz, $CDCl_3$, 25 °C).

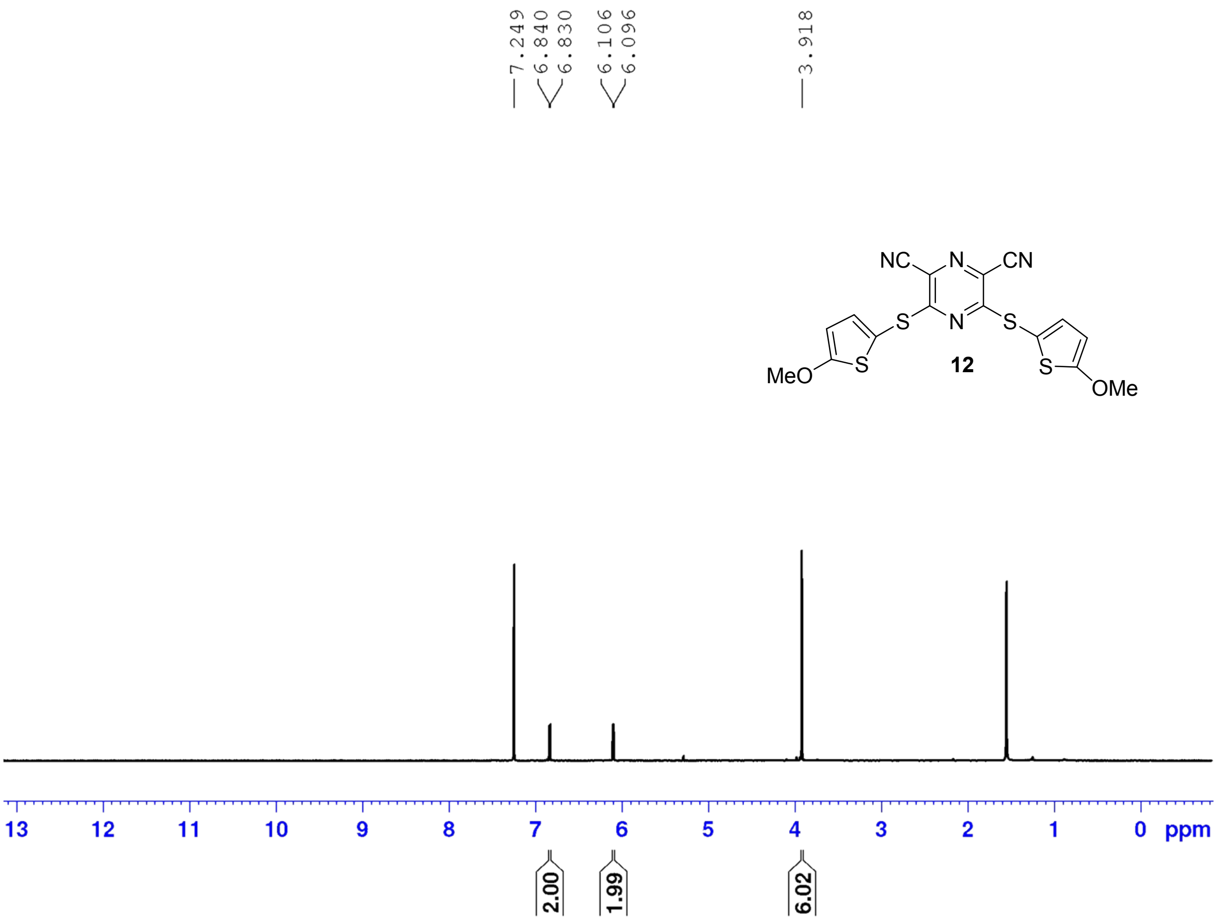

Figure S68. ^{1}H NMR spectrum of product 1**2** (400 MHz, $CDCl_3$, 25 °C).

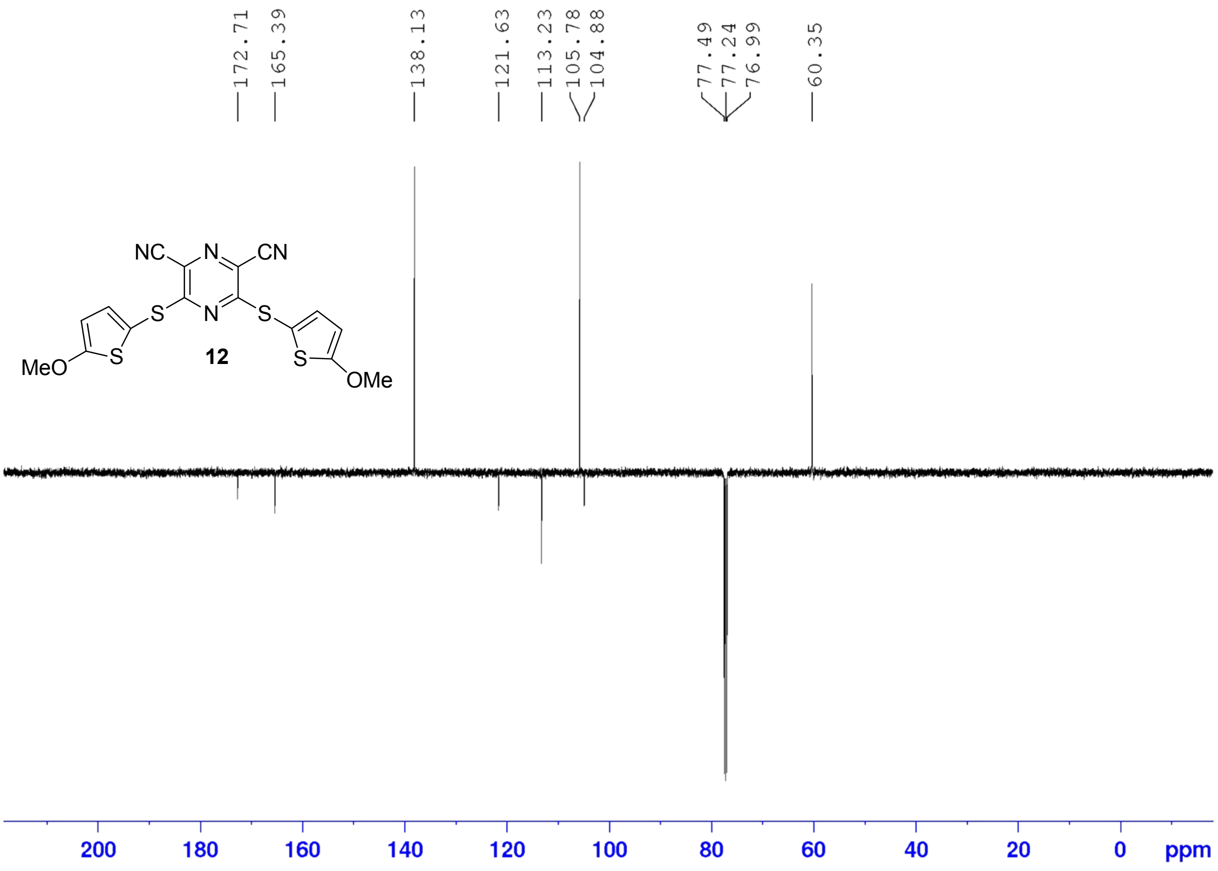

Figure S69. ^{13}C NMR spectrum of product **12** (125 MHz, $CDCl_3$, 25 °C).

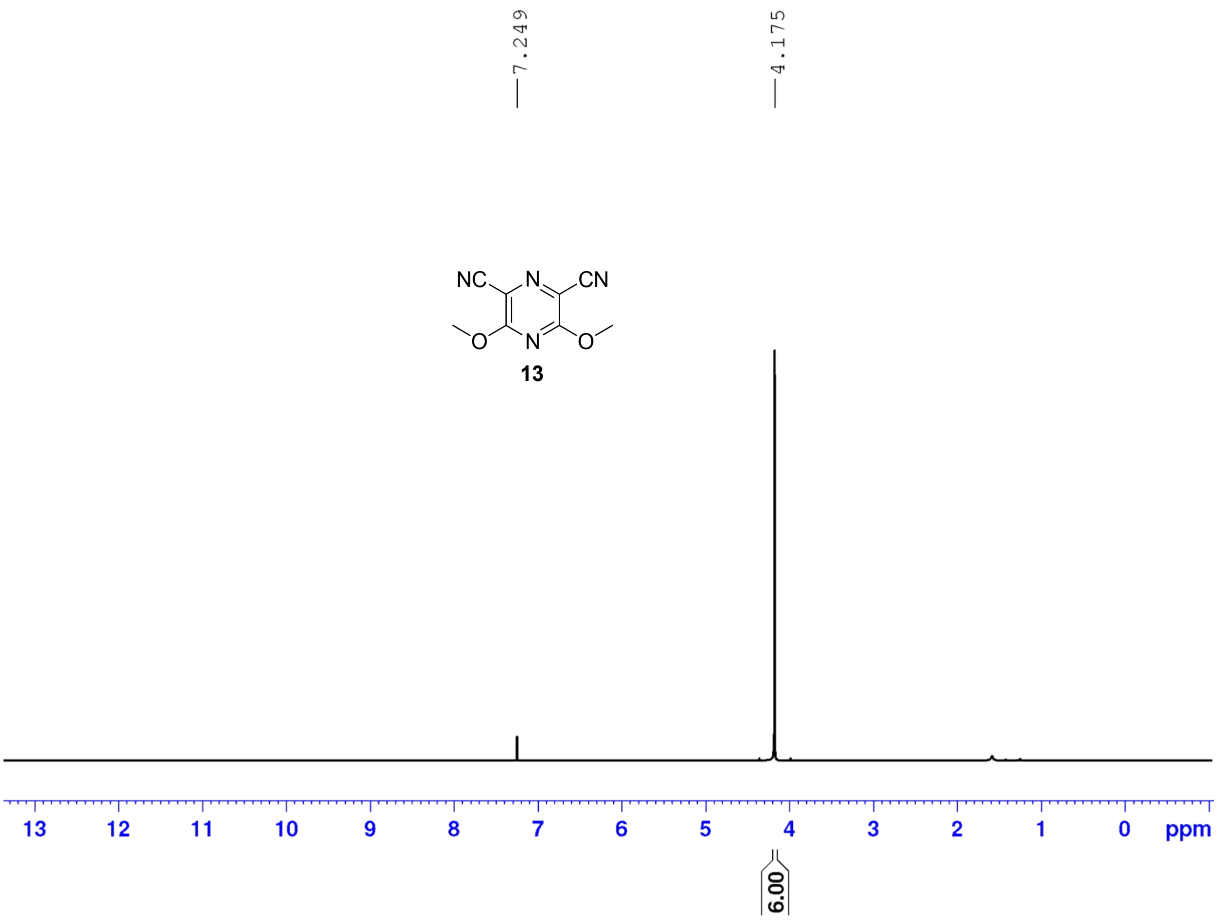

Figure S70. ^{1}H NMR spectrum of product **13** (400 MHz, $CDCl_3$, 25 °C).

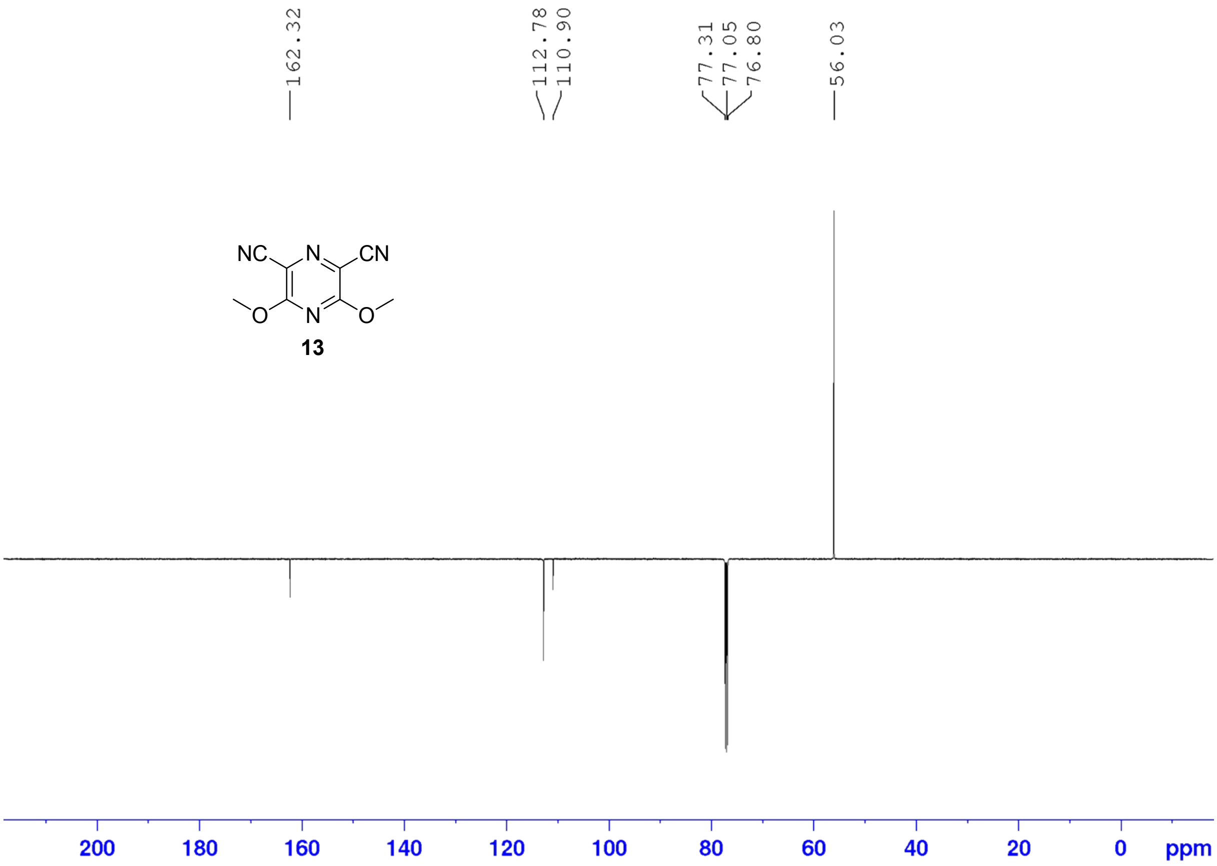

Figure S71. ^{13}C NMR spectrum of product **13** (125 MHz, $CDCl_3$, 25 °C).

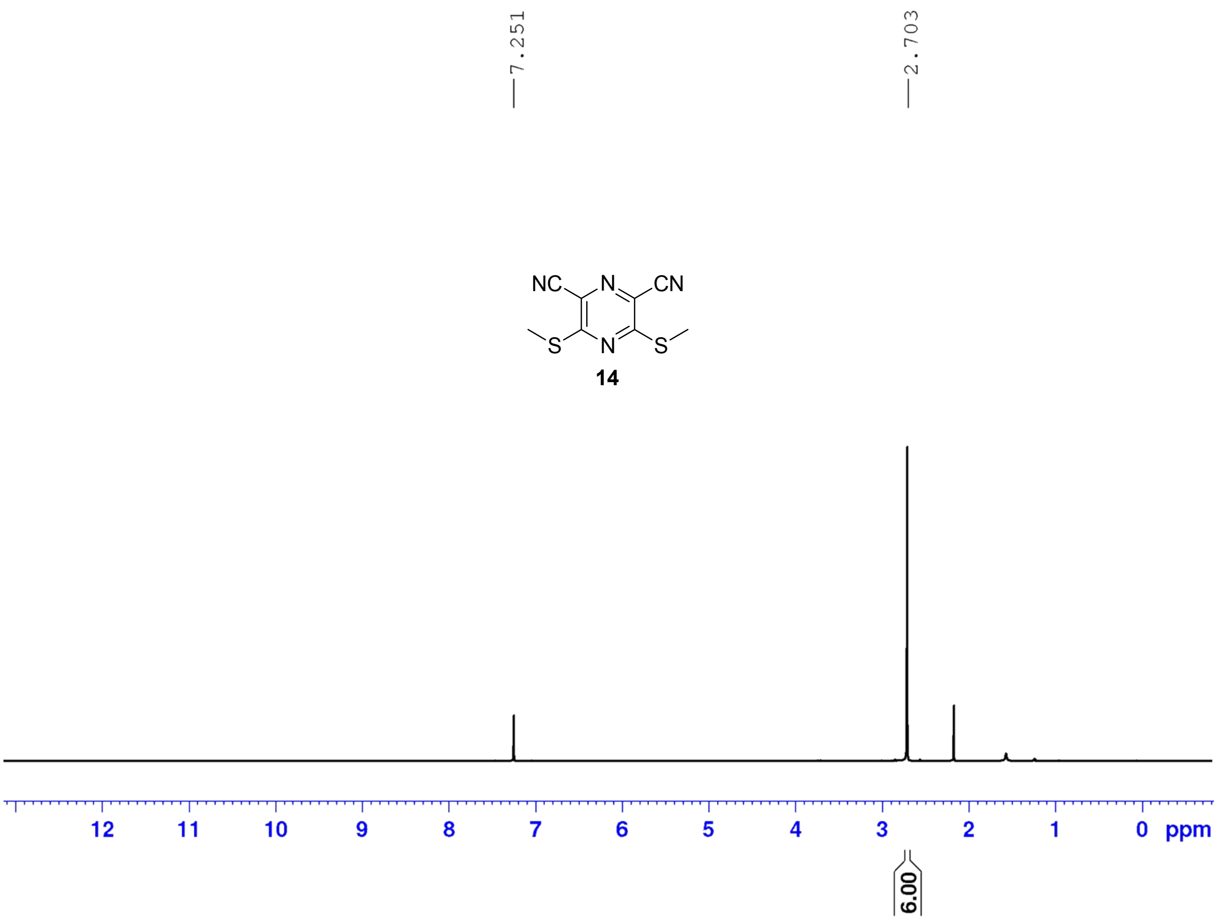

Figure S72. ^{1}H NMR spectrum of product **14** (500 MHz, $CDCl_3$, 25 °C).

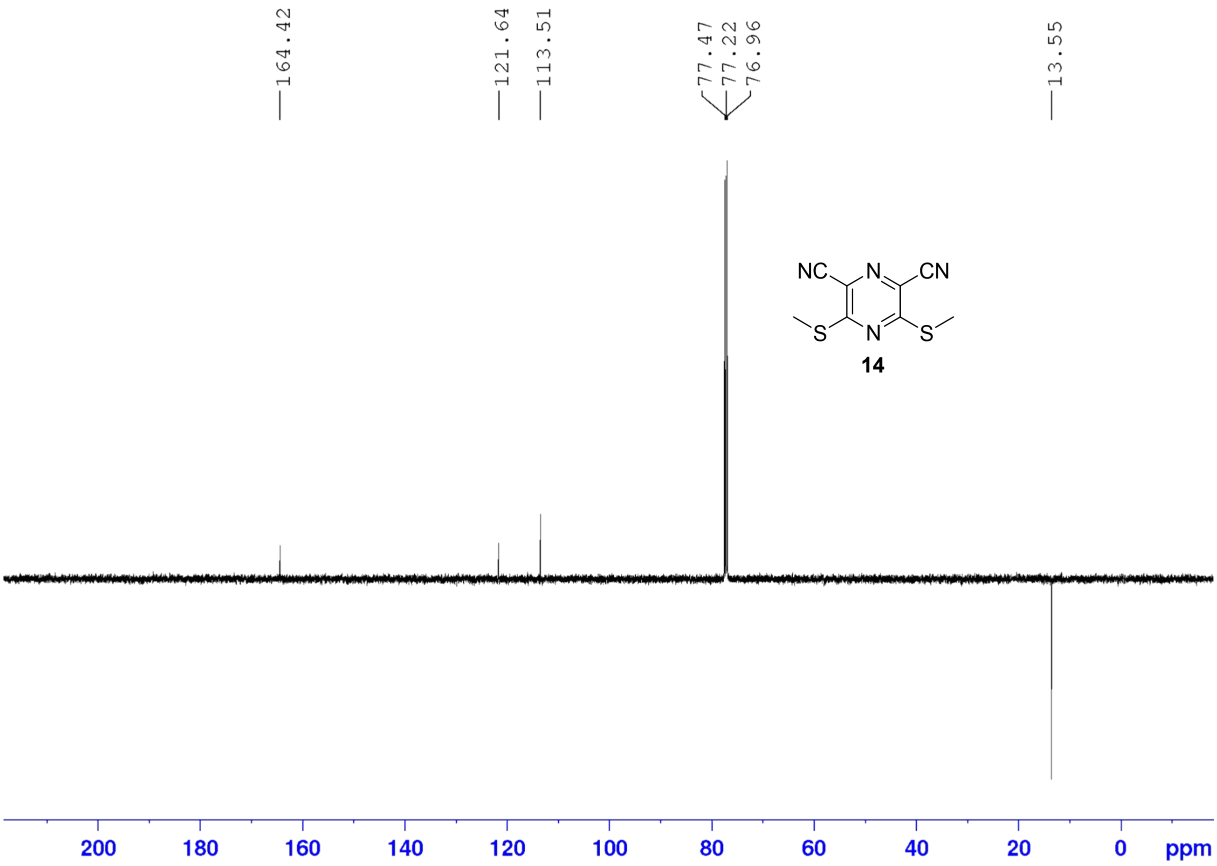

Figure S73. ^{13}C NMR spectrum of product **14** (125 MHz, $CDCl_3$, 25 °C).

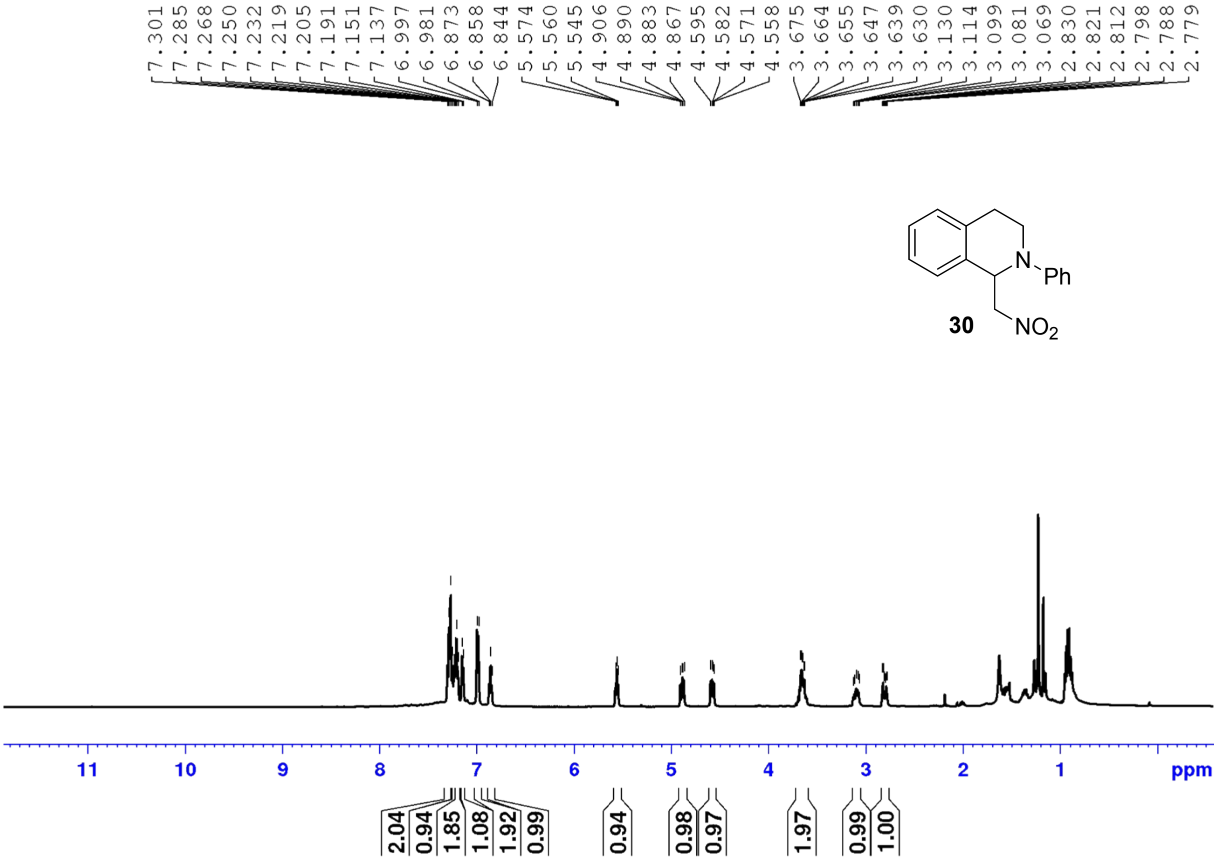

Figure S73. ^{1}H NMR spectrum of product **30** (500 MHz, $CDCl_3$, 25 °C).

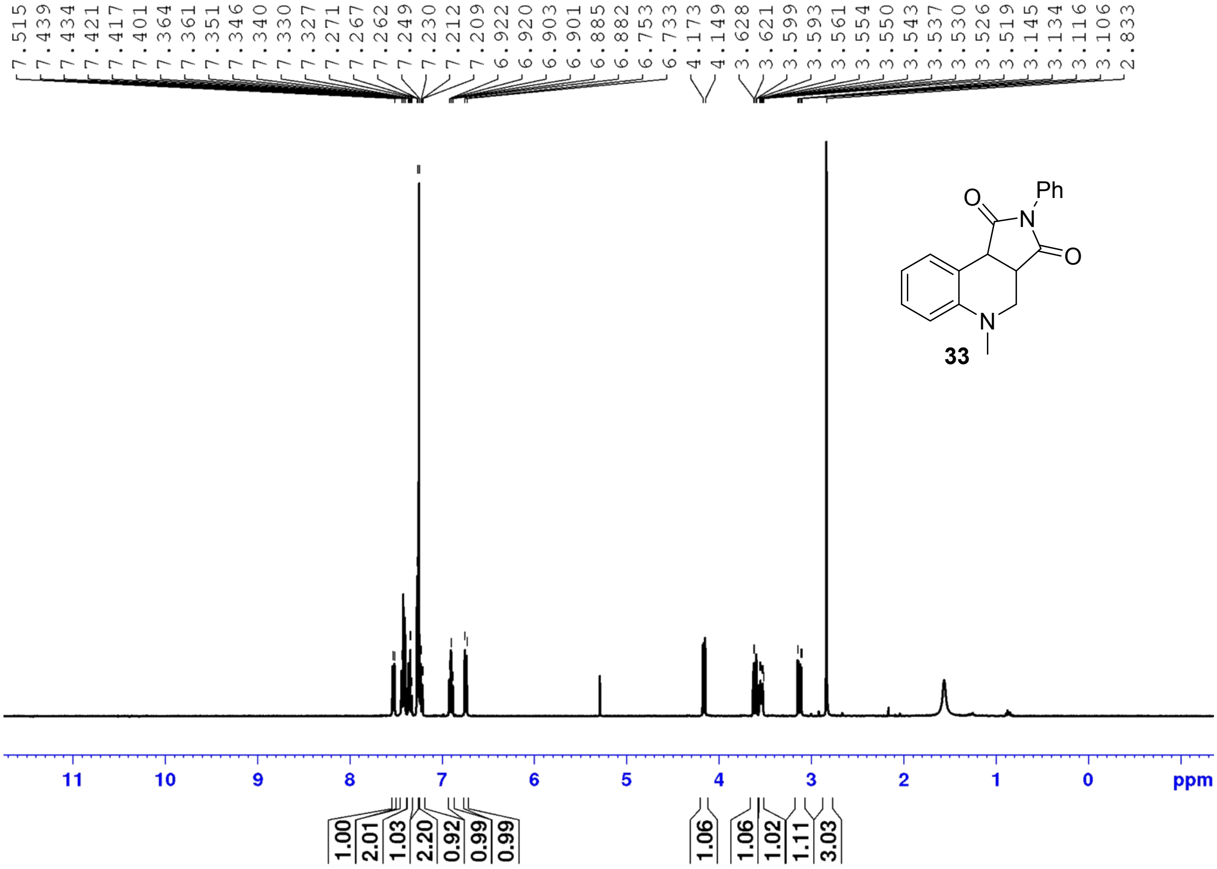

Figure S73. ^{1}H NMR spectrum of product **33** (400 MHz, $CDCl_3$, 25 °C).

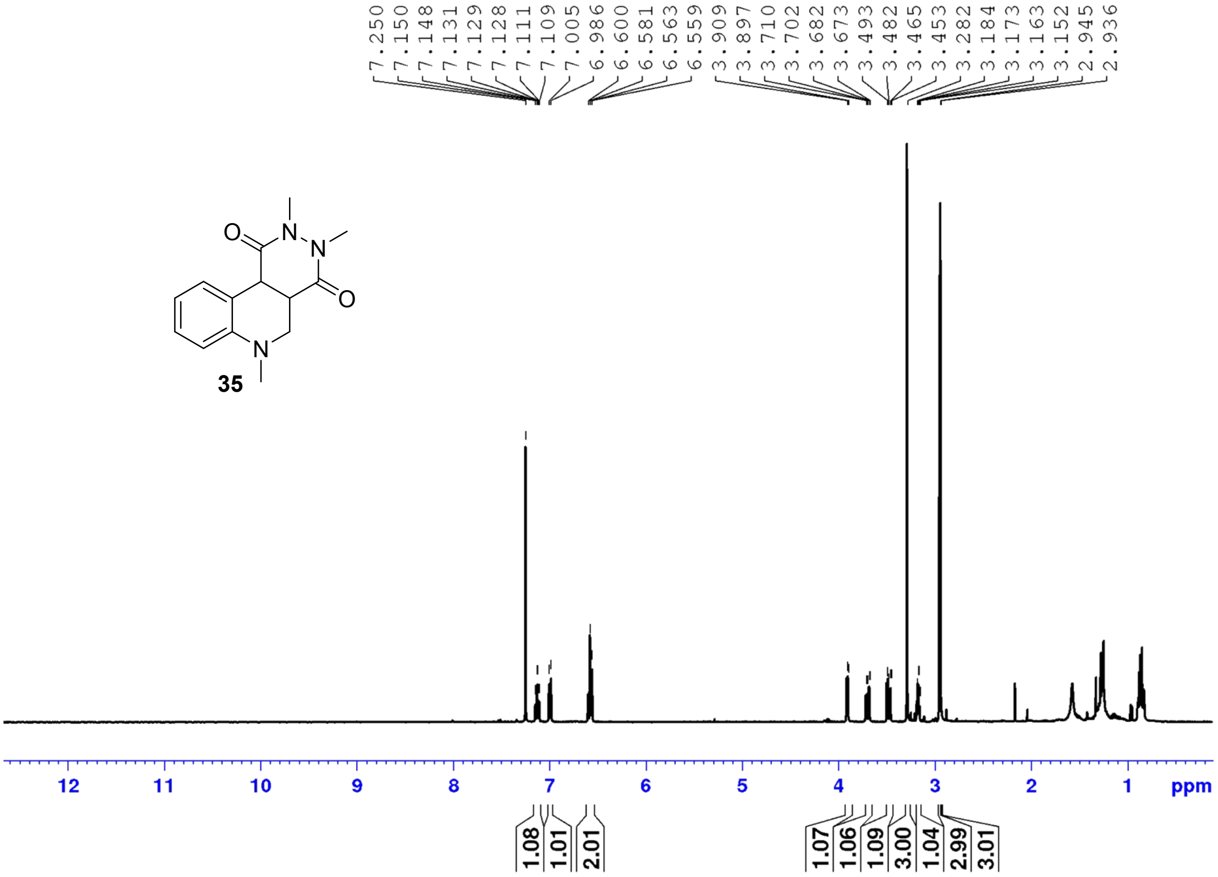

Figure S73. ^{1}H NMR spectrum of product **35** (400 MHz, $CDCl_3$, 25 °C).

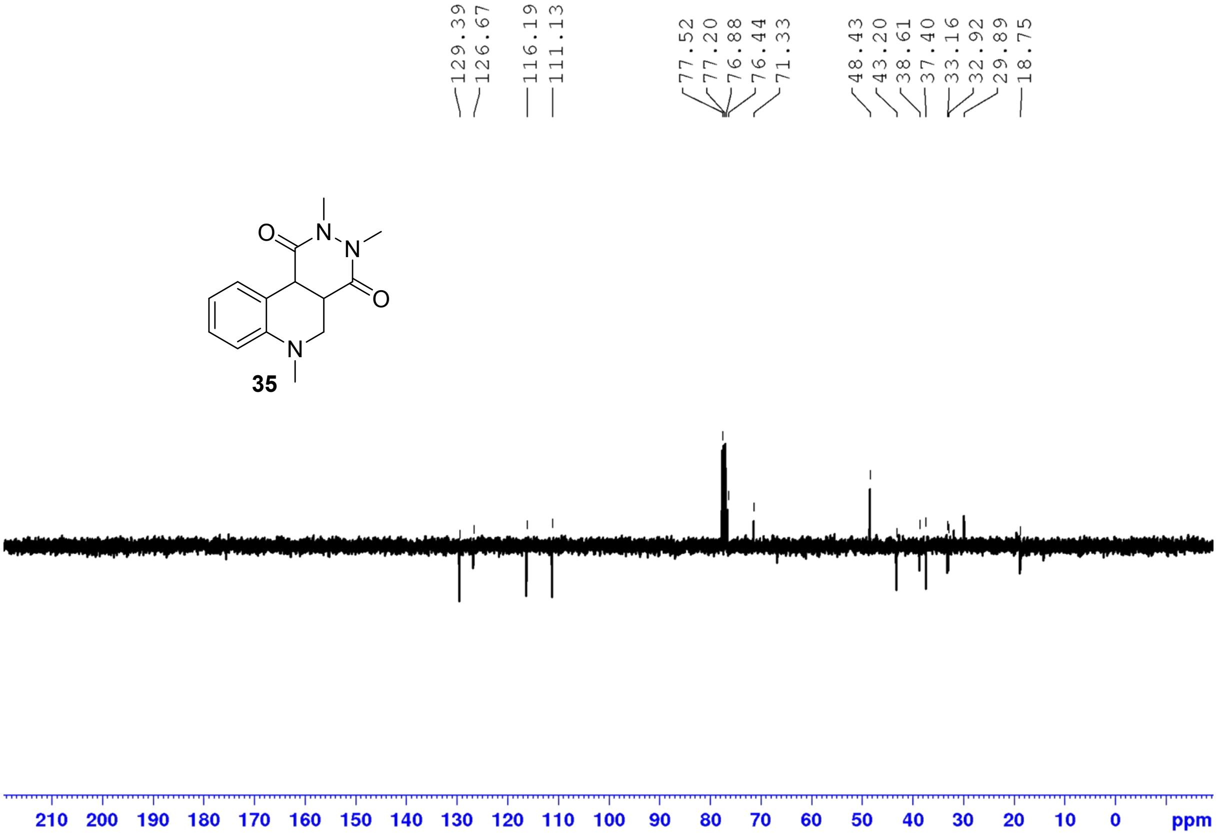

Figure S73. ^{13}C NMR spectrum of product **35** (100 MHz, $CDCl_3$, 25 °C).

8. References

1. L. Brandsma, M. A. Keegstra, H. A. Theo, *Tetrahedron* **1992**, *48*, 3633.
2. J. Wang, M. Frings, C. Bolm, *Angew. Chem. Int. Ed.* **2013**, *125*, 8823.
3. T. Furukawa, M. Tobisu, N. Chatani, *J. Am. Chem. Soc*. **2015**, *137*, 12211.
4. M. Zambianchi, F. Di Maria, A. Cazzato, G. Gigli, M. Piacenza, F. Della Sala, G. Barbarella, *J. Am. Chem. Soc.* **2009**, *131*, 10892.
5. M. F. Fathalla, S. H. Khattab, *J. Chem. Soc. Pak.* **2011**, *33*, 324.
6. G. Kinast, *Liebigs Ann. Chem.* **1981**, 1561.
7. J. Perchais, J. P. Fleury, *Tetrahedron* **1974**, *30*, 999.
8. D. Vázquez Vilarelle, C. Peinador Veira, J. M. Quintela López, *Tetrahedron* **2004**, *60*, 275.
9. K. Eichenberg, A. Staehelin, J. Druey, *Helv. Chim. Acta*, **1954**, *37*, 837.
10. Q. Y. Meng, Q. Liu, J. J. Zhong, H. H. Zhang, Z. J. Li, B. Chen, C. H. Tung, L. Z. Wu, *Org. Lett.*, **2012**, *14*, 5992.
11. M. Hosseini-Sarvari, M. Koohgard, S. Firoozi, A. Mohajeri, H. Tavakolian, *New J. Chem.*, **2018**, *42*, 6880.
12. G.M. Sheldrick, *Acta Cryst. A* **2015**, *71*, 3.
